# Supplementary material for: Rapid room-temperature phosphorescence chiral recognition of natural amino acids
Source: Nat Commun. 2024 Apr 17;15:3314. doi: 10.1038/s41467-024-47648-z (PMC11024135; doi:10.1038/s41467-024-47648-z)
Supplement: Supplementary file 1 — Supplementary Information [file 41467_2024_47648_MOESM1_ESM.pdf]

Supplementary Information

Rapid Room-Temperature Phosphorescence Chiral Recognition of Natural Amino Acids

Xiaoyu Chen[1], Renlong Zhu[1], Baicheng Zhang[1], Xiaolong Zhang[1], Aoyuan Cheng[1], Hongping Liu[1], Ruiying Gao[2], Xuepeng Zhang[1], Biao Chen[1]*, Shuji Ye[1], Jun Jiang[1] and Guoqing Zhang[1,3]*

[1]Hefei National Research Center for Physical Sciences at the Microscale, University of Science and Technology of China, Anhui 230026, PR China.

[2]School of Chemistry and Materials Science, University of Science and Technology of China, Anhui 230026, PR China.

[3]Hefei National Laboratory, University of Science and Technology of China, Anhui 230094, PR China.

*E-mail: gzhang@ustc.edu.cn; biaochen@ustc.edu.cn

Table of Contents

1. Supplementary Methods........3

2. Supplementary Figures and Tables........8

1. Supplementary Methods

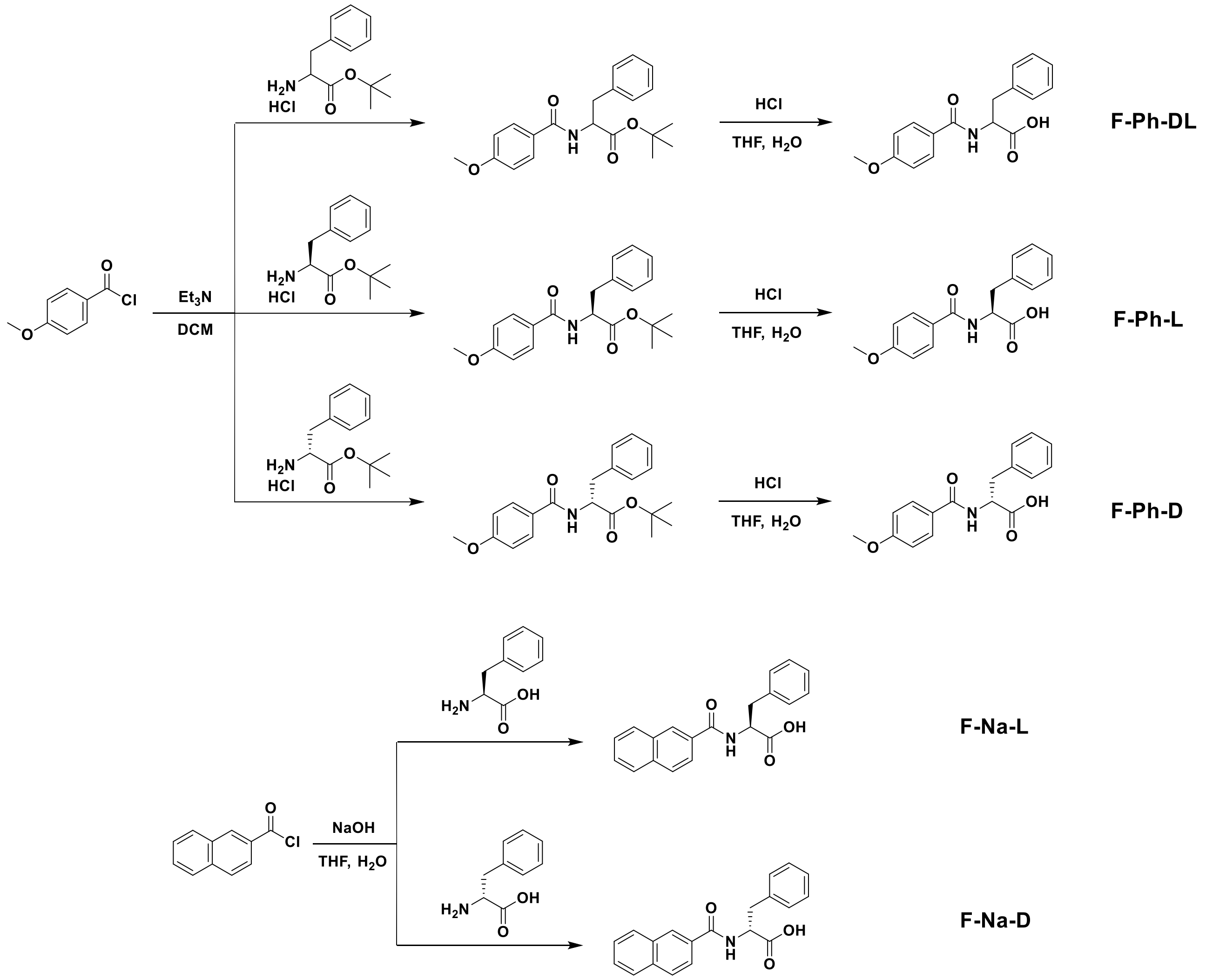

Supplementary Figure 1. Synthetic routes of the target compounds.

Preparation of (4-methoxybenzoyl)phenylalanine (F-Ph-DL)

Tert-butyl phenylalaninate hydrochloride (2.58 g, 10 mmol) and triethylamine (2.23 g, 22 mmol) were added to a round-bottomed flask containing 50 ml CH_2Cl_2 and the solution was stirred at 0 °C for 10 mins. Then 4-methoxybenzoyl chloride (1.88 g, 11 mmol) was added dropwise and the solution was stirred at 0 °C for an additional 1 h. Subsequently, the mixture was allowed to warm to room temperature and stirred overnight. The mixture was washed with 1 M HCl solution (25 ml*2) and the organic layer was dried over sodium sulfate and filtered, and the solvent was removed under vacuum, yielding a yellow viscous liquid. The crude product was dissolved in 30 ml THF and 25 ml 1 M HCl solution and stirred at 70 °C for 30 h. Then the mixture was extracted with EtOAc, the organic layer was dried over sodium sulfate and filtered, and the solvent was removed under vacuum. The product was washed with a mixture of 20 ml $CHCl_3$ and 40 ml Hexane and filtered, and the solvent was removed under vacuum, yielding pure **F-Ph-DL** as a white solid (2.36 g, 79%). ^{1}H NMR (400 MHz, DMSO-d6) δ = 12.68 (s, 1H), 8.54 (d, J = 8.1 Hz, 1H), 7.87-7.66 (m, 2H), 7.39-7.07 (m, 5H), 7.04-6.85 (m, 2H), 4.58 (ddd, J = 10.6, 8.2, 4.5 Hz, 1H), 3.80 (s, 3H), 3.17 (dd, J = 13.8, 4.6 Hz, 1H), 3.06 (dd, J = 13.8, 10.6 Hz, 1H). ^{13}C NMR (101

MHz, DMSO) δ = 173.81, 166.29, 162.15, 138.73, 129.67, 129.51, 128.63, 126.78, 126.61, 113.92, 55.81, 54.63, 40.64, 40.44, 40.23, 40.02, 39.81, 39.60, 39.39, 36.78. HRMS (EI+) m/z 300.12306 [$(M)^+$; calculated mass for $C_{17}H_{18}NO_4^+$: 300.12303 amu]. Elemental analysis (calcd., found for $C_{17}H_{17}NO_4$): C (68.22, 68.17), H (5.72, 5.76).

Preparation of (4-methoxybenzoyl)phenylalanine (F-Ph-L)

The same synthesis method as **F-Ph-DL**, tert-butyl L-phenylalaninate hydrochloride (2.56 g, 9.9 mmol), and 4-methoxybenzoyl chloride (1.88 g, 11 mmol) afforded **F-Ph-L** (1.88 g, 63%) as a white solid. ^{1}H NMR (400 MHz, DMSO-d6) δ = 12.68 (s, 1H), 8.54 (d, *J* = 8.1 Hz, 1H), 7.87-7.66 (m, 2H), 7.39-7.07 (m, 5H), 7.04-6.85 (m, 2H), 4.58 (ddd, *J* = 10.6, 8.2, 4.5 Hz, 1H), 3.80 (s, 3H), 3.17 (dd, *J* = 13.8, 4.6 Hz, 1H), 3.06 (dd, *J* = 13.8, 10.6 Hz, 1H). ^{13}C NMR (101 MHz, DMSO) δ = 173.81, 166.29, 162.15, 138.73, 129.66, 129.51, 128.63, 126.78, 126.61, 113.92, 55.81, 54.63, 40.64, 40.43, 40.23, 40.02, 39.81, 39.60, 39.39, 36.78. HRMS (EI+) m/z 300.12267 [$(M)^+$; calculated mass for $C_{17}H_{18}NO_4^+$: 300.12303 amu]. Elemental analysis (calcd., found for $C_{17}H_{17}NO_4$): C (68.22, 68.24), H (5.72, 5.75).

Preparation of (4-methoxybenzoyl)phenylalanine (F-Ph-D)

The same synthesis method as **F-Ph-DL**, tert-butyl D-phenylalaninate hydrochloride (2.57 g, 10 mmol), and 4-methoxybenzoyl chloride (1.88 g, 11 mmol) afforded **F-Ph-D** (1.92 g, 64%) as a white solid. ^{1}H NMR (400 MHz, DMSO-d6) δ = 12.69 (s, 1H), 8.54 (d, *J* = 8.1 Hz, 1H), 7.85-7.63 (m, 2H), 7.39-7.05 (m, 5H), 7.04-6.85 (m, 2H), 4.59 (ddd, *J* = 10.6, 8.1, 4.4 Hz, 1H), 3.80 (s, 3H), 3.17 (dd, *J* = 13.8, 4.5 Hz, 1H), 3.06 (dd, *J* = 13.8, 10.6 Hz, 1H). ^{13}C NMR (101 MHz, DMSO) δ = 173.80, 166.29, 162.15, 138.73, 129.66, 129.51, 128.63, 126.78, 126.61, 113.91, 55.81, 54.62, 40.64, 40.43, 40.23, 40.02, 39.81, 39.60, 39.39, 36.78. HRMS (EI+) m/z 300.12318 [$(M)^+$; calculated mass for $C_{17}H_{18}NO_4^+$: 300.12303 amu]. Elemental analysis (calcd., found for $C_{17}H_{17}NO_4$): C (68.22, 68.20), H (5.72, 5.69).

Preparation of (2-naphthoyl)-L-phenylalanine (F-Na-L)

L-Phenylalanine (1.65 g, 10 mmol) and sodium hydroxide solution (0.88 g in 8 ml H_2O, 22 mmol) were added to a round-bottomed flask containing 30 ml tetrahydrofuran and the solution was stirred at 0 °C for 10 mins. Then, 2-naphthoyl chloride (2.10 g, 11 mmol) was added and the solution was stirred at 0 °C for an additional 0.5 h. Subsequently, the mixture was allowed to warm to room temperature and stirred for 2 h. The mixture was washed with 30 ml HCl solution (1 M) and 50 ml ethyl acetate, the organic layer was dried over sodium sulfate and filtered, and the solvent was removed under vacuum, yielding a white solid. The crude product was washed with a mixture of 30 ml $CHCl_3$ and 30 ml Hexane and filtered, and the solvent was removed under vacuum, yielding pure **F-Na-L** as a white solid (2.86 g, 89%). ^{1}H NMR (400 MHz, DMSO-d6) δ = 12.81 (s, 1H), 8.90 (d, *J* = 8.1 Hz, 1H), 8.42 (s, 1H), 8.06-7.82 (m, 4H), 7.61 (tt, *J* = 7.0, 5.3 Hz, 2H), 7.40-7.12 (m, 5H), 4.68 (ddd, *J* = 10.3, 8.0, 4.4 Hz, 1H), 3.23 (dd, *J* = 13.8, 4.5 Hz, 1H), 3.12 (dd, *J* = 13.9, 10.5 Hz, 1H). ^{13}C NMR (101 MHz, DMSO) δ = 173.65, 166.83, 138.66, 134.66, 132.53, 131.75, 129.55, 129.31, 128.67, 128.31, 128.13, 128.09, 127.23, 126.83, 124.66, 54.75,

40.65, 40.44, 40.23, 40.02, 39.82, 39.61, 39.40, 36.84. HRMS (EI+) m/z 320.12754 [$(M)^+$; calculated mass for $C_{20}H_{18}NO_3^+$: 320.12812 amu]. Elemental analysis (calcd., found for $C_{20}H_{17}NO_3$): C (75.22, 75.24), H (5.37, 5.41).

Preparation of (2-naphthoyl)-L-phenylalanine (F-Na-D)

The same synthesis method as **F-Na-L**, D-phenylalanine (860 mg, 5.2 mmol), and 2-naphthoyl chloride (1.15 g, 6.0 mmol) afforded **F-Na-D** (1.49 g, 90%) as a white solid. ^{1}H NMR (400 MHz, DMSO-d6) δ = 12.81 (s, 1H), 8.89 (d, *J* = 8.1 Hz, 1H), 8.41 (d, *J* = 1.8 Hz, 1H), 8.05-7.84 (m, 4H), 7.61 (tt, *J* = 7.0, 5.2 Hz, 2H), 7.39-7.14 (m, 5H), 4.68 (ddd, *J* = 10.3, 8.1, 4.6 Hz, 1H), 3.23 (dd, *J* = 13.7, 4.5 Hz, 1H), 3.11 (dd, *J* = 13.8, 10.6 Hz, 1H). ^{13}C NMR (101 MHz, DMSO) δ = 173.65, 166.83, 138.66, 134.66, 132.53, 131.75, 129.55, 129.31, 128.67, 128.31, 128.13, 128.09, 127.23, 126.84, 124.66, 54.76, 40.65, 40.44, 40.23, 40.02, 39.82, 39.61, 39.40, 36.85. HRMS (EI+) m/z 320.12794 [$(M)^+$; calculated mass for $C_{20}H_{18}NO_3^+$: 320.12812 amu]. Elemental analysis (calcd., found for $C_{20}H_{17}NO_3$): C (75.22, 75.19), H (5.37, 5.35).

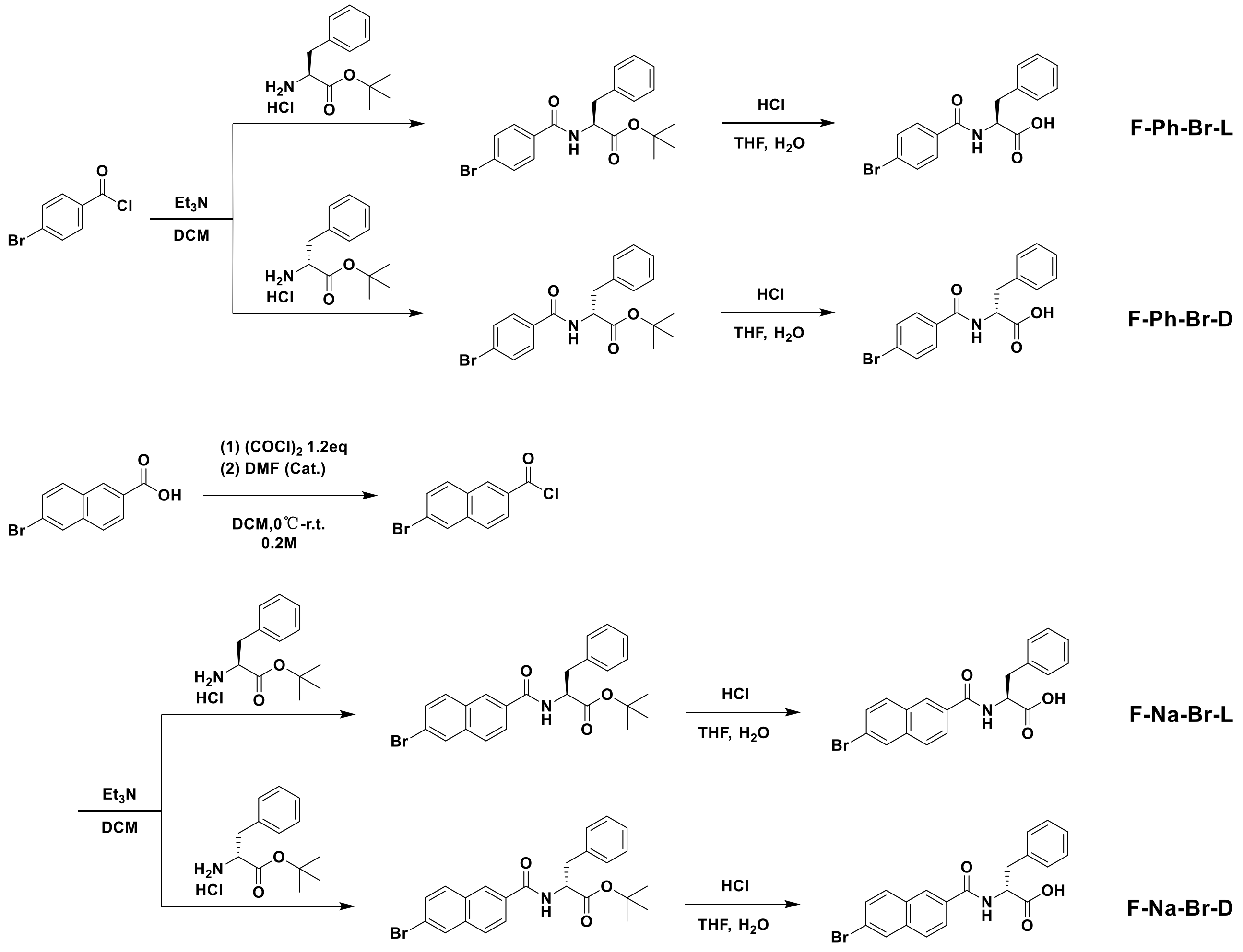

Supplementary Figure 2. Synthetic routes of the target compounds.

Preparation of (4-bromobenzoyl)-L-phenylalanine (F-Ph-Br-L)

Tert-butyl phenylalaninate hydrochloride (2.56 g, 9.9 mmol) and triethylamine (2.23 g, 22 mmol) were added to a round-bottomed flask containing 50 ml CH_2Cl_2 and the solution was stirred at 0 °C for 10 mins. Then 4-bromobenzoyl chloride (2.45 g, 11 mmol) was added dropwise and the solution was stirred at 0 °C for an additional 1 h. Subsequently, the mixture was allowed to warm to room temperature and stirred overnight. The mixture was washed with 1 M HCl solution (25 ml*2) and the organic layer was dried over sodium sulfate and filtered, and the solvent was removed under vacuum, yielding a yellow viscous liquid. The crude product was dissolved in 30 ml THF and 25 ml 1M HCl solution and stirred at 90 °C for 72 h. Then, the mixture was extracted with EtOAc, the organic layer was dried over sodium sulfate, filtered, and the solvent was removed under vacuum. The product was washed with a mixture of 20 ml $CHCl_3$ and 40 ml hexane twice and filtered and the solvent was removed under vacuum, yielding pure **F-Ph-Br-L** as a white solid (2.15 g, 62%). ^{1}H NMR (400 MHz, DMSO-d6) δ = 12.83 (s, 1H), 8.83 (d, *J* = 8.2 Hz, 1H), 7.79-7.63 (m, 4H), 7.34-7.14 (m, 5H), 4.60 (ddd, *J* = 10.7, 8.1, 4.3 Hz, 1H), 3.19 (dd, *J* = 13.8, 4.4 Hz, 1H), 3.05 (dd, *J* = 13.8, 10.8 Hz, 1H). ^{13}C NMR (101 MHz, DMSO) δ = 173.54, 165.88, 138.57, 133.40, 131.80, 129.93, 129.51, 128.67, 126.85, 125.64, 54.73, 40.59, 40.38, 40.17, 39.97, 39.76, 39.55, 39.34, 36.67. HRMS (EI+) m/z 348.02268 [$(M)^+$; calculated mass for $C_{16}H_{15}BrNO_3^+$: 348.02298 amu]. Elemental analysis (calcd., found for $C_{16}H_{14}BrNO_3$): C (55.19, 55.23), H (4.05, 4.02).

Preparation of (4-bromobenzoyl)-L-phenylalanine (F-Ph-Br-D)

The same synthesis method as **F-Ph-Br-L**, tert-butyl D-phenylalaninate hydrochloride (2.58 g, 10 mmol), and 4-bromobenzoyl chloride (2.45 g, 11 mmol) afforded **F-Ph-Br-D** (2.28 g, 66%) as a white solid. ^{1}H NMR (400 MHz, DMSO-d6) δ = 12.78 (s, 1H), 8.81 (d, *J* = 8.2 Hz, 1H), 7.78-7.56 (m, 4H), 7.35-7.13 (m, 5H), 4.61 (ddd, *J* = 10.7, 8.1, 4.4 Hz, 1H), 3.19 (dd, *J* = 13.8, 4.5 Hz, 1H), 3.05 (dd, *J* = 13.8, 10.7 Hz, 1H). ^{13}C NMR (101 MHz, DMSO) δ = 173.55, 165.88, 138.58, 133.41, 131.80, 129.93, 129.51, 128.67, 126.85, 125.64, 54.75, 40.59, 40.38, 40.17, 39.96, 39.76, 39.55, 39.34, 36.68. HRMS (EI+) m/z 348.02254 [$(M)^+$; calculated mass for $C_{16}H_{15}BrNO_3^+$: 348.02298 amu]. Elemental analysis (calcd., found for $C_{16}H_{14}BrNO_3$): C (55.19, 55.25), H (4.05, 4.07).

Preparation of (4-bromobenzoyl)-L-phenylalanine (F-Na-Br-L)

6-Bromo-2-naphthoic acid (2.09 g, 8.3 mmol) was added to a round-bottomed flask containing 70 ml anhydrous CH_2Cl_2 and the solution was stirred at 0 °C for 10 mins under inert gas protection. Then, oxalyl chloride (1.4 ml, 16.4 mmol) and anhydrous DMF (0.1 ml, 1.3 mmol) were added dropwise and the solution was stirred at 0 °C for an additional 1 h. Subsequently, the mixture was allowed to warm to room temperature and stirred for 3 h. The solvent was removed under vacuum, yielding a yellow solid. The crude product was dissolved in anhydrous 50 ml CH_2Cl_2, and tert-butyl phenylalaninate hydrochloride (2.04 g, 7.9 mmol) was added, and the solution was stirred at 0 °C for 10 mins under inert gas protection. Then, anhydrous triethylamine (3.5 ml, 25 mmol) was added dropwise and the solution was stirred at 0 °C for an additional 1 h. Subsequently, the mixture was allowed to warm to room

temperature and stirred for 20 h. The mixture was purified via silica gel chromatography and the product was dissolved in 20 ml of THF and 20 ml of 1 M HCl solution and stirred at 95 °C for 120 h. Then, the mixture was extracted with EtOAc, the organic layer was dried over sodium sulfate and filtered, and the solvent was removed under vacuum. The product was washed with a mixture of 20 ml $CHCl_3$ and 40 ml Hexane twice and filtered, and the solvent was removed under vacuum, yielding pure **F-Ph-Br-L** as a white solid (1.45 g, 46%). ^1H NMR (400 MHz, DMSO-d6) δ = 12.82 (s, 1H), 8.91 (d, *J* = 8.1 Hz, 1H), 8.42 (d, *J* = 1.6 Hz, 1H), 8.28 (d, *J* = 2.0 Hz, 1H), 8.04-7.86 (m, 3H), 7.71 (dd, *J* = 8.8, 2.0 Hz, 1H), 7.39-7.13 (m, 5H), 4.69 (ddd, *J* = 10.4, 8.0, 4.5 Hz, 1H), 3.23 (dd, *J* = 13.8, 4.6 Hz, 1H), 3.11 (dd, *J* = 13.9, 10.5 Hz, 1H). ^{13}C NMR (101 MHz, DMSO) δ = 173.63, 166.57, 138.61, 135.74, 132.27, 131.55, 131.07, 130.29, 130.07, 129.55, 128.70, 128.21, 127.64, 126.86, 125.81, 121.53, 54.80, 40.59, 40.38, 40.18, 39.97, 39.76, 39.55, 39.34, 36.79. HRMS (EI+) m/z 398.03859 [$(M)^+$; calculated mass for $C_{20}H_{17}BrNO_3^+$: 398.03863 amu]. Elemental analysis (calcd., found for $C_{20}H_{16}BrNO_3$): C (60.32, 60.28), H (4.05, 4.01).

Preparation of (4-bromobenzoyl)-L-phenylalanine (F-Na-Br-D)

The same synthesis method as **F-Na-Br-L**, tert-butyl D-phenylalaninate hydrochloride (2.00 g, 7.9 mmol), and 6-bromo-2-naphthoic acid (2.05 g, 8.2 mmol) afforded **F-Ph-Br-D** (1.52 g, 48%) as a white solid. ^1H NMR (400 MHz, DMSO-d6) δ = 12.82 (s, 1H), 8.91 (d, *J* = 8.1 Hz, 1H), 8.42 (d, *J* = 1.7 Hz, 1H), 8.28 (d, *J* = 2.0 Hz, 1H), 8.04-7.87 (m, 3H), 7.71 (dd, *J* = 8.8, 2.1 Hz, 1H), 7.39-7.14 (m, 5H), 4.68 (ddd, *J* = 10.5, 8.1, 4.5 Hz, 1H), 3.23 (dd, *J* = 13.8, 4.6 Hz, 1H), 3.11 (dd, *J* = 13.8, 10.5 Hz, 1H). ^{13}C NMR (101 MHz, DMSO) δ = 173.63, 166.57, 138.61, 135.74, 132.26, 131.55, 131.07, 130.29, 130.07, 129.55, 128.70, 128.21, 127.64, 126.87, 125.82, 121.53, 54.79, 40.59, 40.38, 40.17, 39.97, 39.76, 39.55, 39.34, 36.79. HRMS (EI+) m/z 398.03816 [$(M)^+$; calculated mass for $C_{20}H_{17}BrNO_3^+$: 398.03863 amu]. Elemental analysis (calcd., found for $C_{20}H_{16}BrNO_3$): C (60.32, 60.35), H (4.05, 4.03).

2. Supplementary Figures and Tables

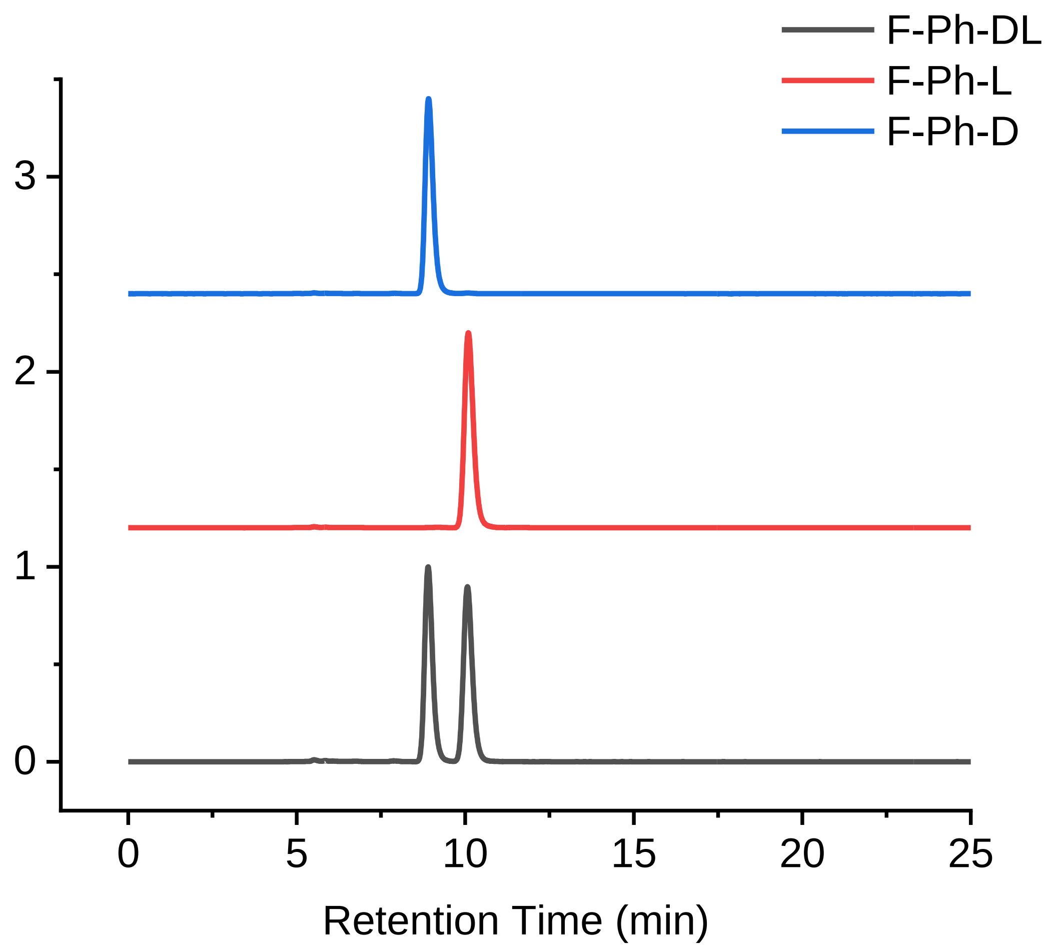

Supplementary Figure 3. Chiral high performance liquid chromatogram (Chiral HPLC) spectrum of the model compounds in ethanol (0.6 mg/ml) with ethanol monitored at the onset absorption of 285 nm.

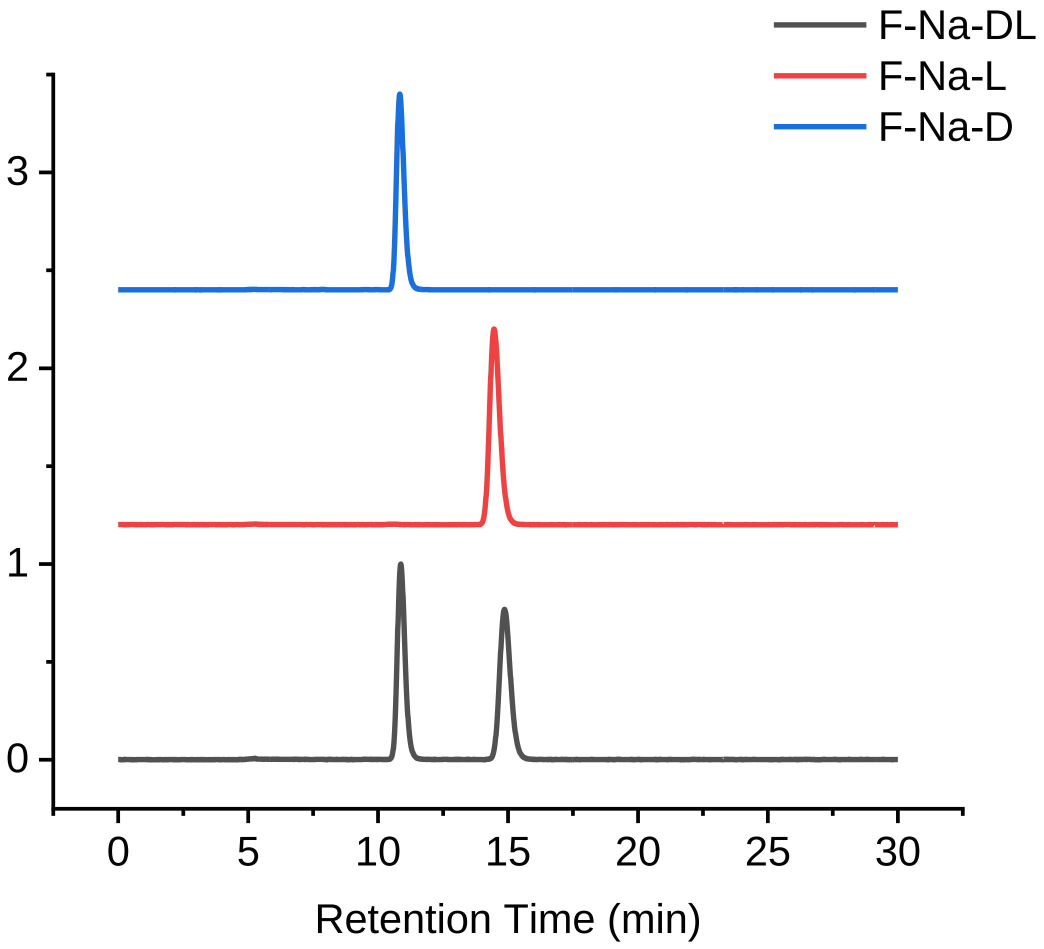

Supplementary Figure 4. Chiral high performance liquid chromatogram (Chiral HPLC) spectrum of the model compounds in ethanol (0.6 mg/ml) with ethanol monitored at the onset absorption of 337 nm.

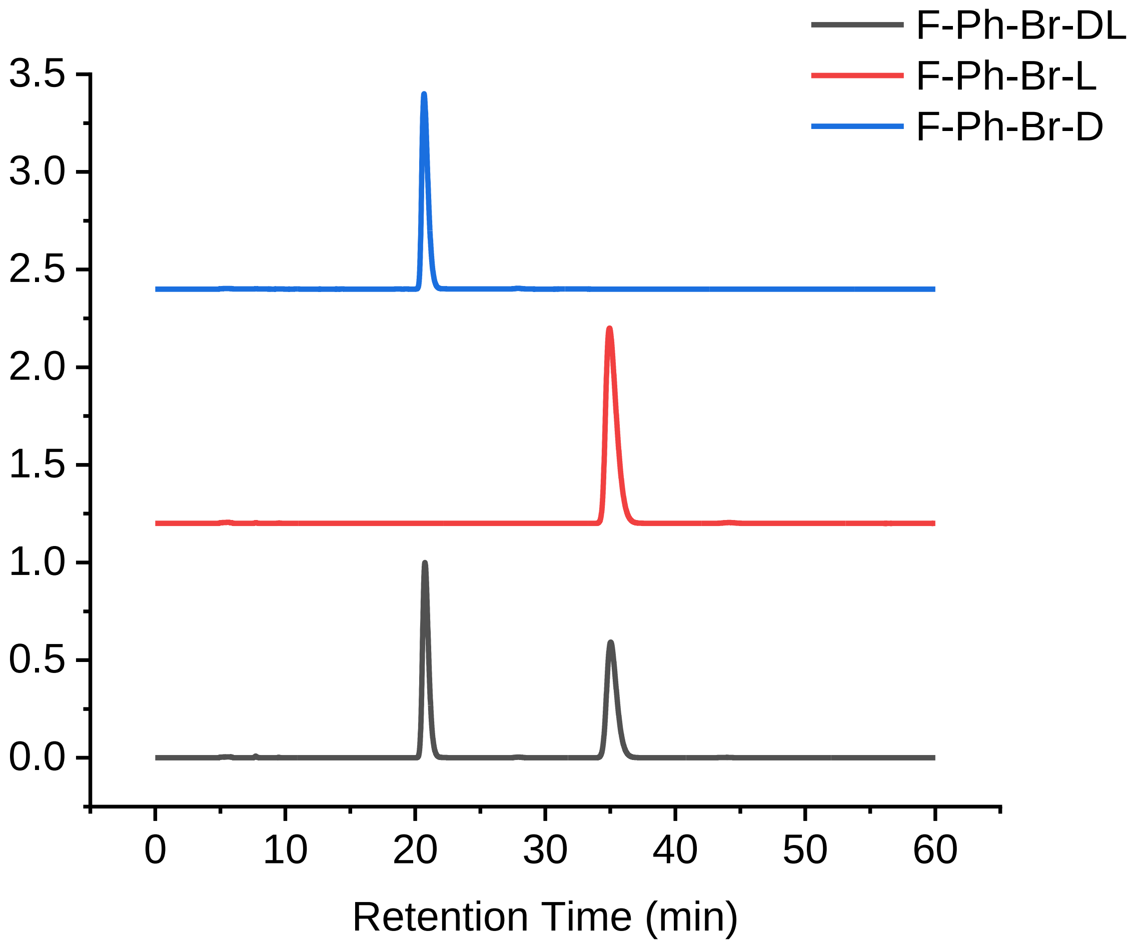

Supplementary Figure 5. Chiral high performance liquid chromatogram (Chiral HPLC) spectrum of the model compounds in ethanol (0.5 mg/ml) with ethanol and n-hexane (25: 75) monitored at the onset absorption of 270 nm.

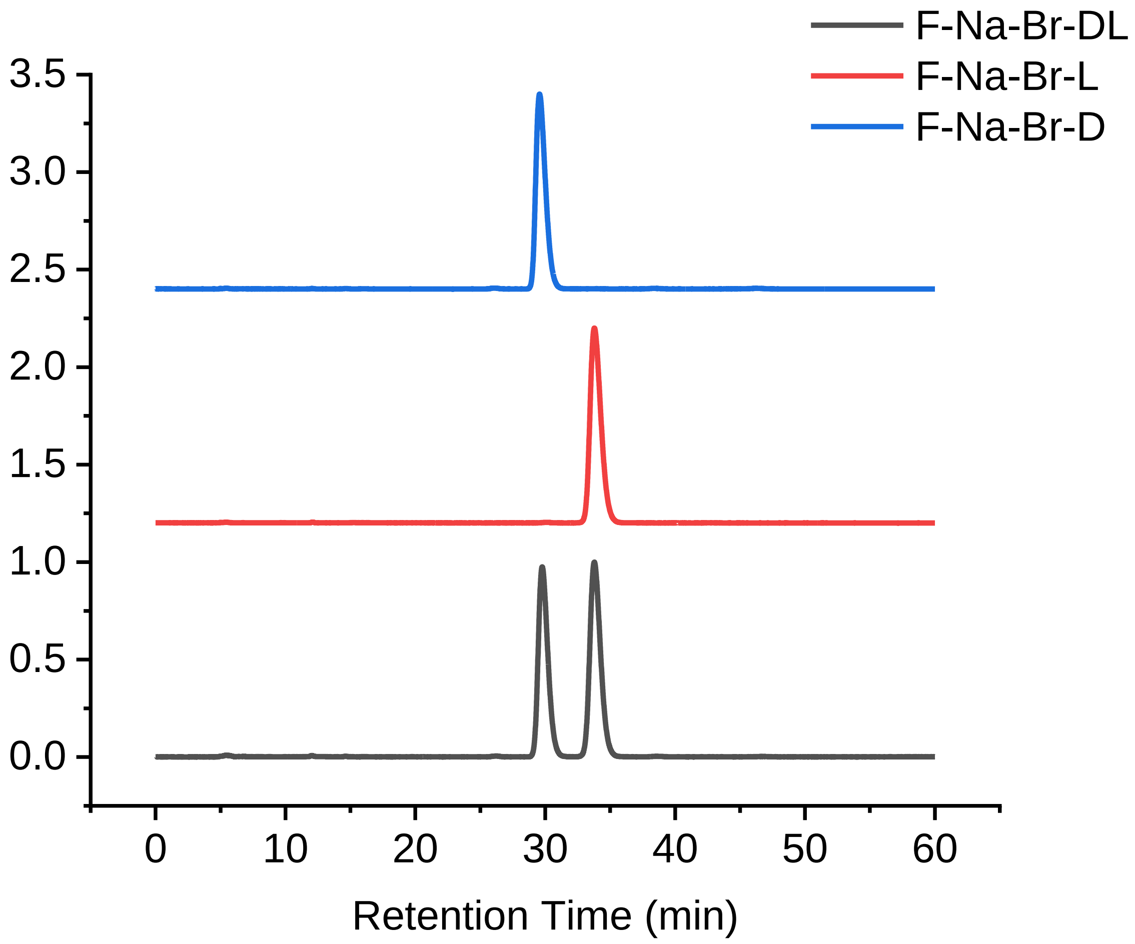

Supplementary Figure 6. Chiral high performance liquid chromatogram (Chiral HPLC) spectrum of the model compounds in ethanol (0.5 mg/ml) with ethanol and n-hexane (33: 67) monitored at the onset absorption of 315 nm.

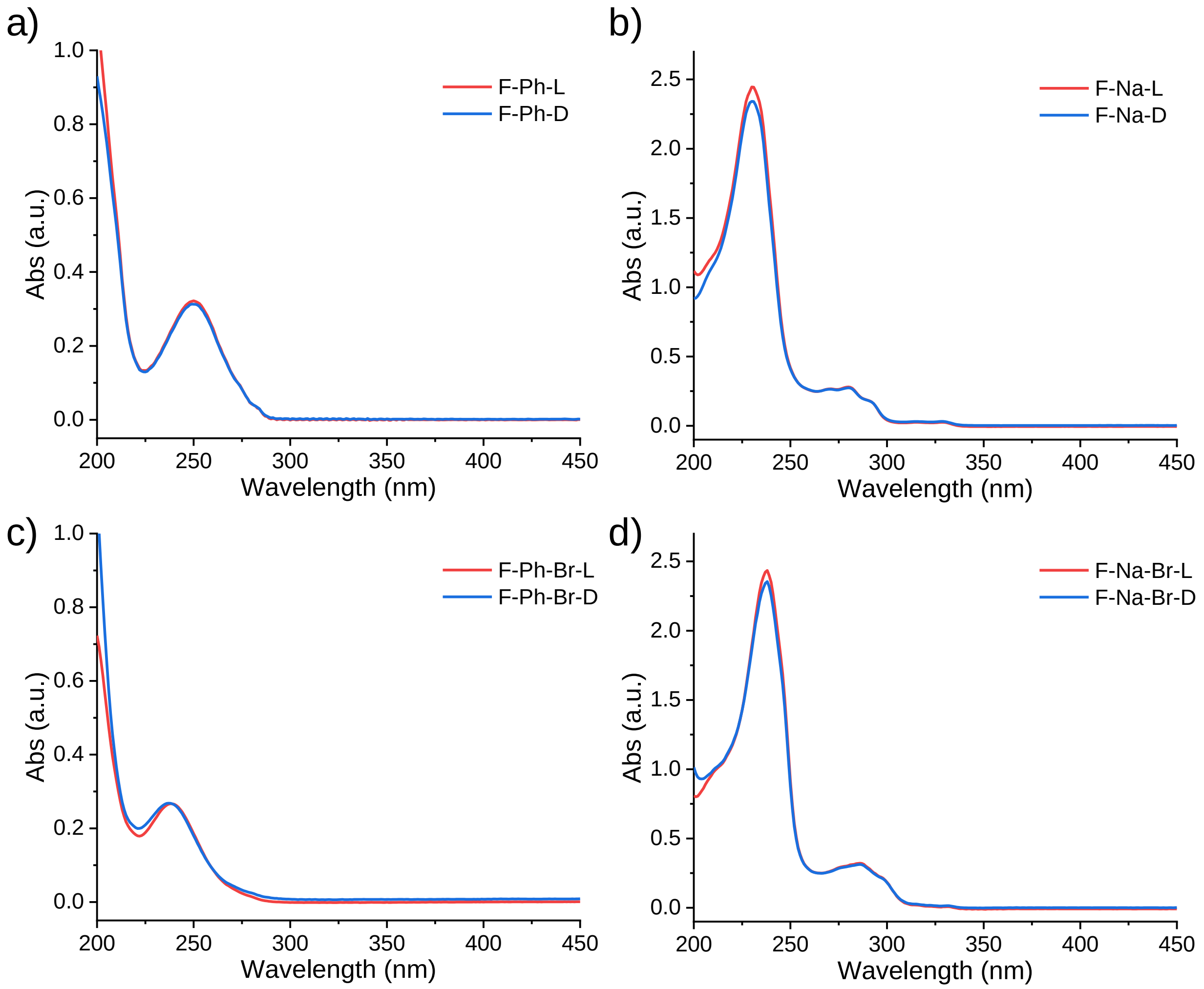

Supplementary Figure 7. UV absorption spectra in acetonitrile (MeCN), 0.02 mM for a) and c), 0.05 mM for b) and 0.04 mM for d) at 298 K.

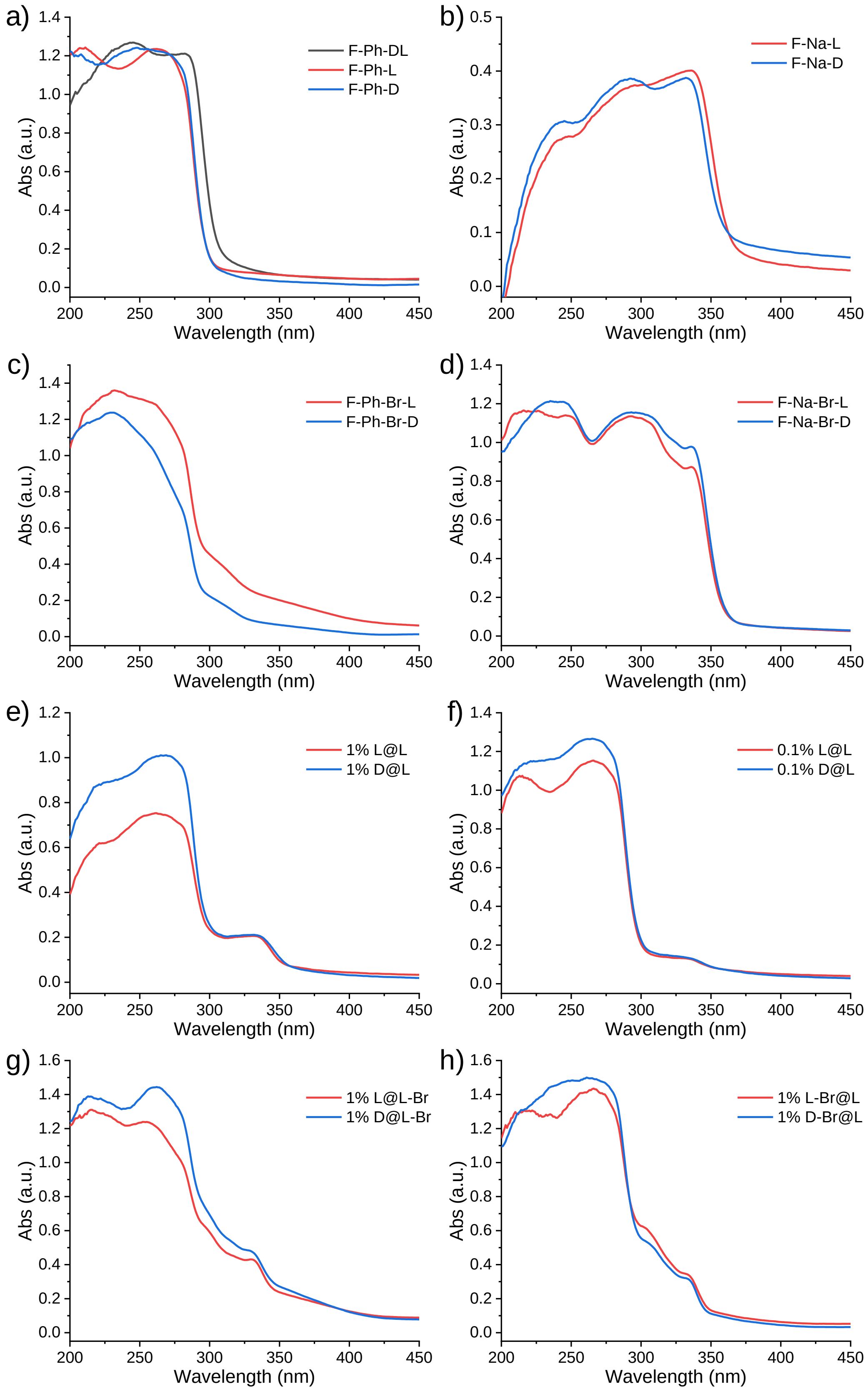

Supplementary Figure 8. a) and c) Solid-state absorption spectra of the host molecules, b) and d) solid-state absorption spectra of the guest molecules, e), f), g) and h) solid-state absorption spectra of the host molecules and doped samples (w/w = 1% and 0.1%).

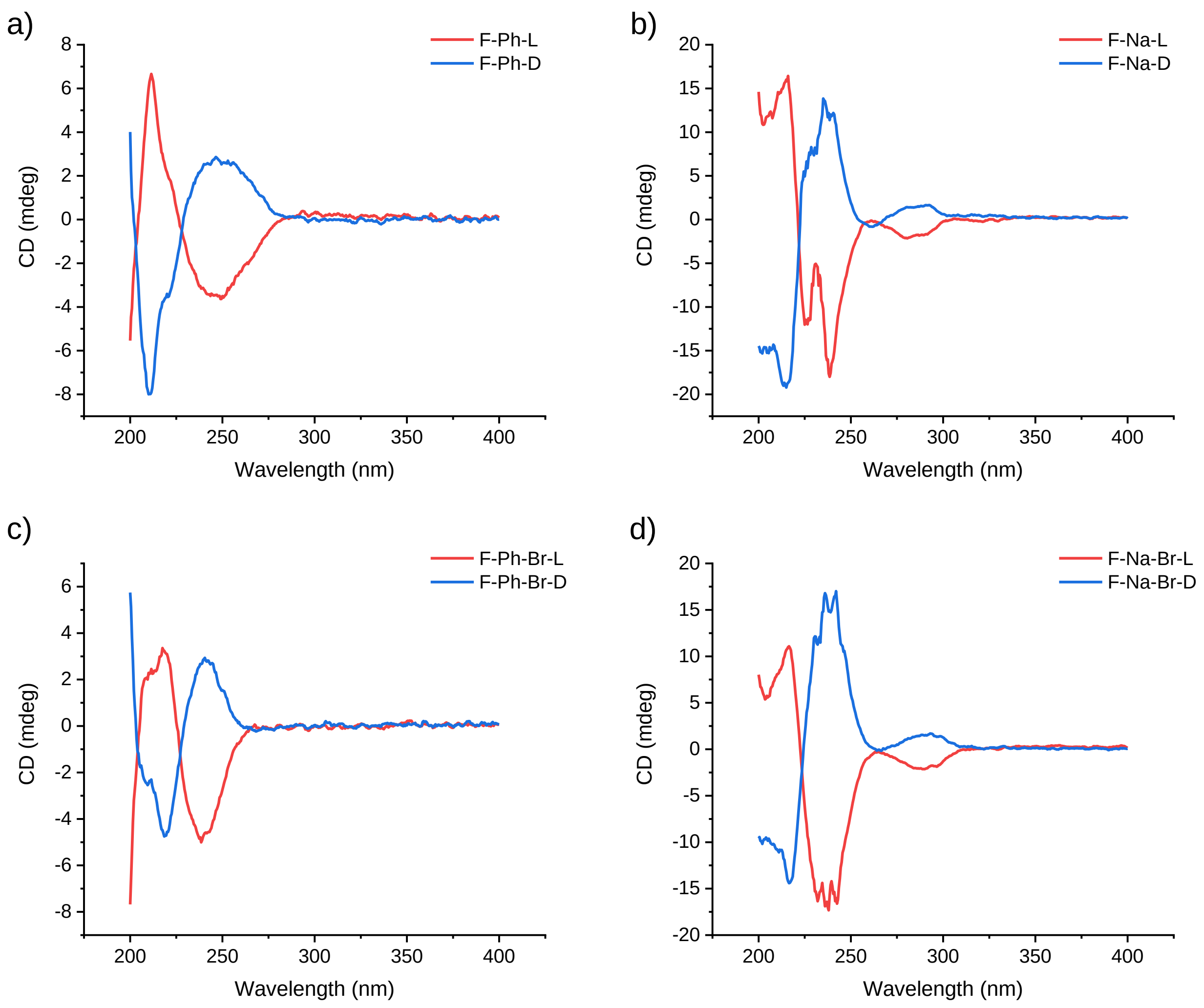

Supplementary Figure 9. Circular dichroism (CD) spectra of dilute solutions of model compounds in acetonitrile (MeCN), 0.04 mM for a) and c), 0.08 mM for b) and 0.06 mM for d) at 298 K.

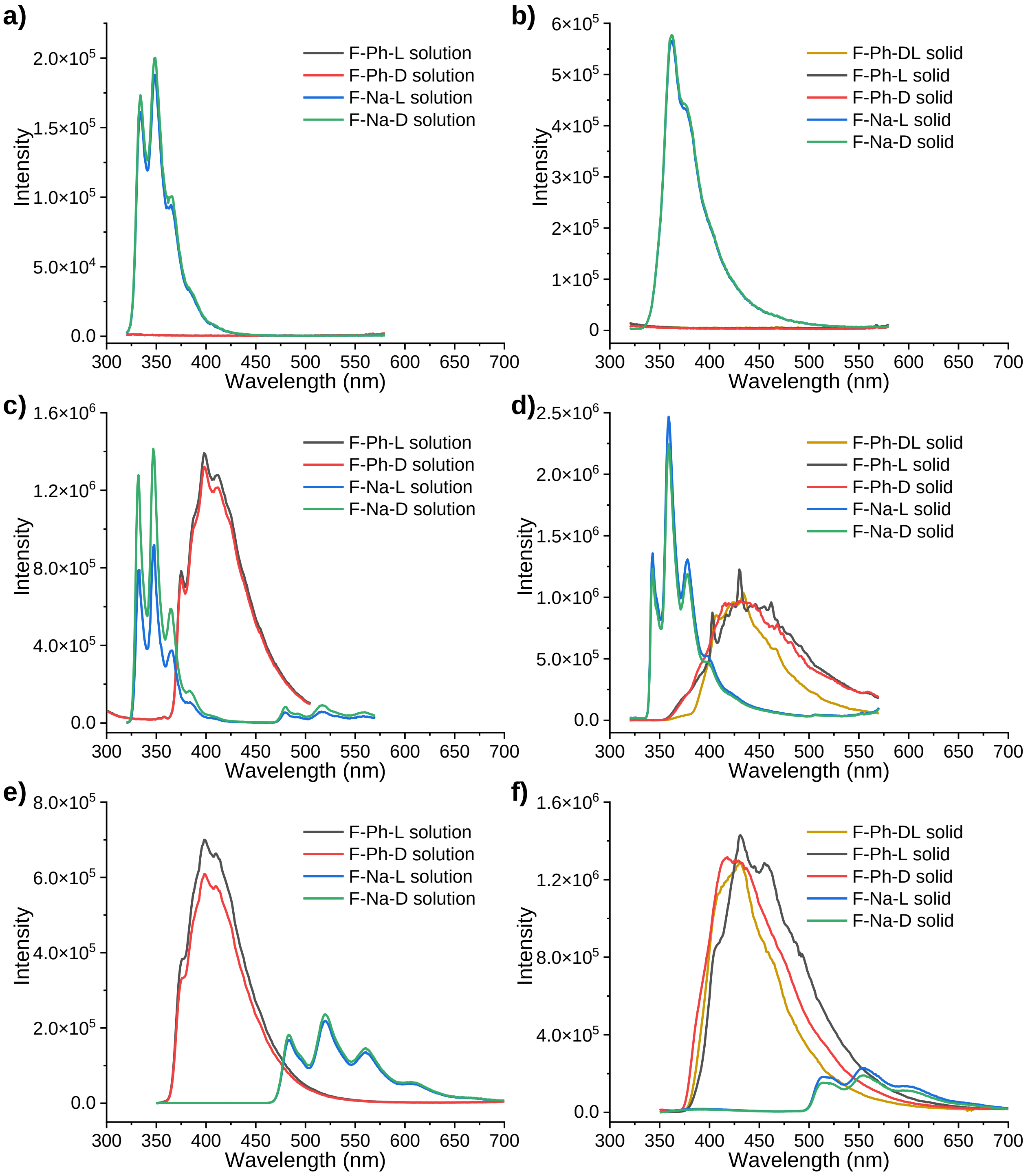

Supplementary Figure 10. a) and b) Photoluminescence (PL) spectra at 298 K, solution: in dimethyl tetrahydrofuran (2-Me-THF) (2 mM) c) and d) PL spectra at 77 K, solution: in 2-Me-THF (0.04 mM) e) and f) Delayed (Δt = 5 ms) emission at 77 K, solution: in 2-Me-THF (0.04 mM)

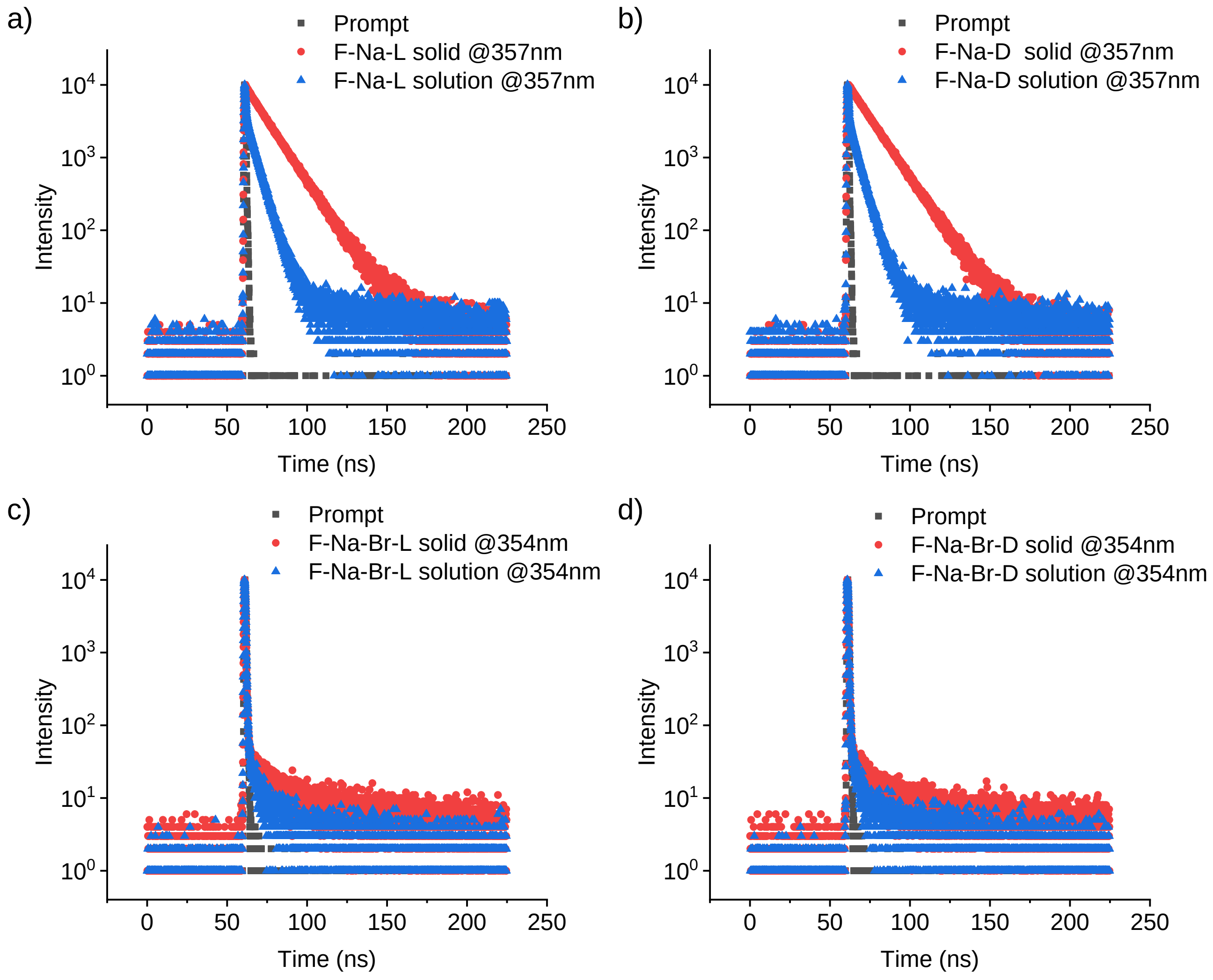

Supplementary Figure 11. Emission decay curves of **F-Na-L** a), **F-Na-D** b), **F-Na-Br-L** c), and **F-Na-Br-D** d) at 298 K (excited with nanoLED-280). Solution: in 2-Me-THF (2 mM).

Supplementary Table 1. Photoluminescence properties of representative molecules in 2-Me-THF.

Molecule	Absorption max[a] (nm)	ε ($L*mol^{-1}*cm^{-1}$)	Emission max (nm)[b]	Lifetime[c] (ns)
**F-Ph-L**	250	16100	/[d]	/
**F-Ph-D**	250	15600	/	/
**F-Na-L**	280	5600	357	5.30 (28.10%)<br>17.7 (1.25%)<br>0.0186 (70.66%)
**F-Na-D**	280	5500	357	5.02 (5.56%)<br>20.4 (0.28%)<br>0.0104 (94.17%)
**F-Ph-Br-L**	238	13000	/	/
**F-Ph-Br-D**	238	13000	/	/
**F-Na-Br-L**	286	8000	354	/
**F-Na-Br-D**	286	7800	354	/

[a] UV absorption spectra in 2-Me-THF (0.02 mM for **F-Ph** and **F-Ph-Br**, 0.05 mM for **F-Na**, 0.04 mM for **F-Na-Br**).
[b] Emission maxima of photoluminescence spectra excited at absorption maxima in 2-Me-THF at 298 K.
[c] Fluorescence lifetime in 2-Me-THF (nanoLED-280).
[d] Too weak to be detected or no emission.

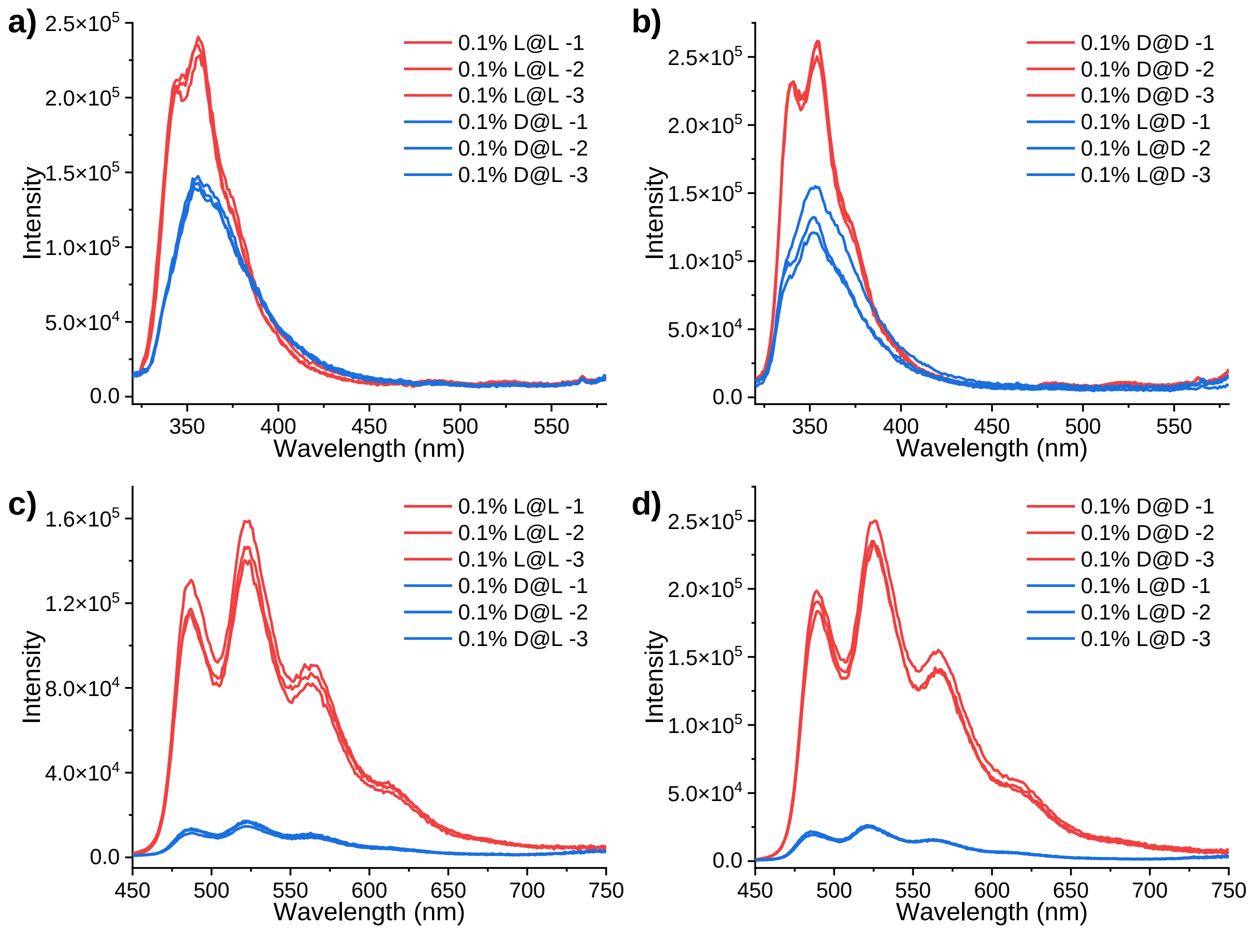

Supplementary Figure 12. Steady-state photoluminescence (PL) spectra of two chiral guests (w/w = 0.1%) in a) **F-Ph-L** and b) **F-Ph-D** at 298 K (λ_{ex} = 298 nm). (The number following the sample name indicates the different individually prepared samples.) Delayed emission (Δt = 5 ms) spectra of two chiral guests (w/w = 0.1%) in c) **F-Ph-L** and d) **F-Ph-D** at 298 K (λ_{ex} = 247 nm).

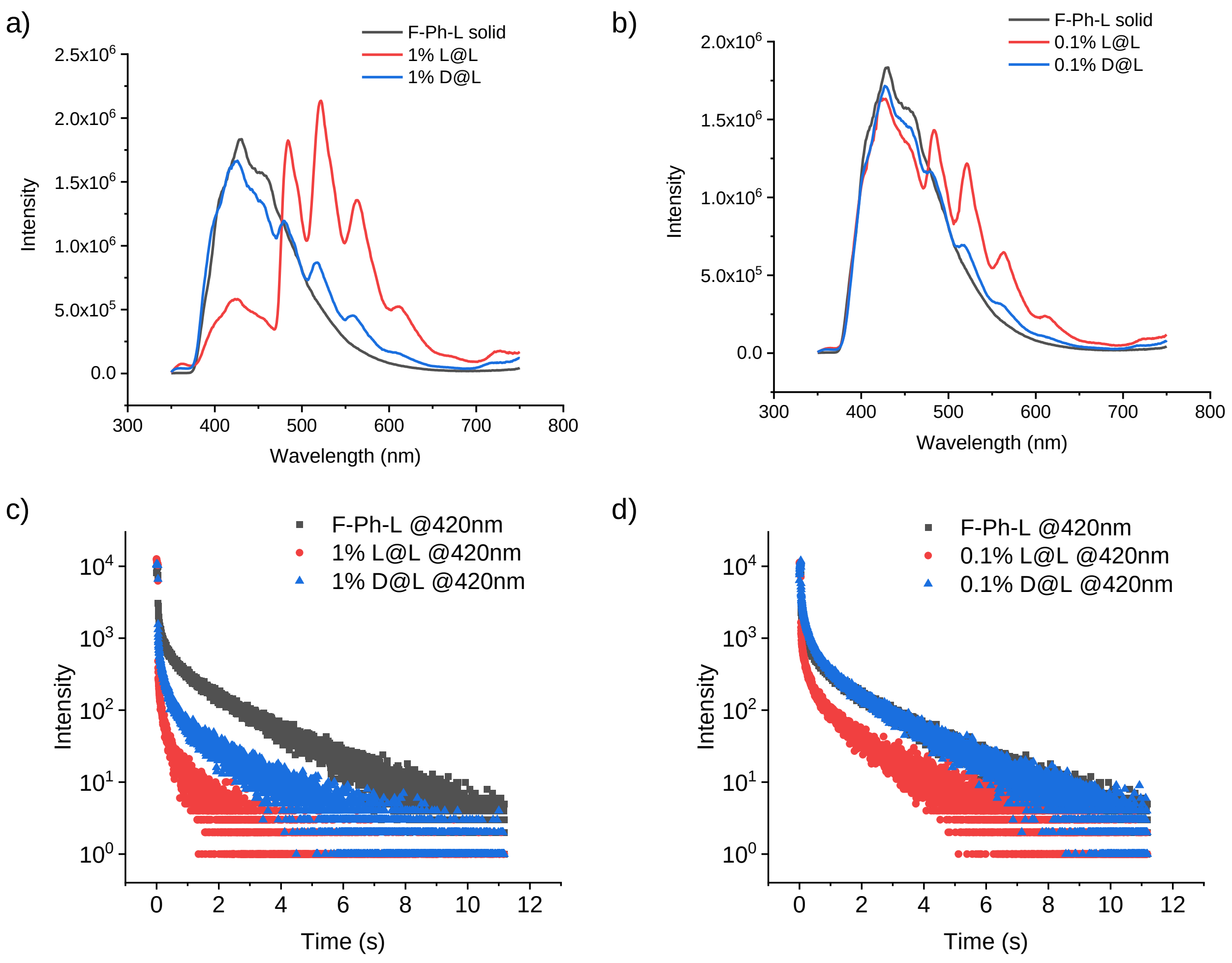

Supplementary Figure 13. a), b) Delayed emission (DE, Δt = 5 ms) spectra of doped samples (w/w = 0.1% or 1%) in air at 77 K (λ_{ex} = 247 nm). c), d) Time-resolved RTP emission for doped samples (w/w = 0.1% or 1%) at 77 K.

Supplementary Table 2 Time-resolved RTP emission for **F-Ph-L** and doped samples (w/w = 0.1% or 1%) at 77 K.

Samples	Emission Wavelength(nm)	Lifetime(s)	Weighted Average Lifetime(s)
**F-Ph-L**	420	0.209 (23.00%)<br>1.77 (73.89%)<br>0.00423 (3.11%)	1.355
**1% L@L**	420	0.130 (36.26%)<br>0.911 (34.23%)<br>0.00203 (29.51%)	0.359
**1% D@L**	420	0.160 (36.30%)<br>1.45 (53.63%)<br>0.00291 (10.07%)	0.834
**0.1% L@L**	420	0.167 (31.60%)<br>1.39 (60.61%)<br>0.00284 (7.79%)	0.894
**0.1% D@L**	420	0.22 (36.75%)<br>1.68 (58.65%)<br>0.00861 (4.60%)	1.064

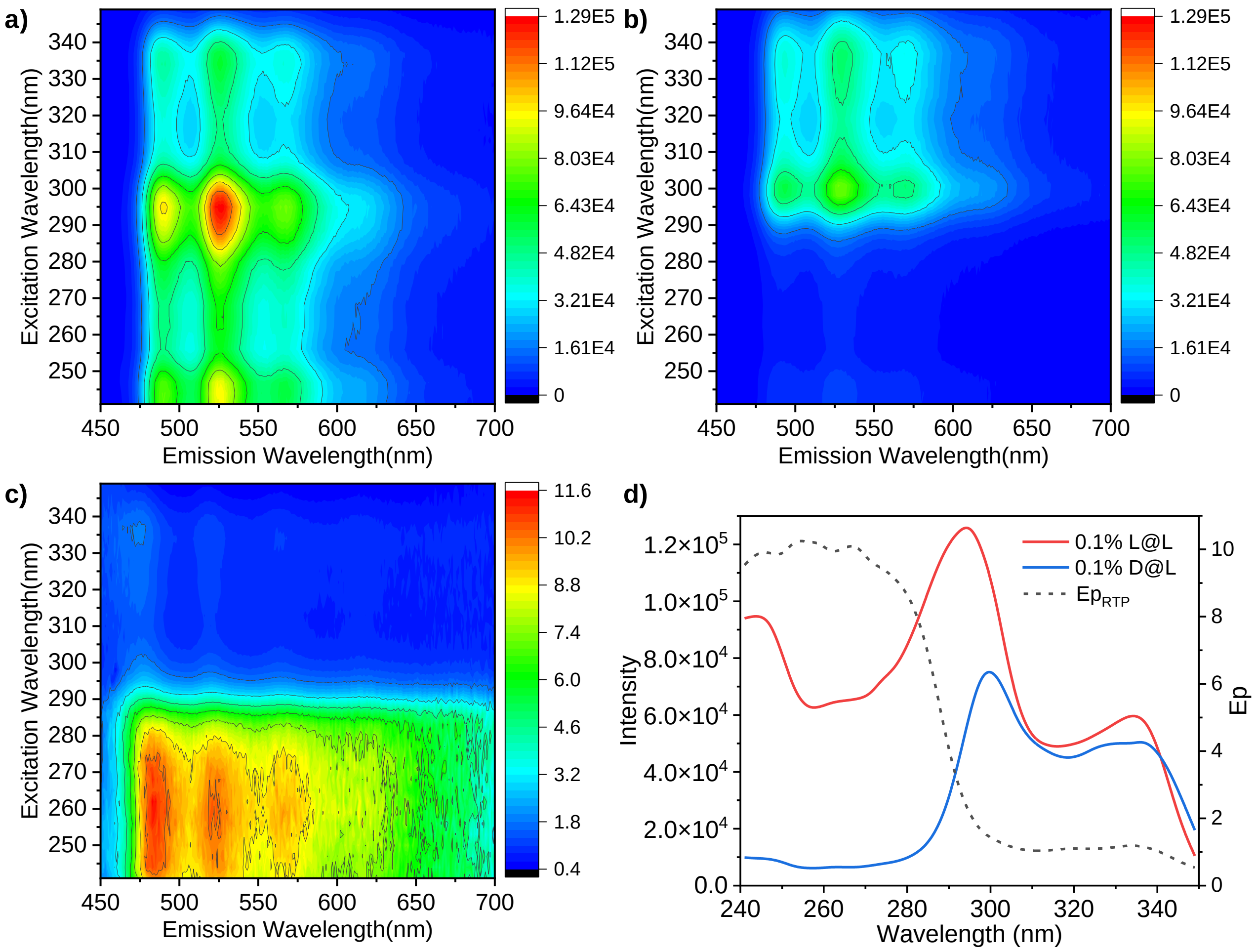

Supplementary Figure 14. The 2D excitation-emission-intensity phosphorescence spectra (Δt = 5 ms) of a) **L@L** and b) **D@L** in air at 298 K. c) The 2D excitation-emission-ep$_{RTP}$ (ep = $I_{L@L}/I_{D@L}$) graphs (Δt = 5 ms) of **L@L** and **D@L** in air at 298 K. (Divide the data in Supplementary Figure 14a by the data in Supplementary Figure 14b) d) Delayed excitation spectra (Δt = 5 ms) of **L@L** and **D@L** (λ_{em} = 526 nm) vs. ep$_{RTP}$ values in air at 298 K. (The guest-to-host ratio is 0.1% for all samples in the solid state)

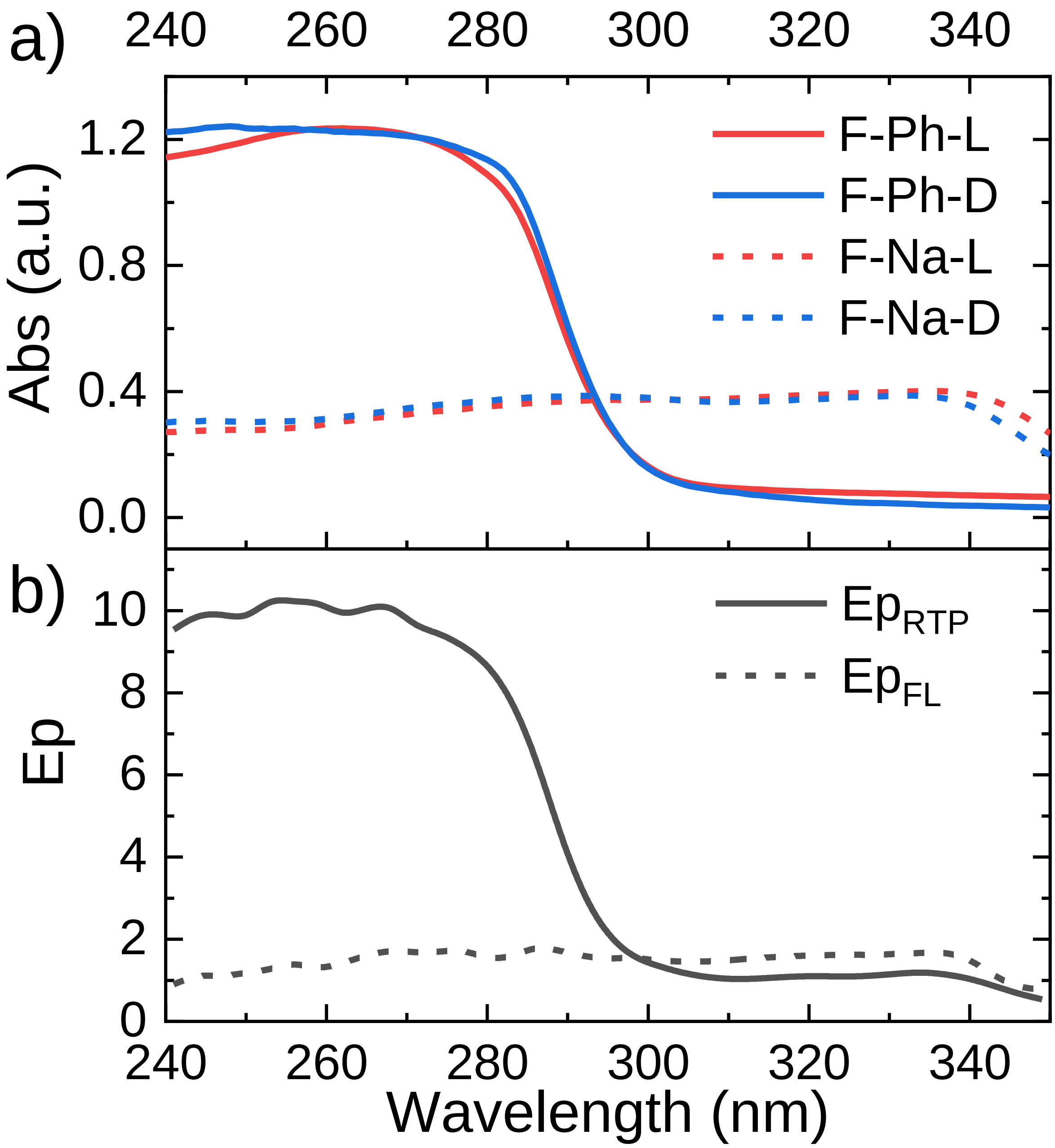

Supplementary Figure 15. a) The solid absorption spectra of the host and guest molecules. b) The wavelength-dependent ep values of the doped samples. (w/w = 0.1%)

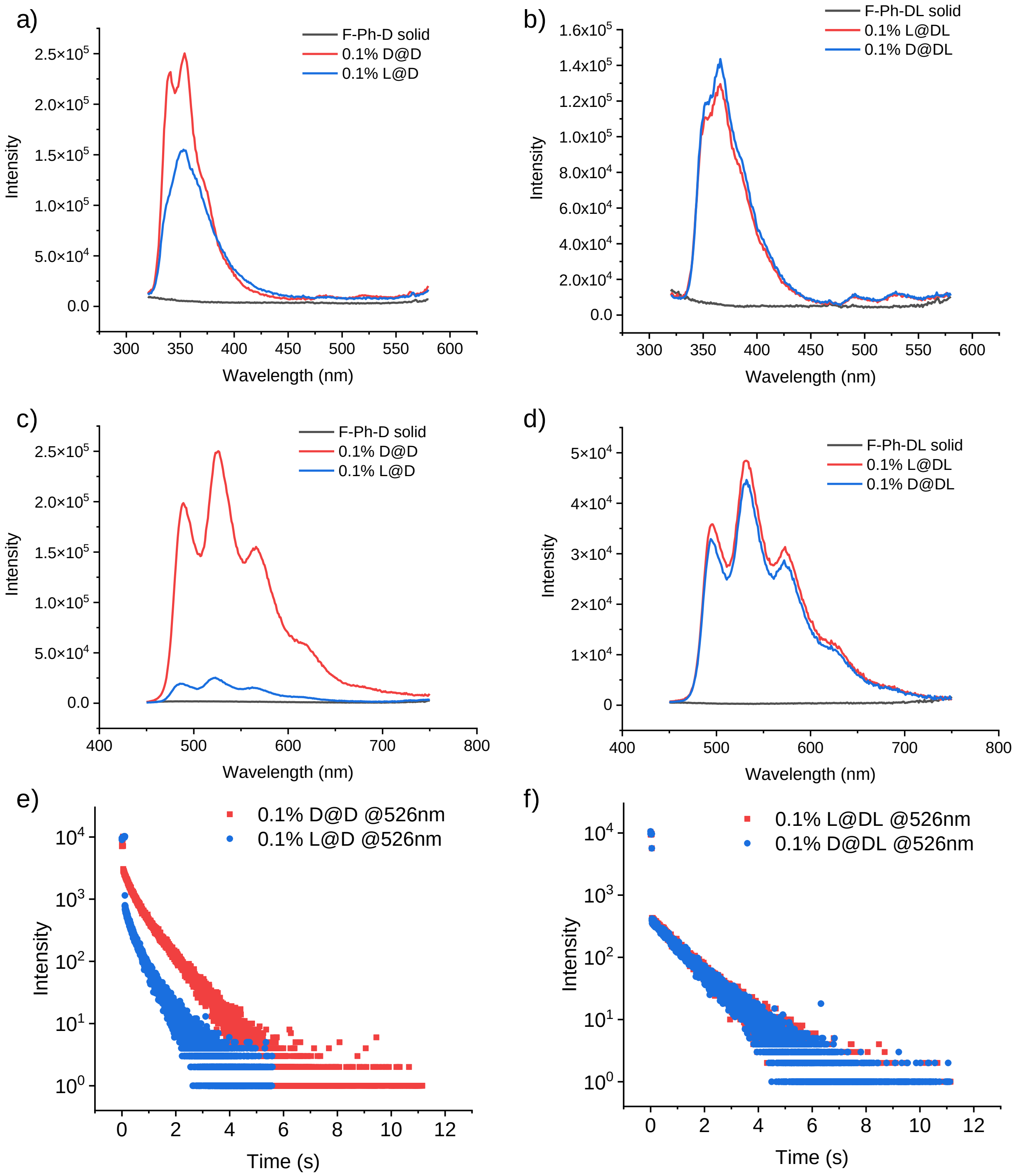

Supplementary Figure 16. a) Steady-state photoluminescence (PL) spectra of **F-Ph-D** and two chiral guests in **F-Ph-D** solid at 298 K (λ_{ex} = 298 nm). b) Steady-state photoluminescence (PL) spectra of **F-Ph-DL** and two chiral guests in **F-Ph-DL** solid at 298 K (λ_{ex} = 298 nm). c) Delayed emission (DE, Δt = 5 ms) spectra of **F-Ph-D** and two chiral guests in **F-Ph-D** solid at 298 K (λ_{ex} = 247 nm). d) Delayed emission (DE, Δt = 5 ms) spectra of **F-Ph-DL** and two chiral guests in **F-Ph-DL** solid at 298 K (λ_{ex} = 247 nm). e) Time-resolved RTP emission for two guests in **F-Ph-D** solid. f) Time-resolved RTP emission for two guests in **F-Ph-DL** solid. (The guest-to-host ratio is 0.1% for all samples in the solid state)

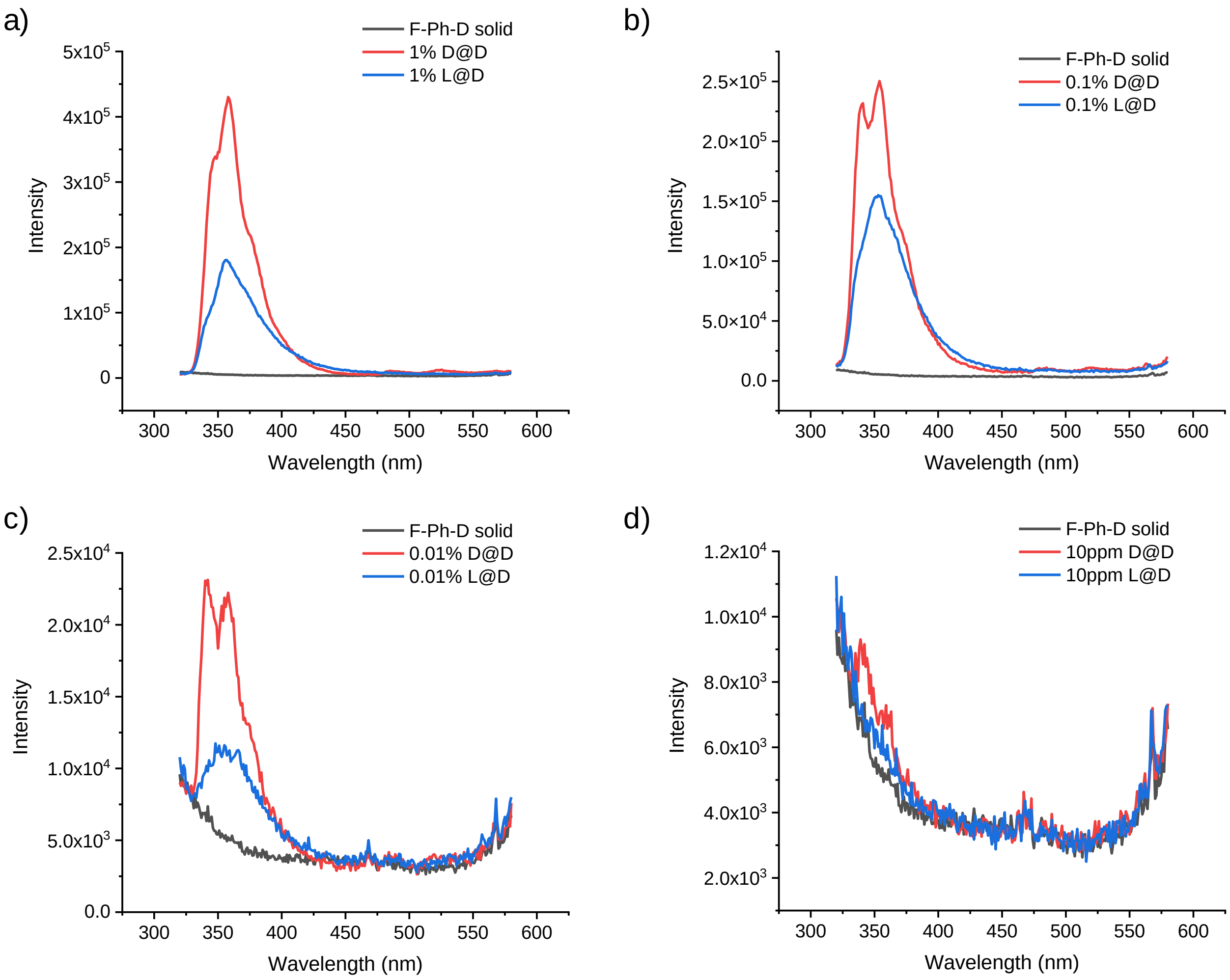

Supplementary Figure 17. Steady-state spectra of L and D guests in **F-Ph-D** solid matrix in air at 298 K (λ_{ex} = 298 nm), w/w = a) 1%, b) 0.1%, c) 0.01%, d) 10ppm.

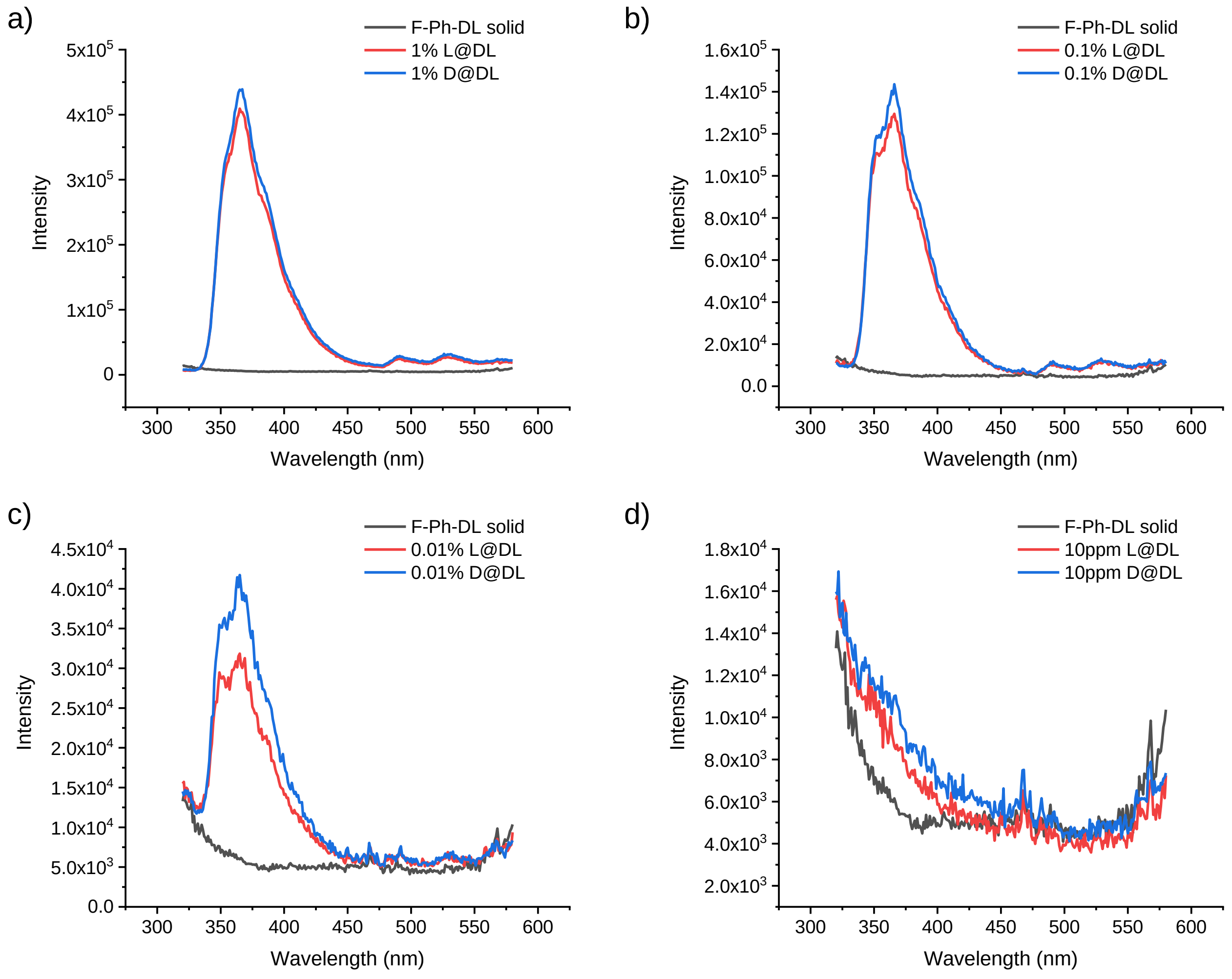

Supplementary Figure 18. Steady-state spectra of L and D guests in **F-Ph-DL** solid matrix in air at 298 K (λ_{ex} = 298 nm), w/w = a) 1%, b) 0.1%, c) 0.01%, d) 10ppm.

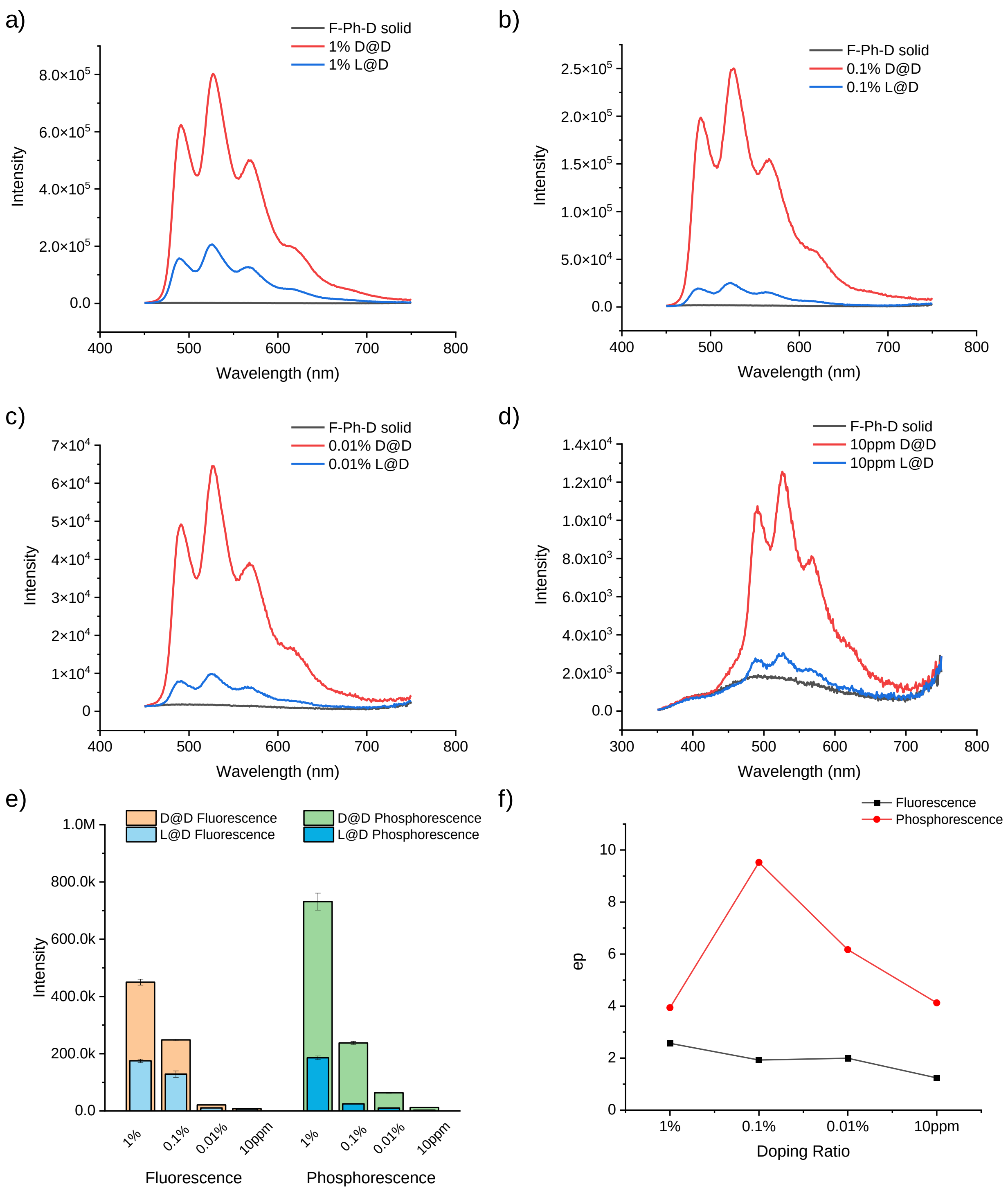

Supplementary Figure 19. Delayed emission (DE, Δt = 5 ms) spectra of D and L guests doped in **F-Ph-D** solid in air at 298 K (λ_{ex} = 247 nm), w/w = a) 1%, b) 0.1%, c) 0.01%, d) 10ppm. e) and f) The ep value (intensity of steady-state photoluminescence at 357 nm and delayed emission at 526 nm) vs. dope ratios of guest in **F-Ph-D**. (w/w = 10 ppm - 1%, values are means ± s.e.m.; n = 3 or 4)

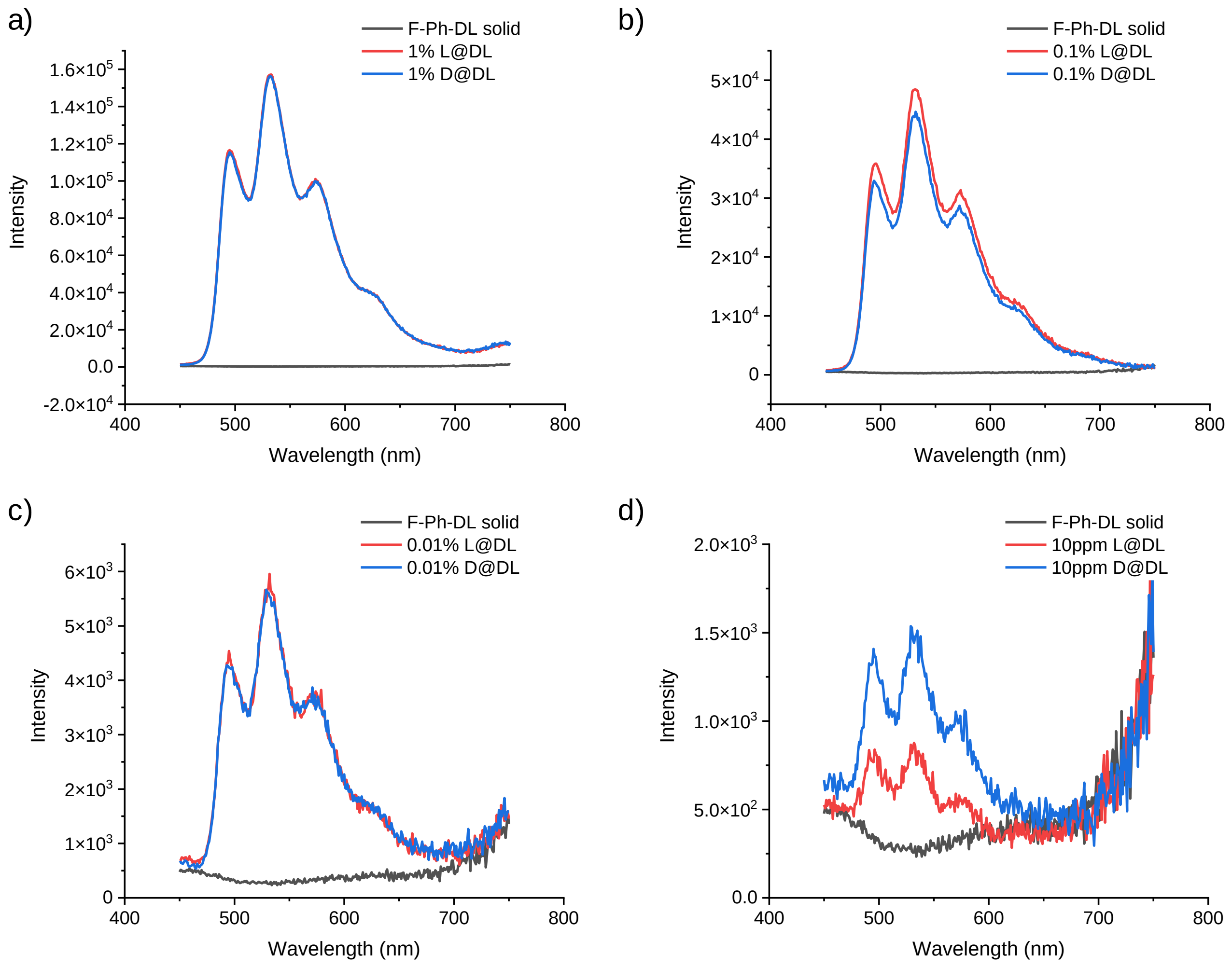

Supplementary Figure 20. Delayed emission (DE, Δt = 5 ms) spectra of L and D guests doped in **F-Ph-DL** solid in air at 298 K (λ_{ex} = 247 nm), w/w = a) 1%, b) 0.1%, c) 0.01%, d) 10ppm.

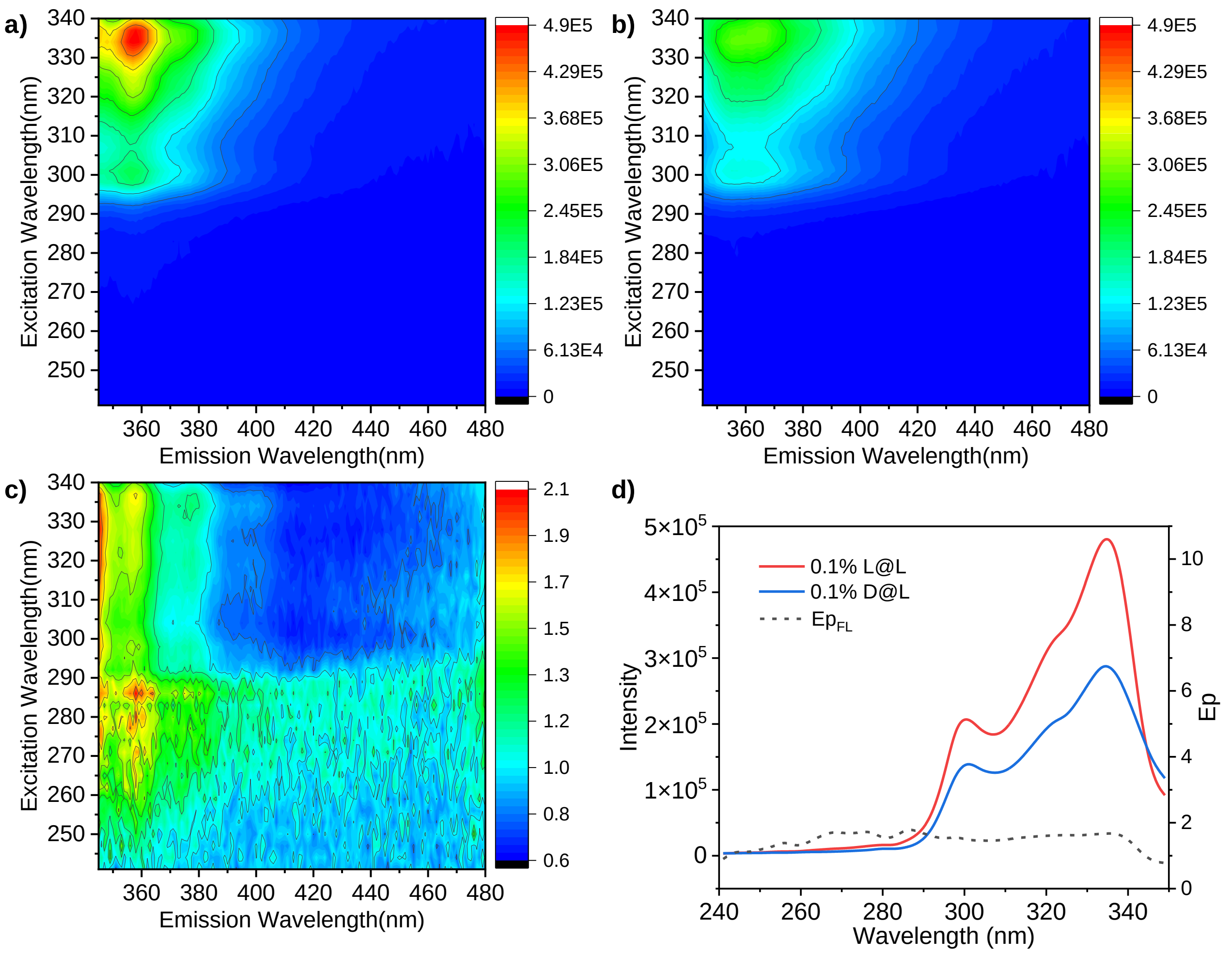

Supplementary Figure 21. The 2D excitation-emission-intensity fluorescence spectra of a) **L@L** and b) **D@L** in air at 298 K. c) The 2D excitation-emission-ep$_{FL}$ graphs of **L@L** and **D@L** in air at 298 K. (Divide the data in Supplementary Figure 21a by the data in Supplementary Figure 21b) d) Steady-state excitation spectra of **L@L** and **D@L** (λ_{em} = 357 nm) vs. ep$_{FL}$ values in air at 298 K. (The guest-to-host ratio is 0.1% for all samples in the solid state)

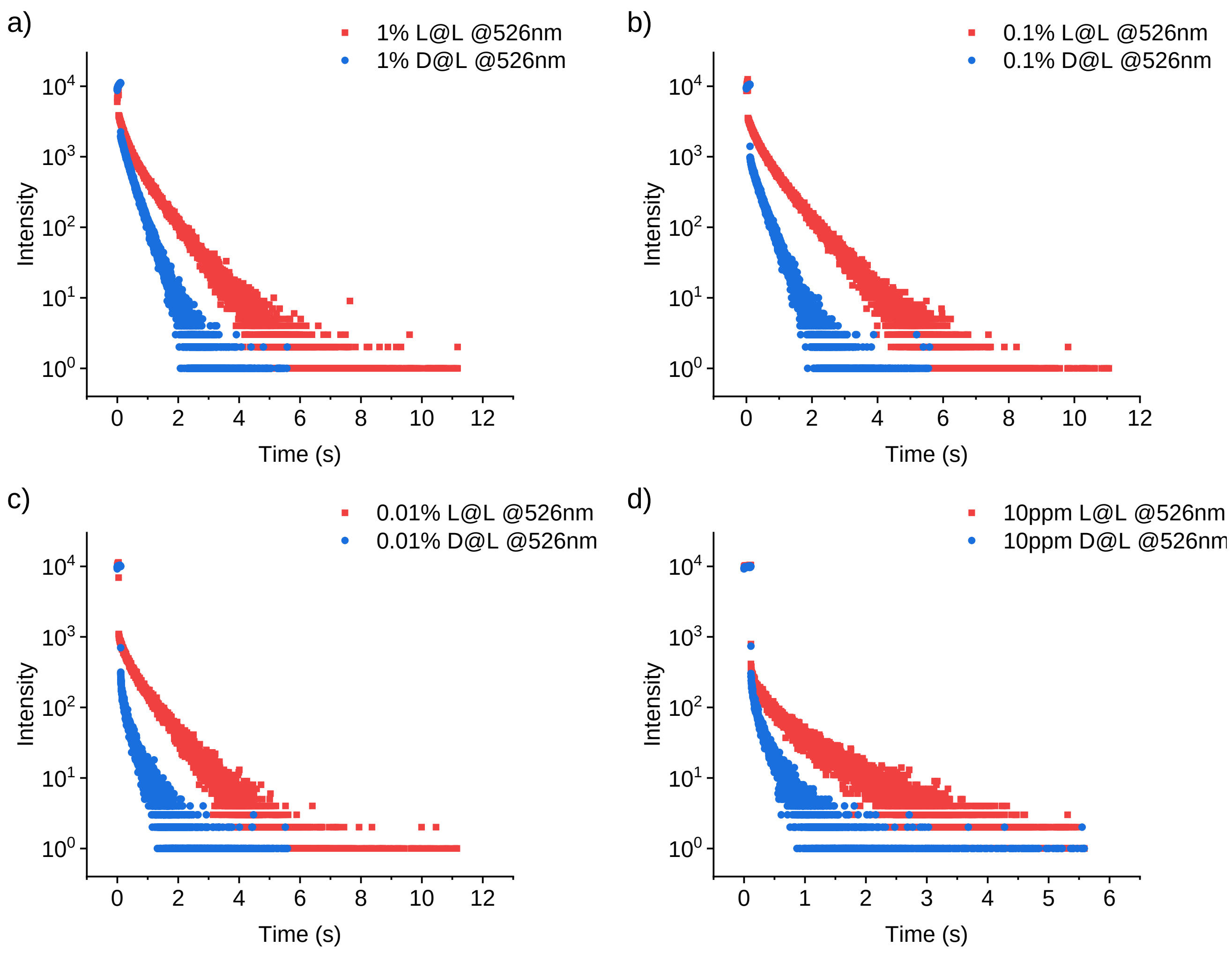

Supplementary Figure 22. Time-resolved RTP emission spectra for two guests doped in **F-Ph-L** solid, w/w = a) 1%, b) 0.1%, c) 0.01%, d) 10ppm.

Supplementary Table 3. Time-resolved RTP emission for two guests doped in **F-Ph-L** (w/w = 10 ppm - 1%) at 298 K.

Samples	Emission max(nm)	Lifetime(s)	Weighted Average Lifetime(s)
**1% L@L**	526	0.216 (29.64%)<br>0.746 (69.26%)<br>0.00277 (1.10%)	0.581
**1% D@L**	526	0.157 (16.13%)<br>0.356 (83.26%)<br>0.000399 (0.62%)	0.322
**0.1% L@L**	526	0.249 (25.79%)<br>0.787 (72.80%)<br>0.00258 (1.41%)	0.637
**0.1% D@L**	526	0.328 (98.31%)<br>0.000483 (1.69%)	0.322
**0.01% L@L**	526	0.218 (19.49%)<br>0.781 (76.03%)<br>0.00217 (4.47%)	0.637
**0.01% D@L**	526	0.287 (90.82%)<br>0.000478 (9.18%)	0.261
**10ppm L@L**	526	0.125 (17.86%)<br>0.680 (78.33%)<br>0.000455 (3.81%)	0.555
**10ppm D@L**	526	0.172 (85.35%)<br>0.000493 (14.65%)	0.147

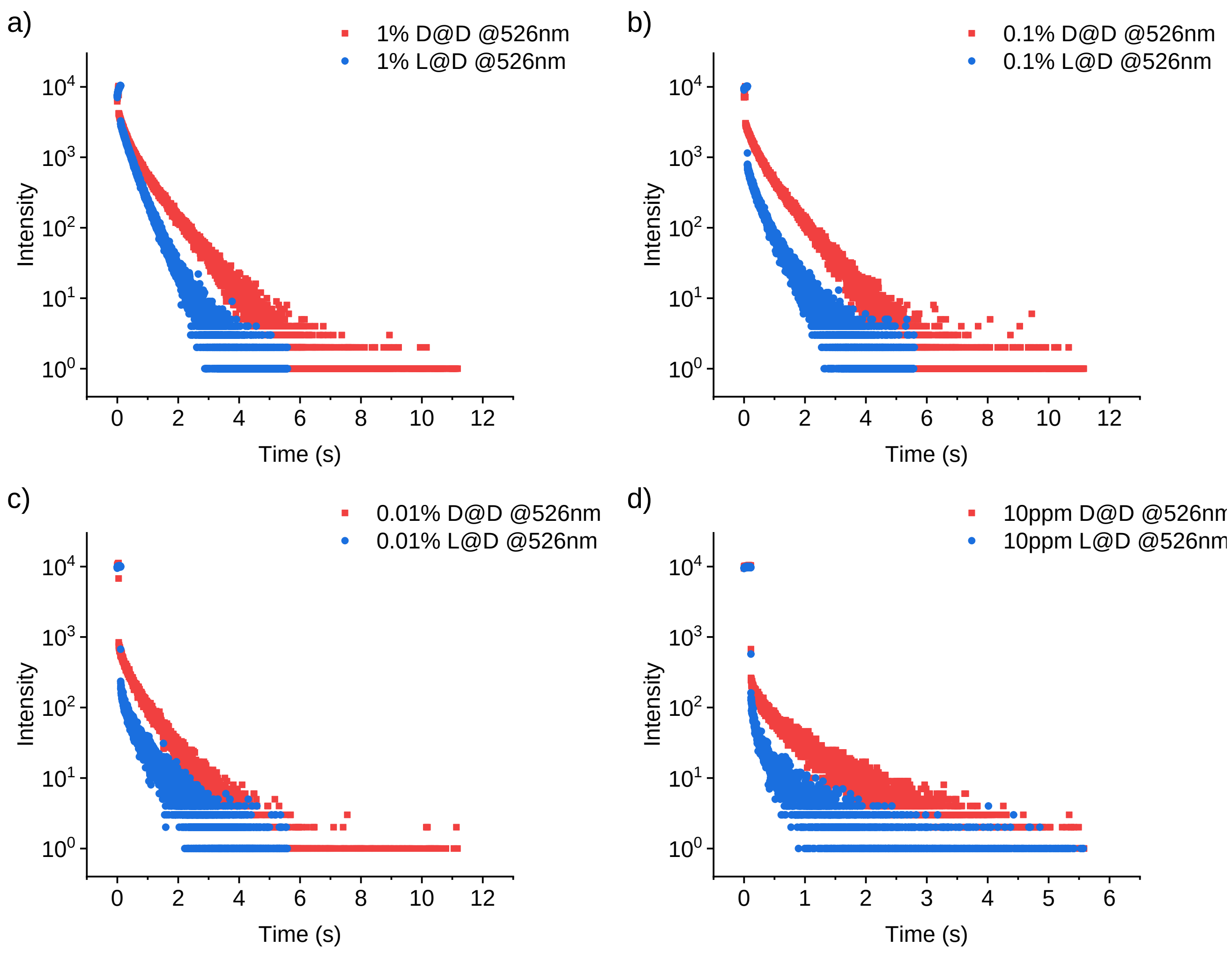

Supplementary Figure 23. Time-resolved RTP emission spectra for two guests doped in **F-Ph-D** solid, w/w = a) 1%, b) 0.1%, c) 0.01%, d) 10ppm.

Supplementary Table 4. Time-resolved RTP emission for two guests doped in **F-Ph-D** (w/w = 10 ppm - 1%) at 298 K.

Samples	Emission max(nm)	Lifetime(s)	Weighted Average Lifetime(s)
**1% D@D**	526	0.235 (29.82%)<br>0.785 (69.22%)<br>0.00284 (0.96%)	0.613
**1% L@D**	526	0.248 (43.88%)<br>0.478 (55.8%)<br>0.000429 (0.31%)	0.376
**0.1% D@D**	526	0.260 (26.62%)<br>0.804 (72.05%)<br>0.00264 (1.33%)	0.649
**0.1% L@D**	526	0.217 (33.87%)<br>0.590 (64.56%)<br>0.000445 (1.57%)	0.454
**0.01% D@D**	526	0.214 (21.01%)<br>0.738 (72.70%)<br>0.00205 (6.28%)	0.581
**0.01% L@D**	526	0.120 (13.41%)<br>0.621 (80.68%)<br>0.000458 (5.91%)	0.516
**10ppm D@D**	526	0.131 (16.31%)<br>0.656 (78.90%)<br>0.000443 (4.79%)	0.539
**10ppm L@D**	526	0.0550 (22.14%)<br>0.350 (58.52%)<br>0.000449 (19.34%)	0.217

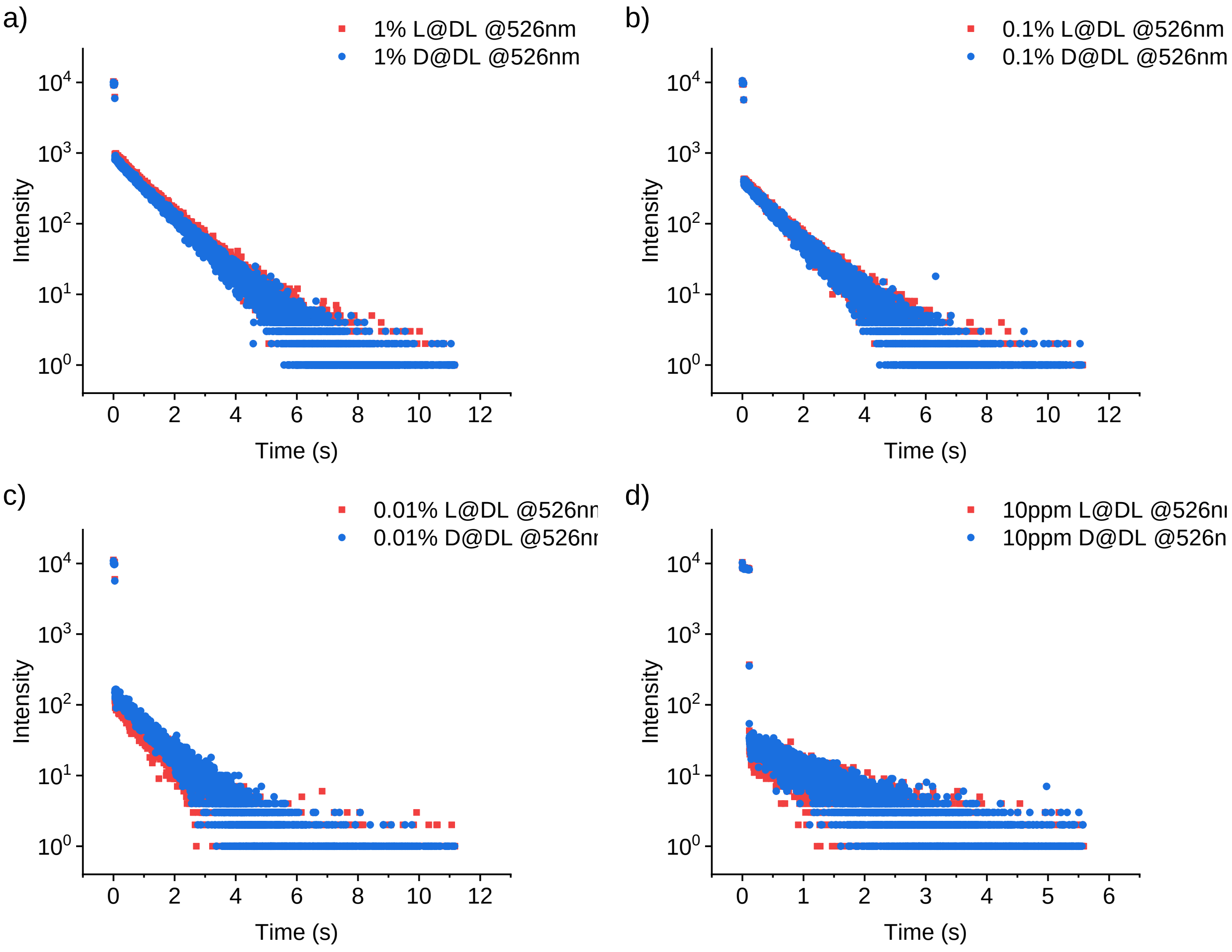

Supplementary Figure 24. Time-resolved RTP emission spectra for two guests doped in **F-Ph-DL** solid, w/w = a) 1%, b) 0.1%, c) 0.01%, d) 10ppm.

Supplementary Table 5. Time-resolved RTP emission for two guests doped in **F-Ph-DL** (w/w = 10 ppm - 1%) at 298 K.

Samples	Emission max(nm)	Lifetime(s)	Weighted Average Lifetime(s)
**1% L@DL**	526	1.05 (97.93%)<br>0.00217 (2.07%)	1.032
**1% D@DL**	526	1.03 (97.76%)<br>0.00206 (2.24%)	1.011
**0.1% L@DL**	526	1.03 (95.74%)<br>0.00184 (4.26%)	0.986
**0.1% D@DL**	526	1.01 (95.57%)<br>0.00180 (4.43%)	0.968
**0.01% L@DL**	526	0.969 (86.74%)<br>0.00140 (13.26%)	0.841
**0.01% D@DL**	526	0.979 (89.93%)<br>0.00150 (10.07%)	0.881
**10ppm L@DL**	526	0.798 (83.90%)<br>0.0405 (0.33%)<br>0.000432 (15.77%)	0.670
**10ppm D@DL**	526	0.849 (87.16%)<br>0.00551 (0.34%)<br>0.000419 (12.51%)	0.740

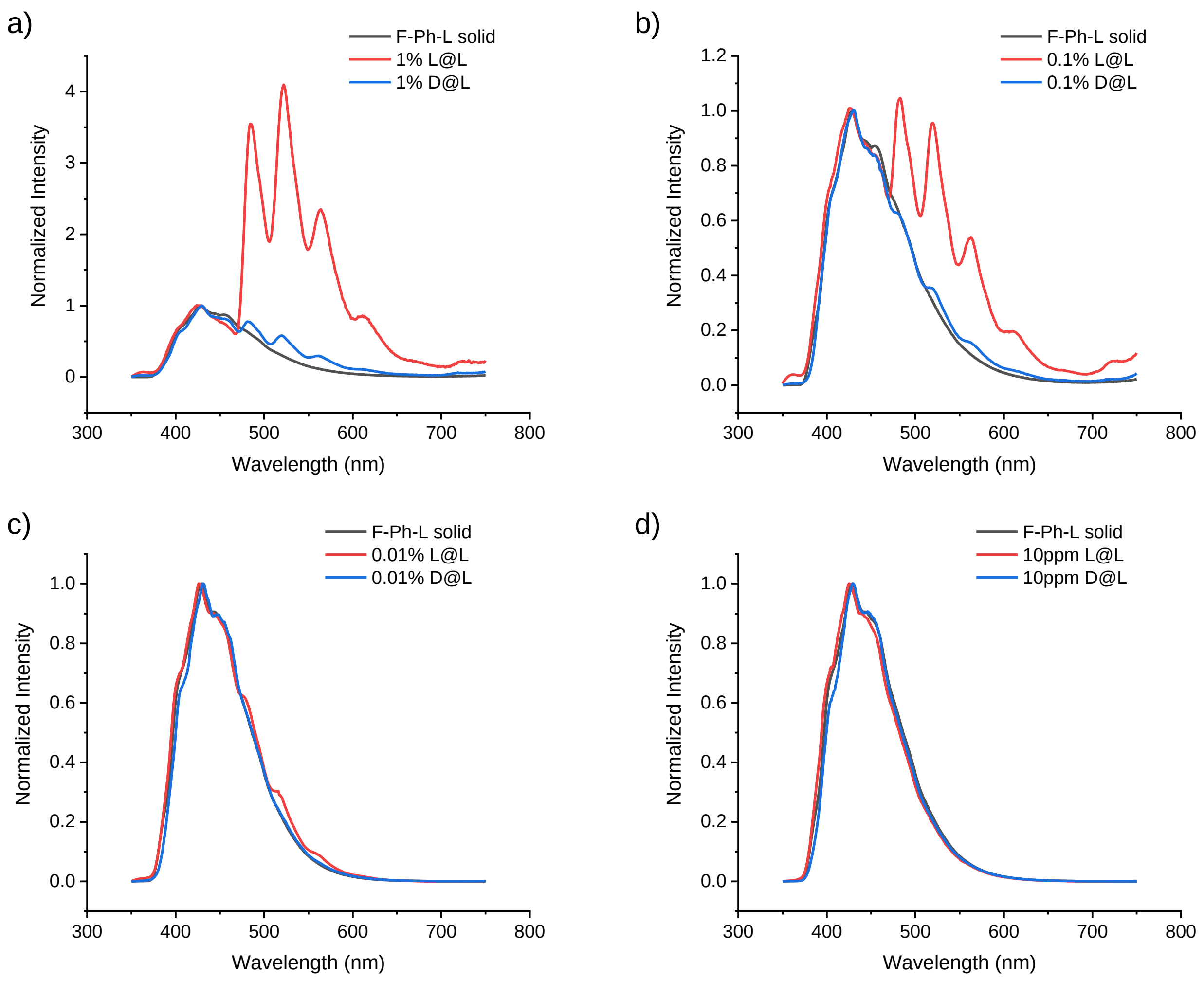

Supplementary Figure 25. Delayed emission (DE, Δt = 5 ms) spectra of two guests doped in **F-Ph-L** solid in air at 77 K (λ_{ex} = 247 nm), w/w = a) 1%, b) 0.1%, c) 0.01%, d) 10ppm.

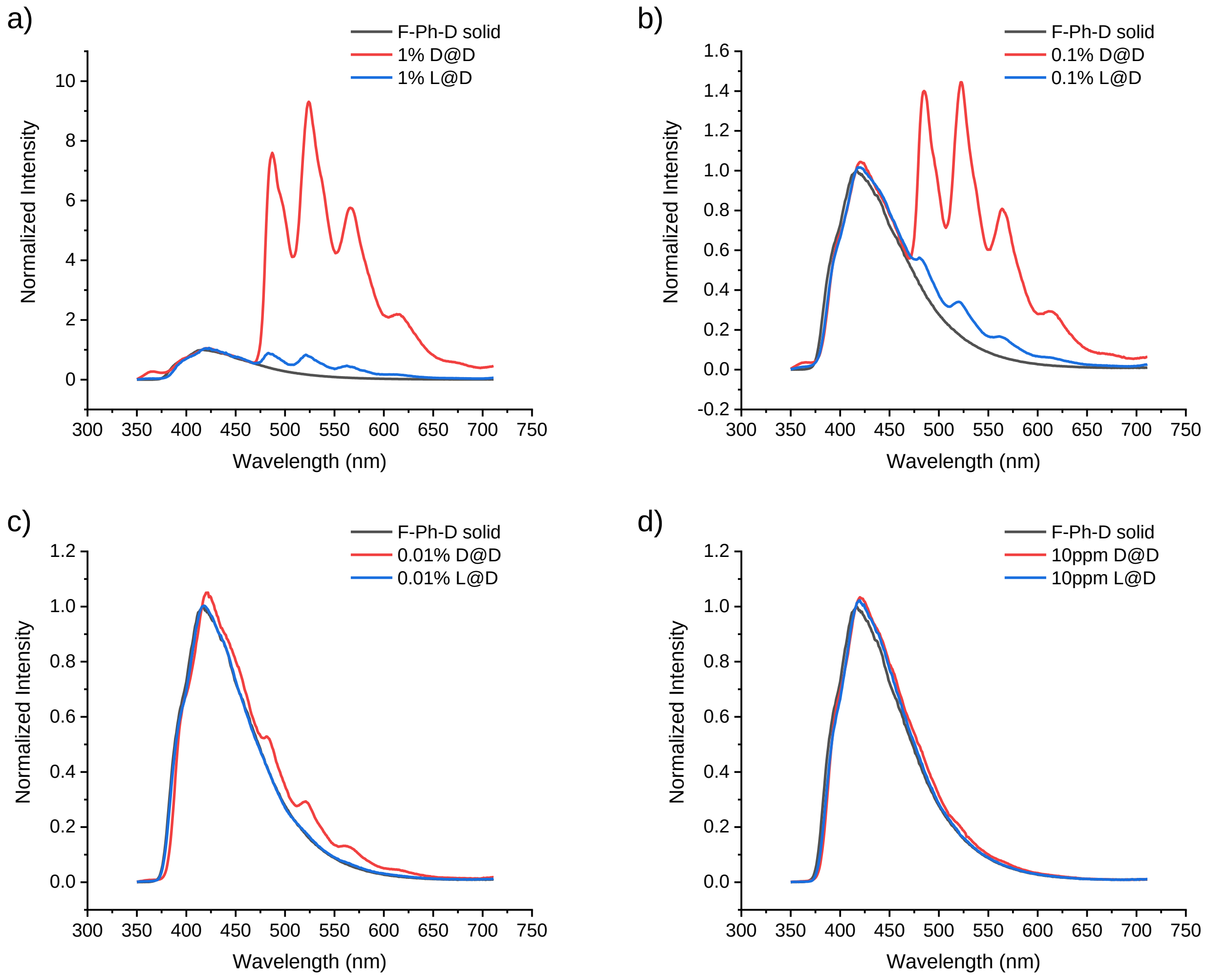

Supplementary Figure 26. Delayed emission (DE, Δt = 5 ms) spectra of two guests doped in **F-Ph-D** solid in air at 77 K (λ_{ex} = 247 nm), w/w = a) 1%, b) 0.1%, c) 0.01%, d) 10ppm.

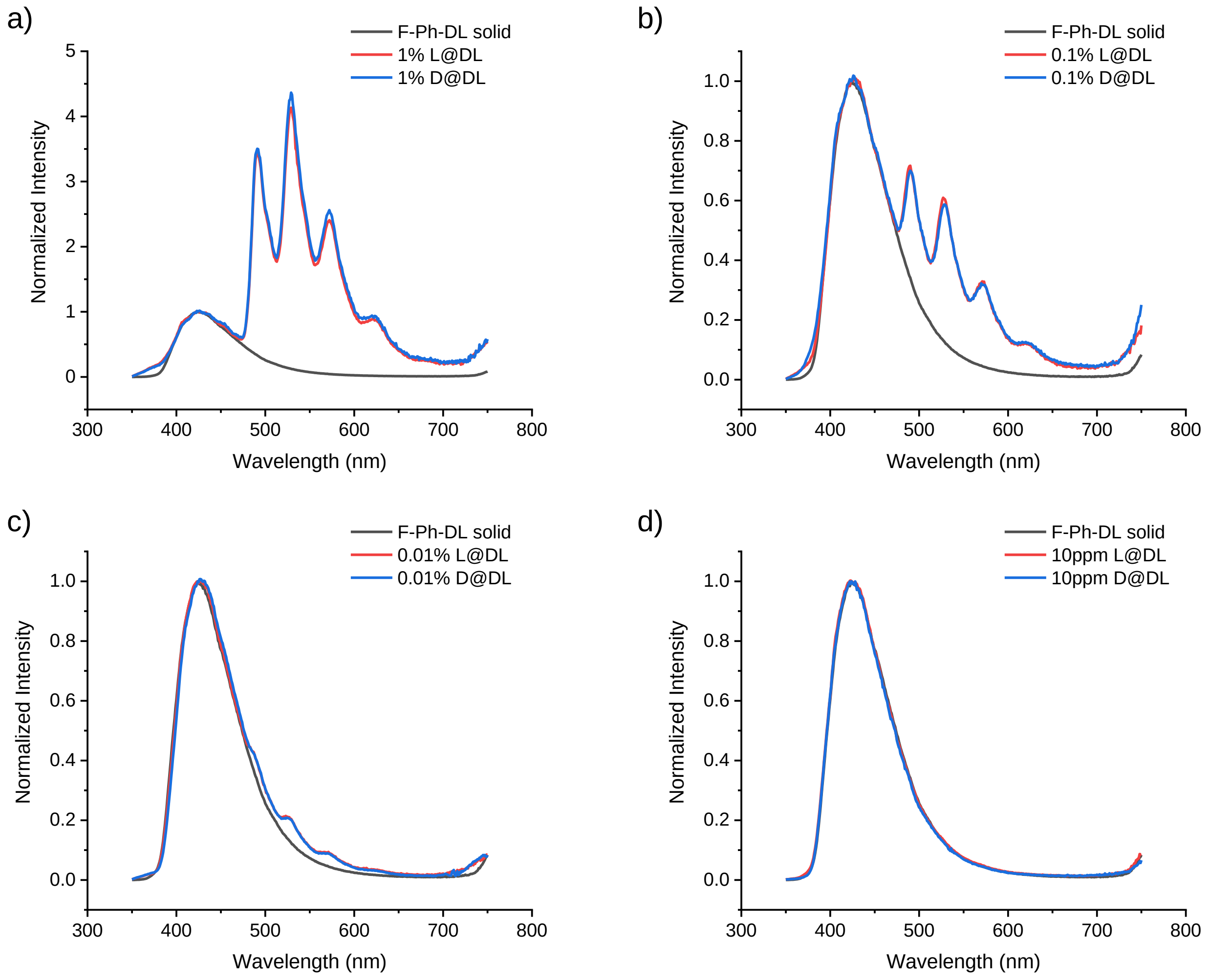

Supplementary Figure 27. Delayed emission (DE, Δt = 5 ms) spectra of two guests doped in **F-Ph-DL** solid in air at 77 K (λ_{ex} = 247 nm), w/w = a) 1%, b) 0.1%, c) 0.01%, d) 10ppm.

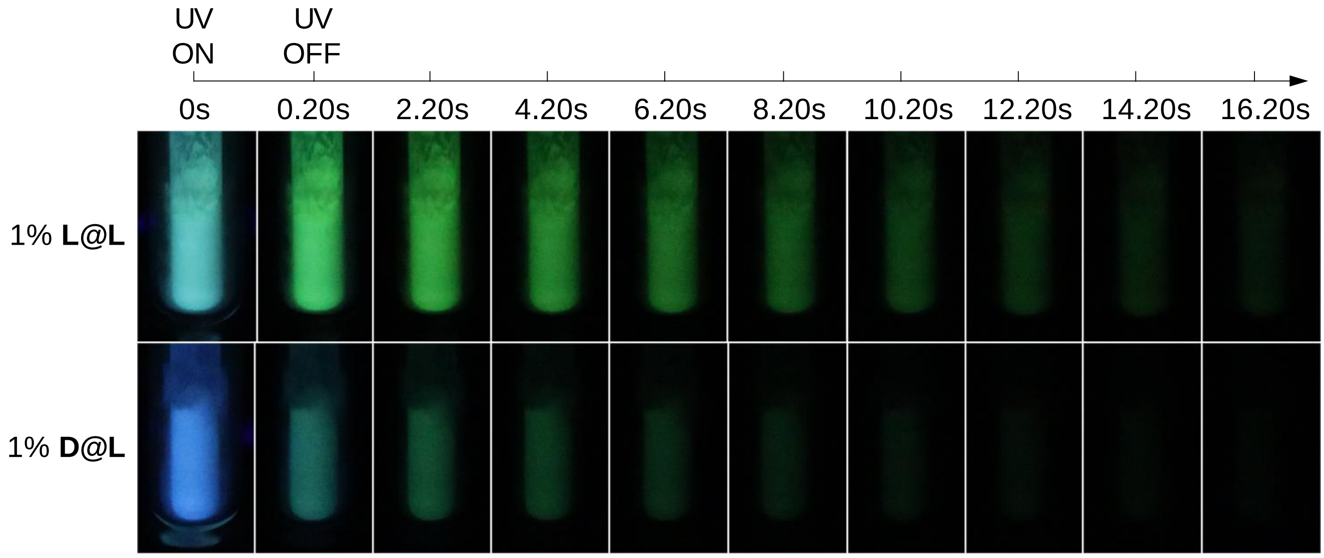

Supplementary Figure 28. Photographs of combinations of two guests doped in **F-Ph-L** (w/w = 1%) during and immediately after 254-nm light irradiation at 77 K.

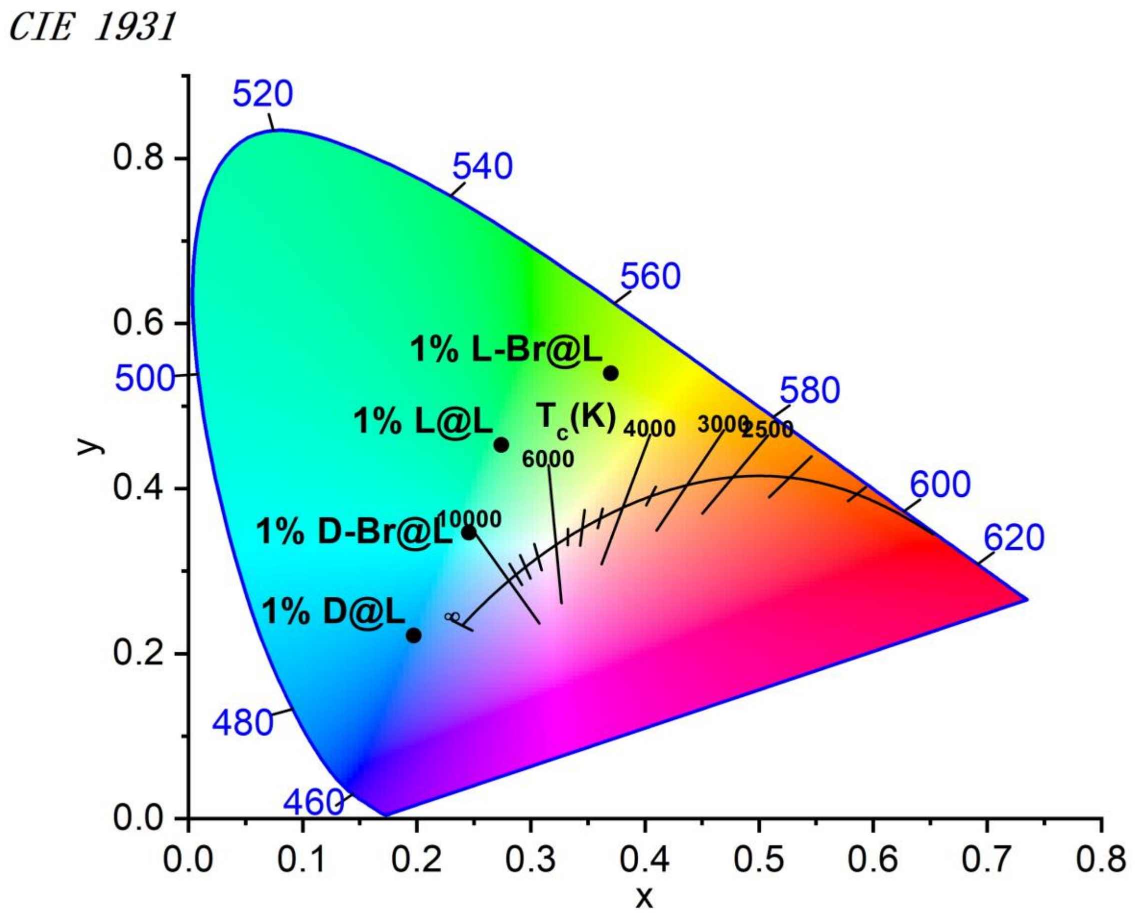

Supplementary Figure 29. CIE Figure of 1% **L@L, D@L, L-Br@L** and **D-Br@L** solid at 77 K (λ_{ex} = 247 nm).

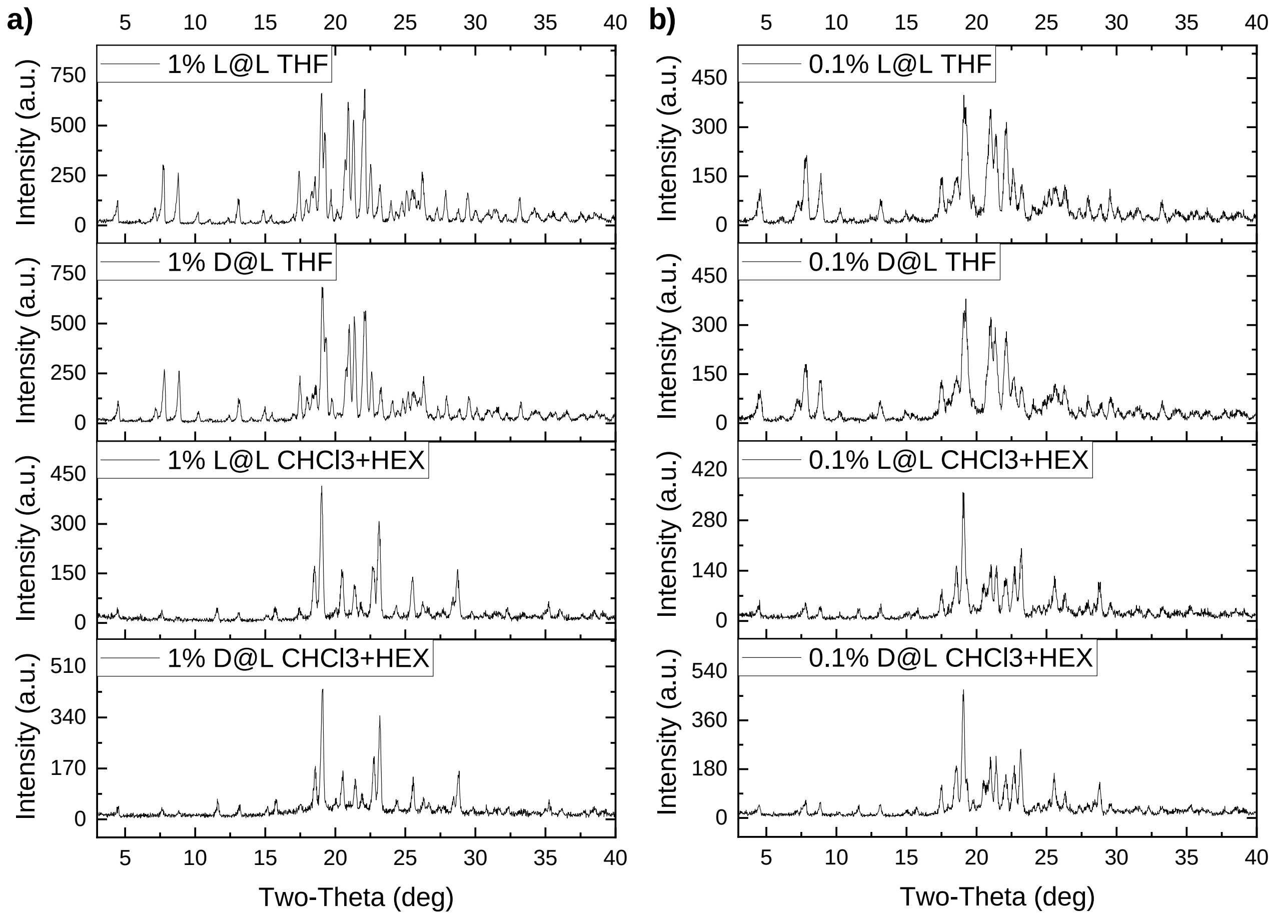

Supplementary Figure 30. Powder X-ray diffraction (PXRD) patterns of doped samples for different solvent systems, w/w = 1% for a) and 0.1% for b).

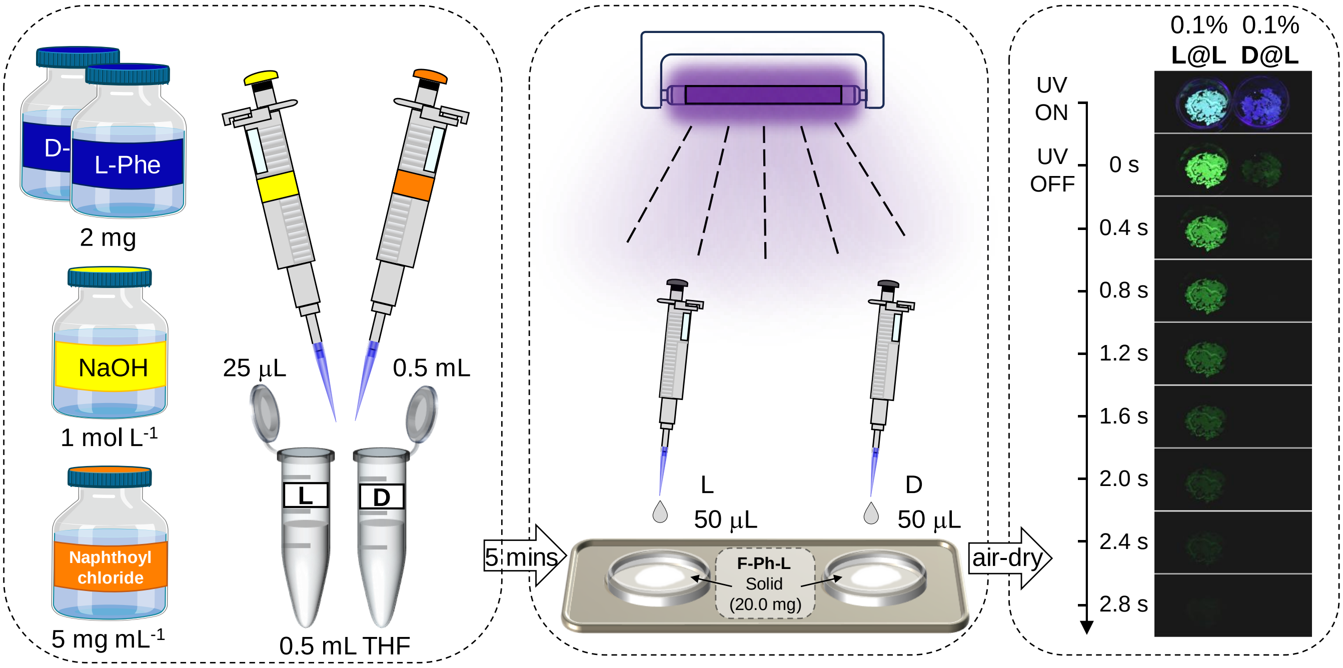

Supplementary Figure 31. Rapid test protocol for in-situ chiral discrimination of D- vs. L-phenylalanine. Left: D- or L-phenylalanine (2mg) was added in the Eppendorf tube with 0.5 mL THF. Then color-coded reagents were transferred by pipettes with corresponding colors in designated amounts for the protocol, including aqueous NaOH (1 mol L-1, yellow) and naphthoyl chloride (5 mg mL-1 in THF, orange), where the final test solution was allowed to react for 5 mins in the mixture solvent of THF and water. Middle: An aliquot of the diluted, acidized solution (50 μL or two drops) was transferred onto pre-fabricated **F-Ph-L** solid-state films (20.0 mg) on glass substrates. Right: Test substrates were air-dried (1-2 min) before being taken for UV examination, where after the 254-nm light irradiation only the phenylalanine with the same chirality of the test substrate exhibits long RTP afterglow. (The excitation light source is a hand-held UV lamp emitting at 254 nm with an input power of 6 watts. The distance between the lamp and the samples was maintained at ~10 centimeters, and the excitation time for obtaining delayed emission or afterglow in the samples was set to 10 seconds.)

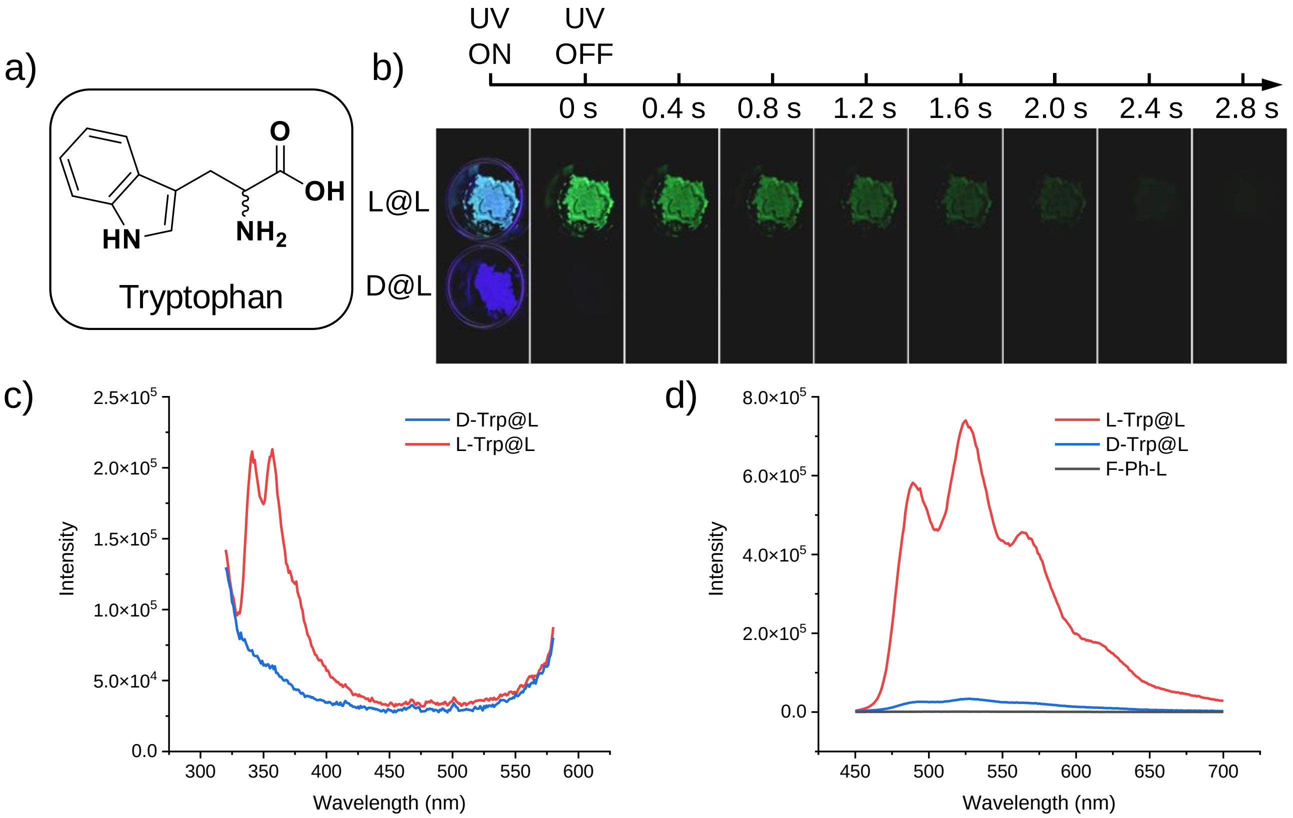

Supplementary Figure 32. a) Structure of Tryptophan. b) Photographs of combinations of two guests (crude products of the two chiral amino acids and 2-naphthoyl chloride) doped in **F-Ph-L** during and immediately after 254-nm light irradiation at 298 K. c) Steady-state spectra of two guests doped in the **F-Ph-L** solid medium in air at 298 K (λ_{ex} = 298 nm). d) Delayed emission (DE, Δt = 5 ms) spectra of two guests doped in **F-Ph-L** solid in air at 298 K (λ_{ex} = 247 nm). (The guest-to-host ratio is 0.1% for all samples in the solid state)

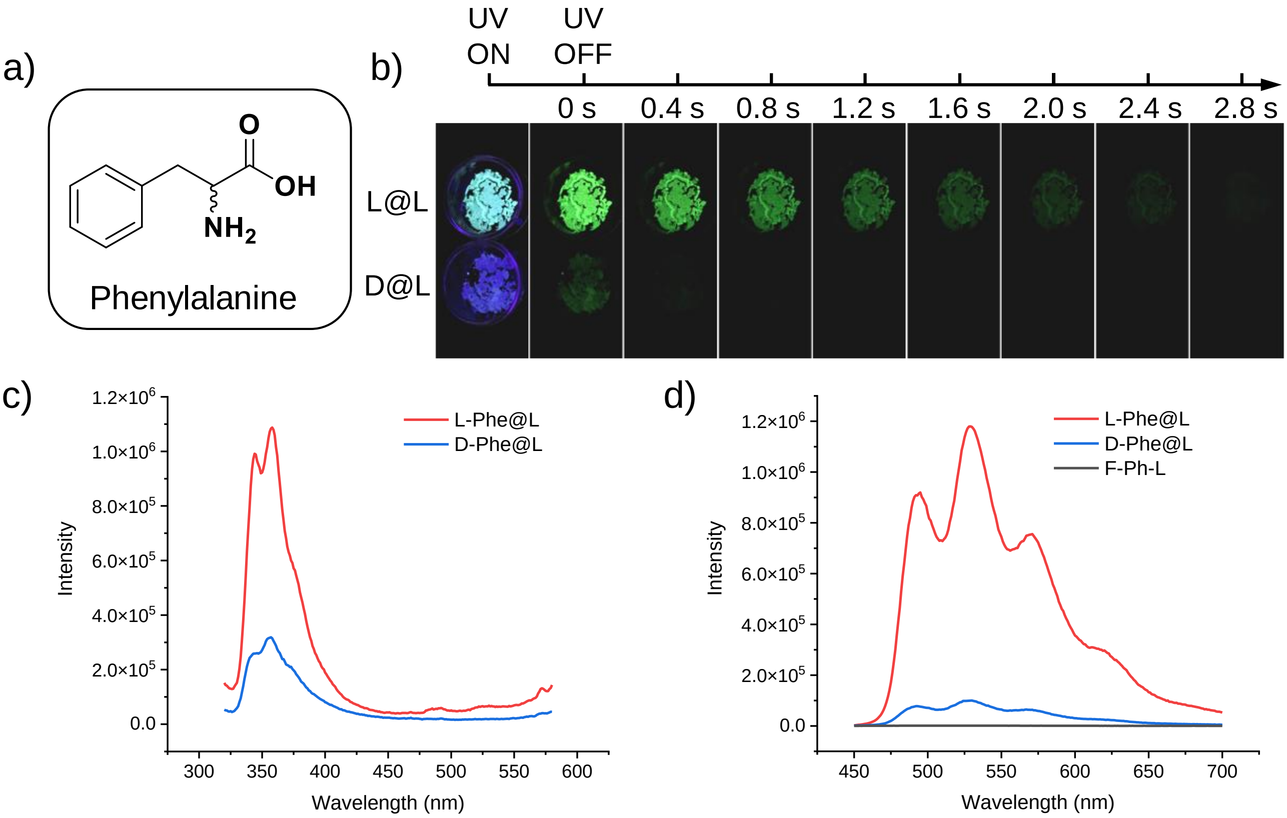

Supplementary Figure 33. a) Structure of Phenylalanine. b) Photographs of combinations of two guests (crude products of the two chiral amino acids and 2-naphthoyl chloride) doped in **F-Ph-L** during and immediately after 254-nm light irradiation at 298 K. c) Steady-state spectra of two guests doped in the **F-Ph-L** solid medium in air at 298 K (λ_{ex} = 298 nm). d) Delayed emission (DE, Δt = 5 ms) spectra of two guests doped in **F-Ph-L** solid in air at 298 K (λ_{ex} = 247 nm). (The guest-to-host ratio is 0.1% for all samples in the solid state)

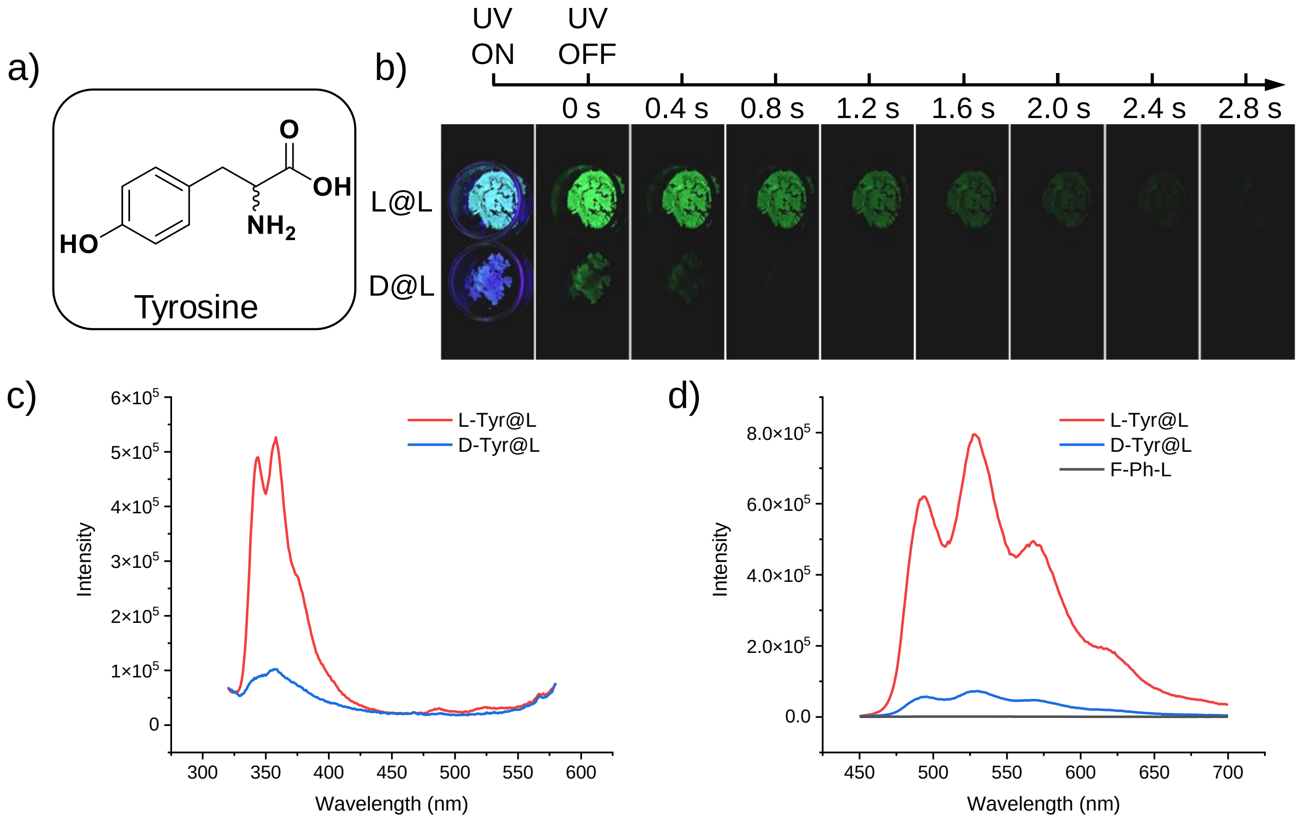

Supplementary Figure 34. a) Structure of Tyrosine. b) Photographs of combinations of two guests (crude products of the two chiral amino acids and 2-naphthoyl chloride) doped in **F-Ph-L** during and immediately after 254-nm light irradiation at 298 K. c) Steady-state spectra of two guests doped in the **F-Ph-L** solid medium in air at 298 K (λ_{ex} = 298 nm). d) Delayed emission (DE, Δt = 5 ms) spectra of two guests doped in **F-Ph-L** solid in air at 298 K (λ_{ex} = 247 nm). (The guest-to-host ratio is 0.1% for all samples in the solid state)

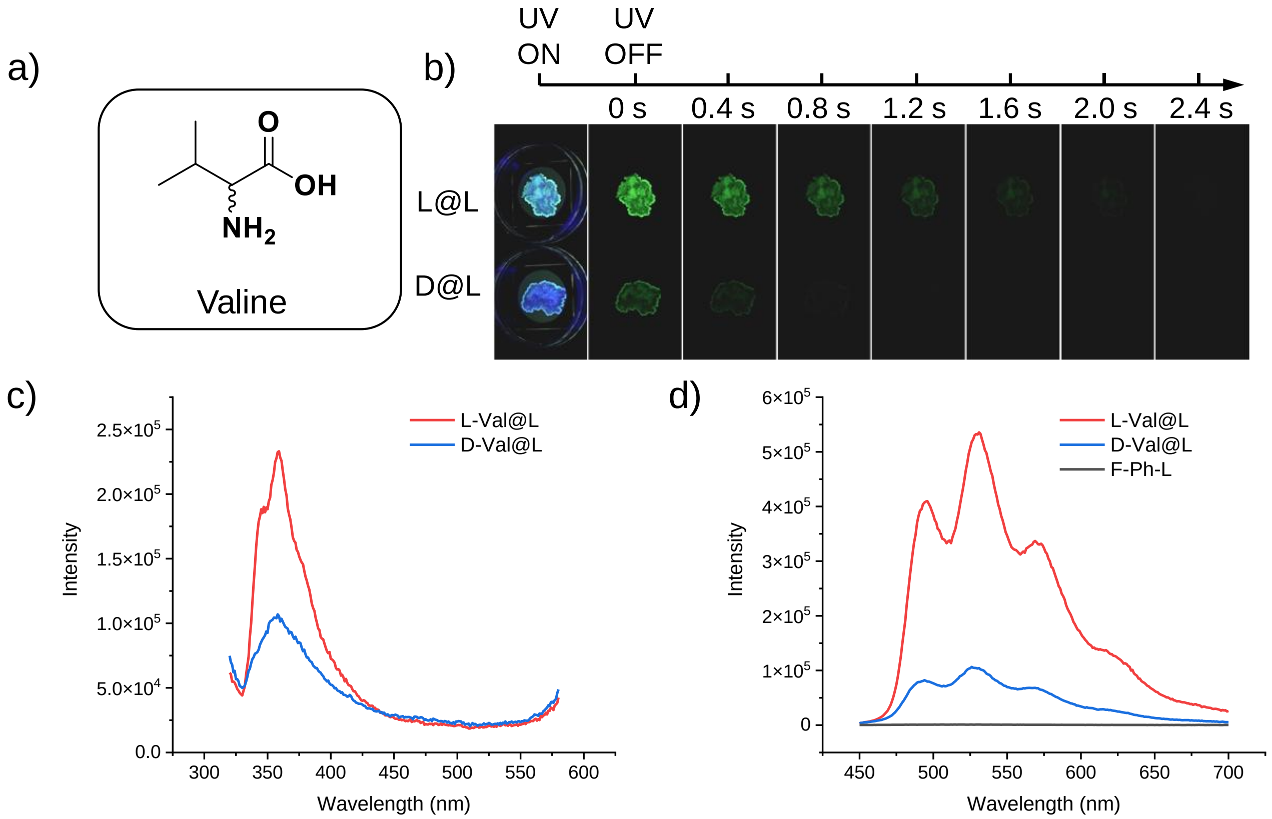

Supplementary Figure 35. a) Structure of Valine. b) Photographs of combinations of two guests (crude products of the two chiral amino acids and 2-naphthoyl chloride) doped in **F-Ph-L** during and immediately after 254-nm light irradiation at 298 K. c) Steady-state spectra of two guests doped in the **F-Ph-L** solid medium in air at 298 K (λ_{ex} = 298 nm). d) Delayed emission (DE, Δt = 5 ms) spectra of two guests doped in **F-Ph-L** solid in air at 298 K (λ_{ex} = 247 nm). (The guest-to-host ratio is 0.1% for all samples in the solid state)

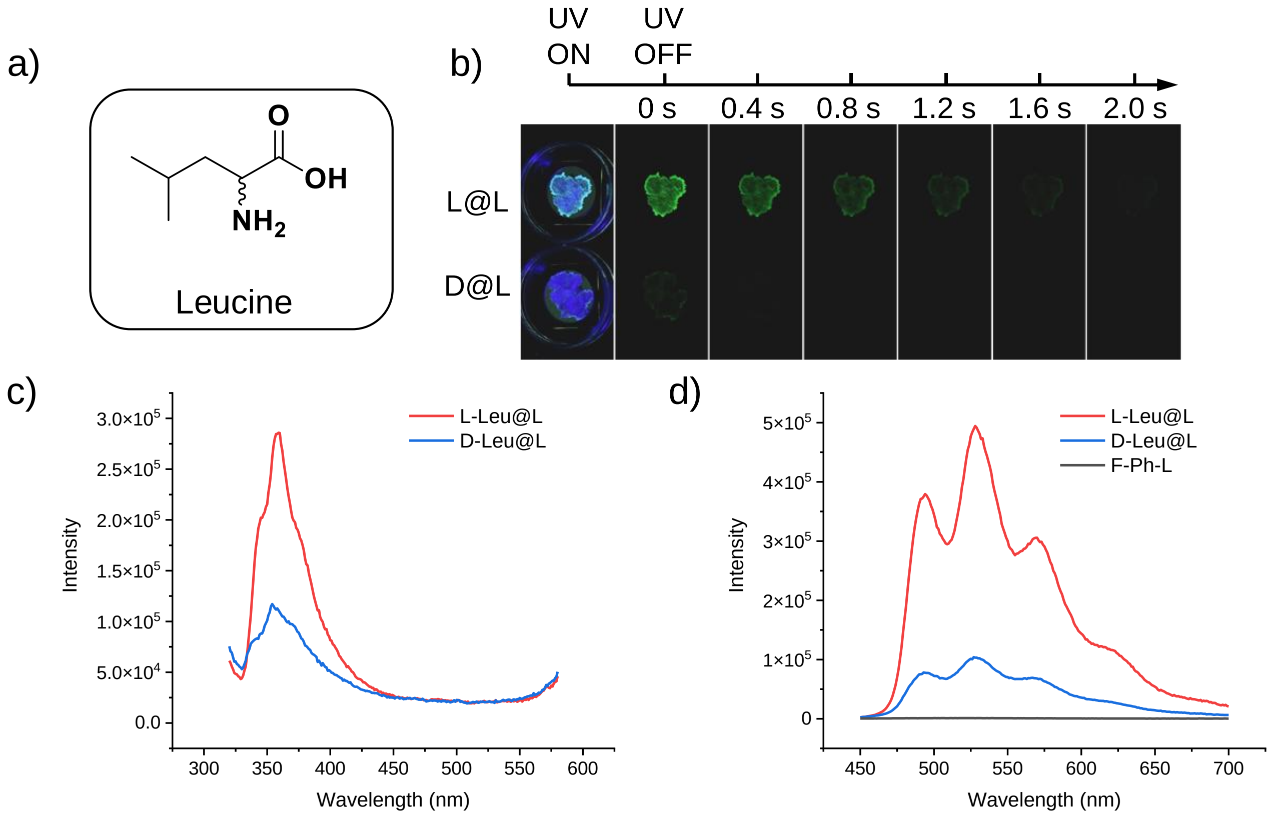

Supplementary Figure 36. a) Structure of Leucine. b) Photographs of combinations of two guests (crude products of the two chiral amino acids and 2-naphthoyl chloride) doped in **F-Ph-L** during and immediately after 254-nm light irradiation at 298 K. c) Steady-state spectra of two guests doped in the **F-Ph-L** solid medium in air at 298 K (λ_{ex} = 298 nm). d) Delayed emission (DE, Δt = 5 ms) spectra of two guests doped in **F-Ph-L** solid in air at 298 K (λ_{ex} = 247 nm). (The guest-to-host ratio is 0.1% for all samples in the solid state)

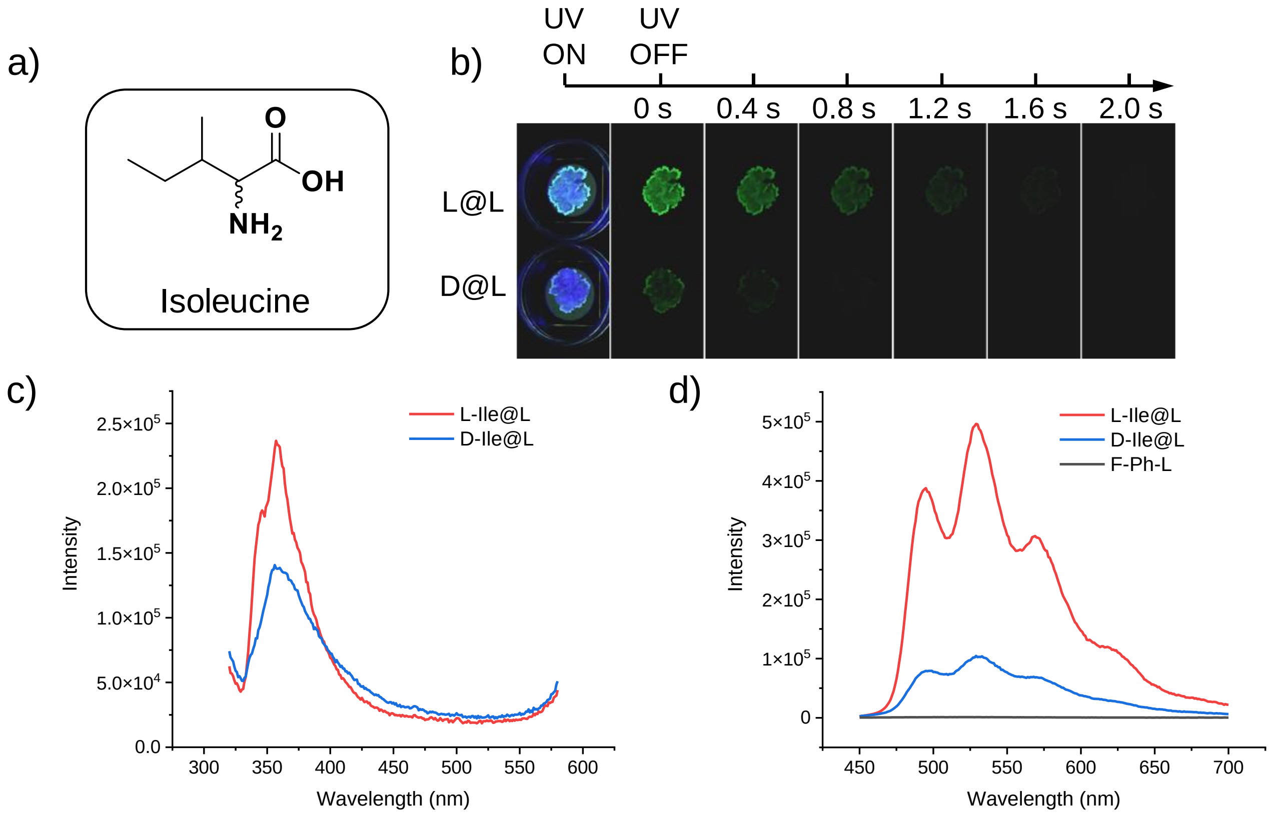

Supplementary Figure 37. a) Structure of Isoleucine. b) Photographs of combinations of two guests (crude products of the two chiral amino acids and 2-naphthoyl chloride) doped in **F-Ph-L** during and immediately after 254-nm light irradiation at 298 K. c) Steady-state spectra of two guests doped in the **F-Ph-L** solid medium in air at 298 K (λ_{ex} = 298 nm). d) Delayed emission (DE, Δt = 5 ms) spectra of two guests doped in **F-Ph-L** solid in air at 298 K (λ_{ex} = 247 nm). (The guest-to-host ratio is 0.1% for all samples in the solid state)

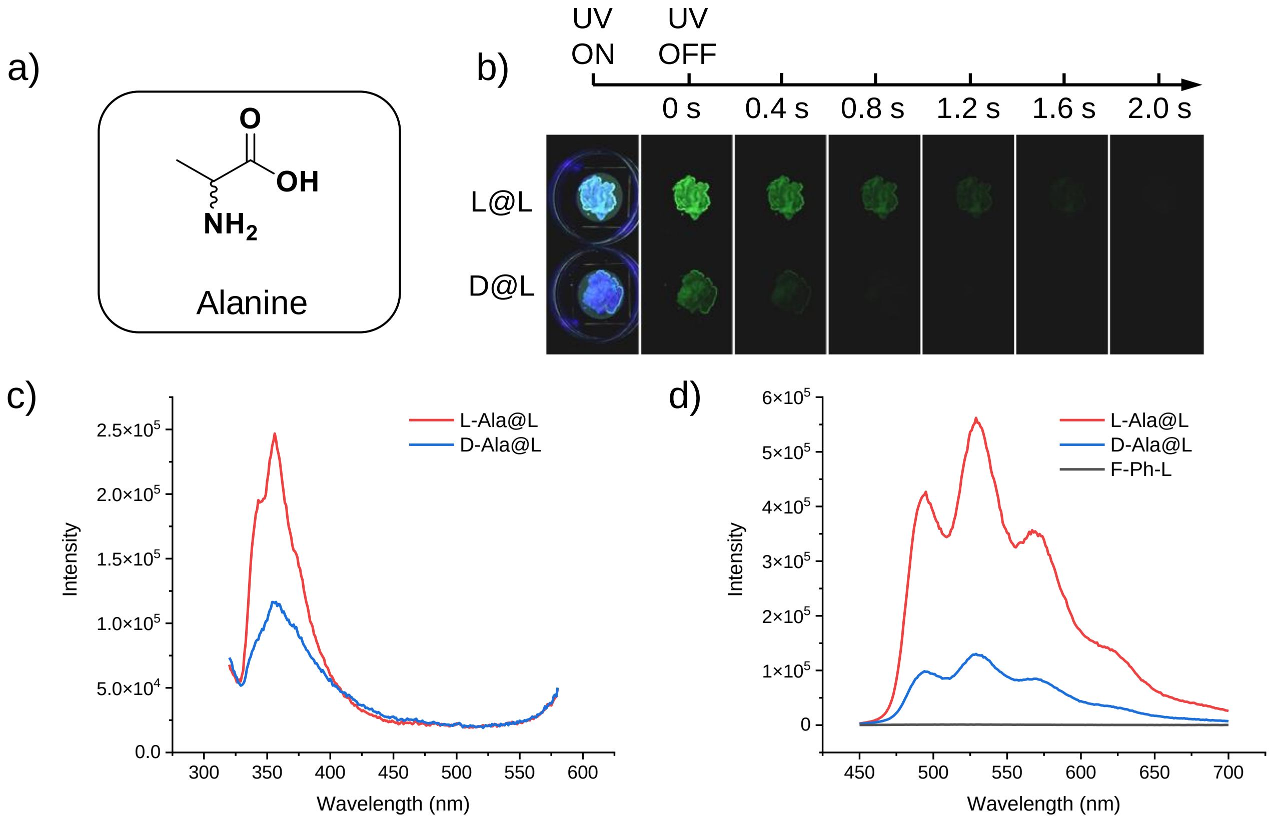

Supplementary Figure 38. a) Structure of Alanine. b) Photographs of combinations of two guests (crude products of the two chiral amino acids and 2-naphthoyl chloride) doped in **F-Ph-L** during and immediately after 254-nm light irradiation at 298 K. c) Steady-state spectra of two guests doped in the **F-Ph-L** solid medium in air at 298 K (λ_{ex} = 298 nm). d) Delayed emission (DE, Δt = 5 ms) spectra of two guests doped in **F-Ph-L** solid in air at 298 K (λ_{ex} = 247 nm). (The guest-to-host ratio is 0.1% for all samples in the solid state)

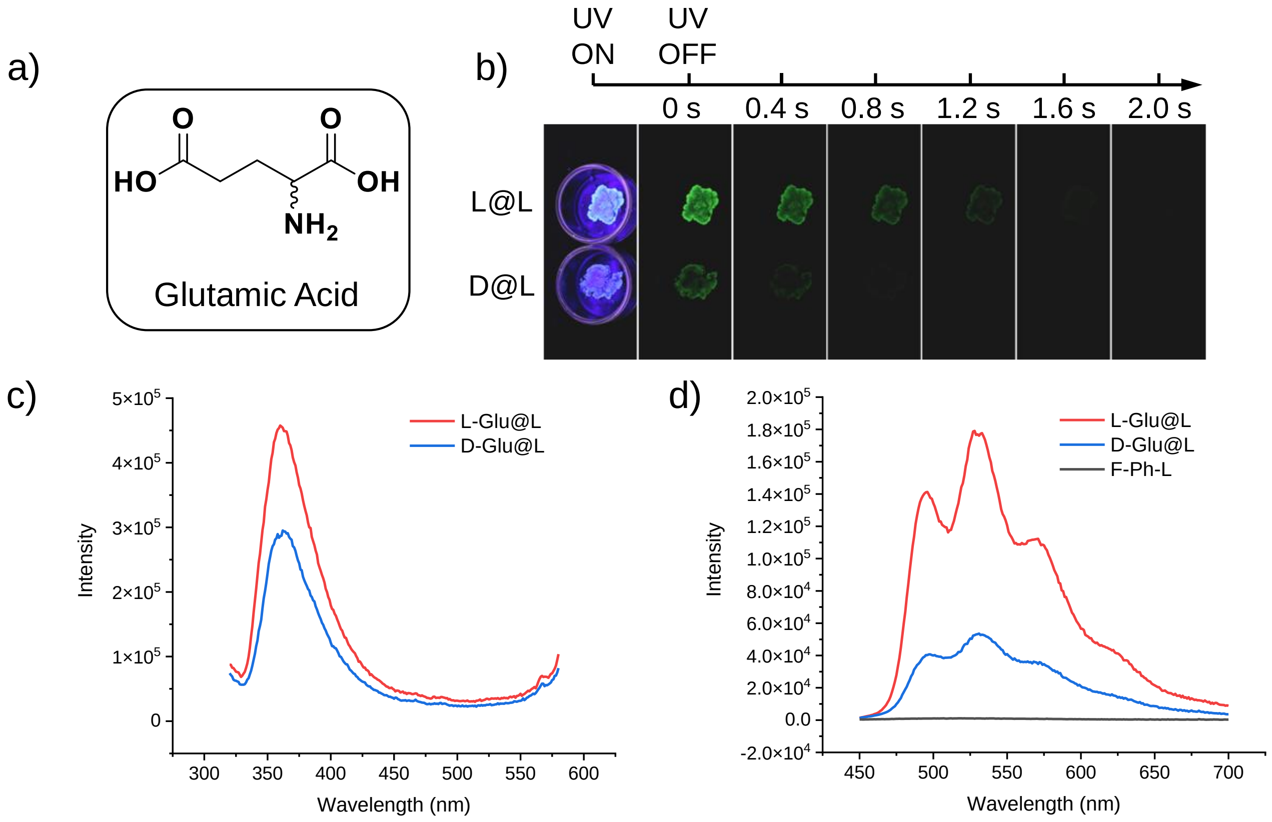

Supplementary Figure 39. a) Structure of Glutamic Acid. b) Photographs of combinations of two guests (crude products of the two chiral amino acids and 2-naphthoyl chloride) doped in **F-Ph-L** during and immediately after 254-nm light irradiation at 298 K. c) Steady-state spectra of two guests doped in the **F-Ph-L** solid medium in air at 298 K (λ_{ex} = 298 nm). d) Delayed emission (DE, Δt = 5 ms) spectra of two guests doped in **F-Ph-L** solid in air at 298 K (λ_{ex} = 247 nm). (The guest-to-host ratio is 0.1% for all samples in the solid state)

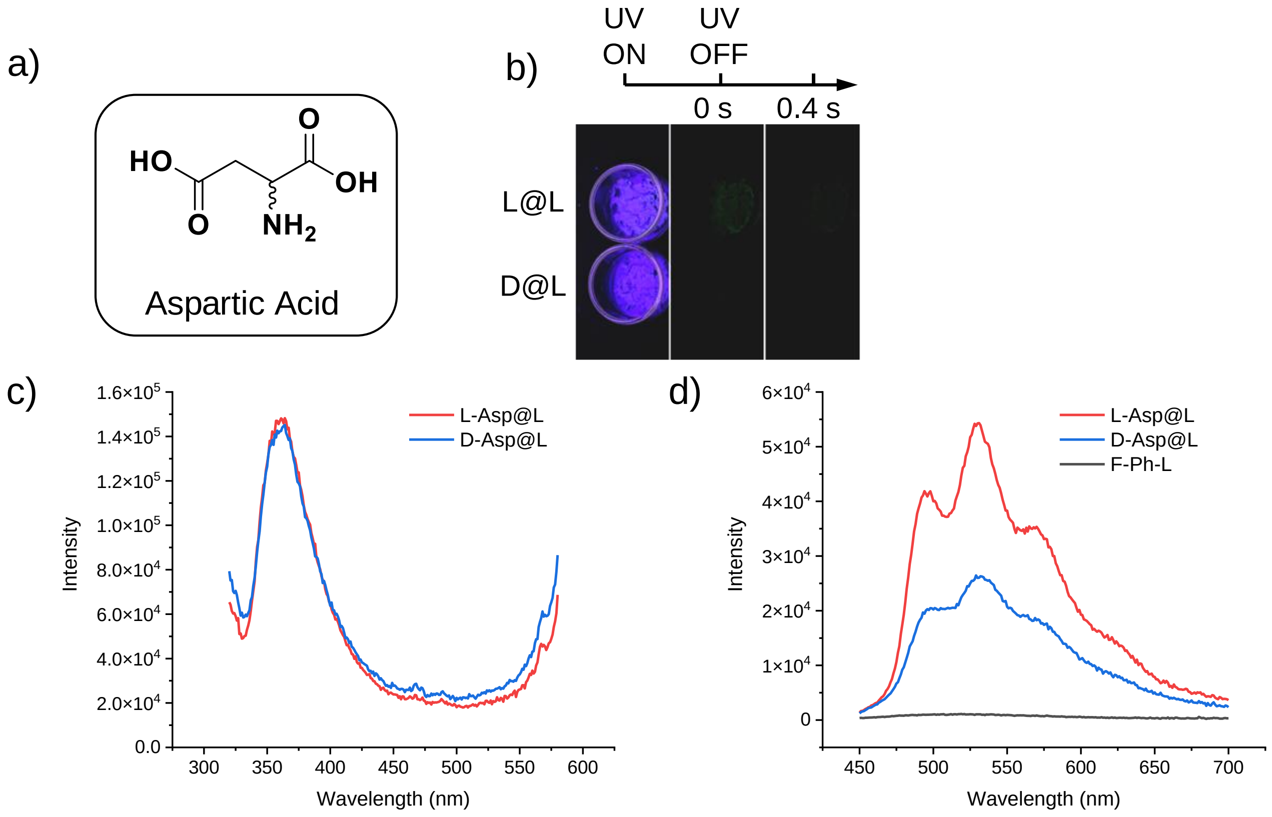

Supplementary Figure 40. a) Structure of Aspartic Acid. b) Photographs of combinations of two guests (crude products of the two chiral amino acids and 2-naphthoyl chloride) doped in **F-Ph-L** during and immediately after 254-nm light irradiation at 298 K. c) Steady-state spectra of two guests doped in the **F-Ph-L** solid medium in air at 298 K (λ_{ex} = 298 nm). d) Delayed emission (DE, Δt = 5 ms) spectra of two guests doped in **F-Ph-L** solid in air at 298 K (λ_{ex} = 247 nm). (The guest-to-host ratio is 0.1% for all samples in the solid state)

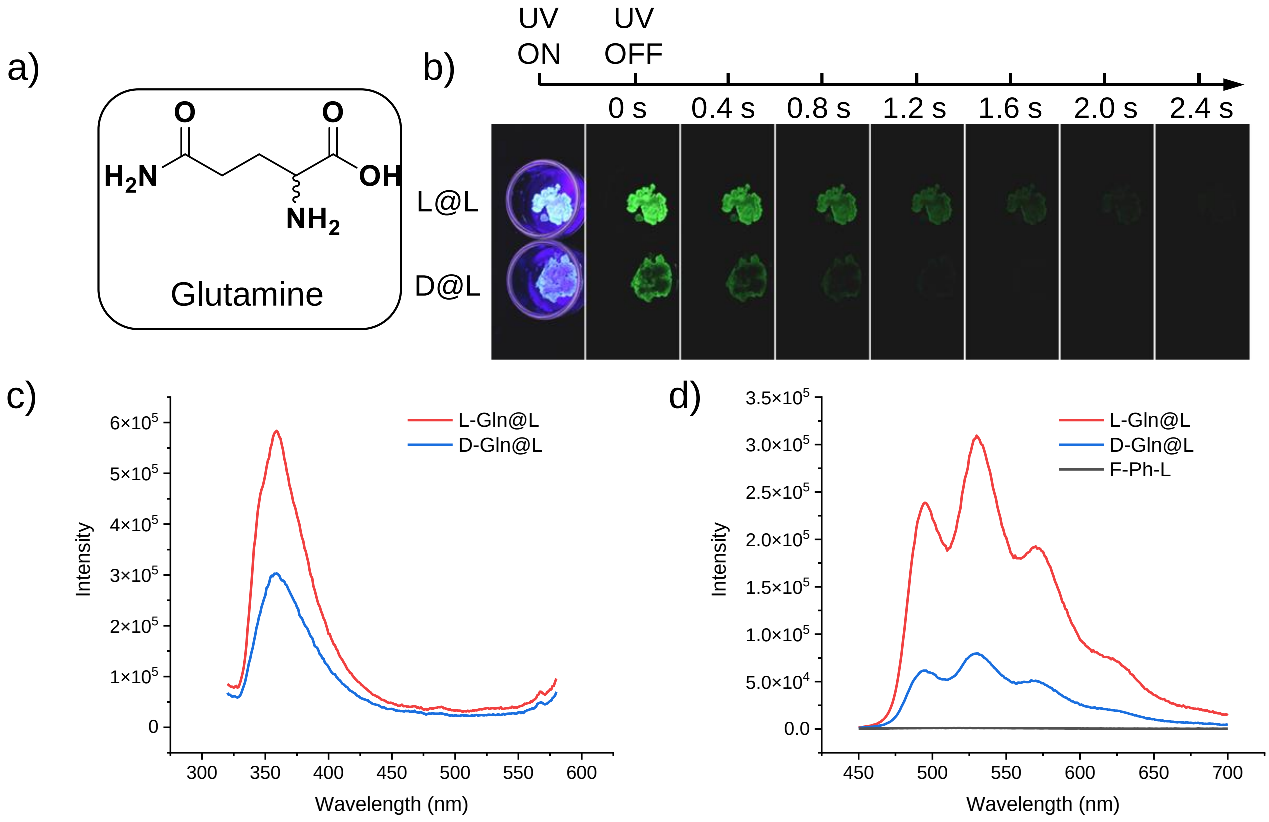

Supplementary Figure 41. a) Structure of Glutamine. b) Photographs of combinations of two guests (crude products of the two chiral amino acids and 2-naphthoyl chloride) doped in **F-Ph-L** during and immediately after 254-nm light irradiation at 298 K. c) Steady-state spectra of two guests doped in the **F-Ph-L** solid medium in air at 298 K (λ_{ex} = 298 nm). d) Delayed emission (DE, Δt = 5 ms) spectra of two guests doped in **F-Ph-L** solid in air at 298 K (λ_{ex} = 247 nm). (The guest-to-host ratio is 0.1% for all samples in the solid state)

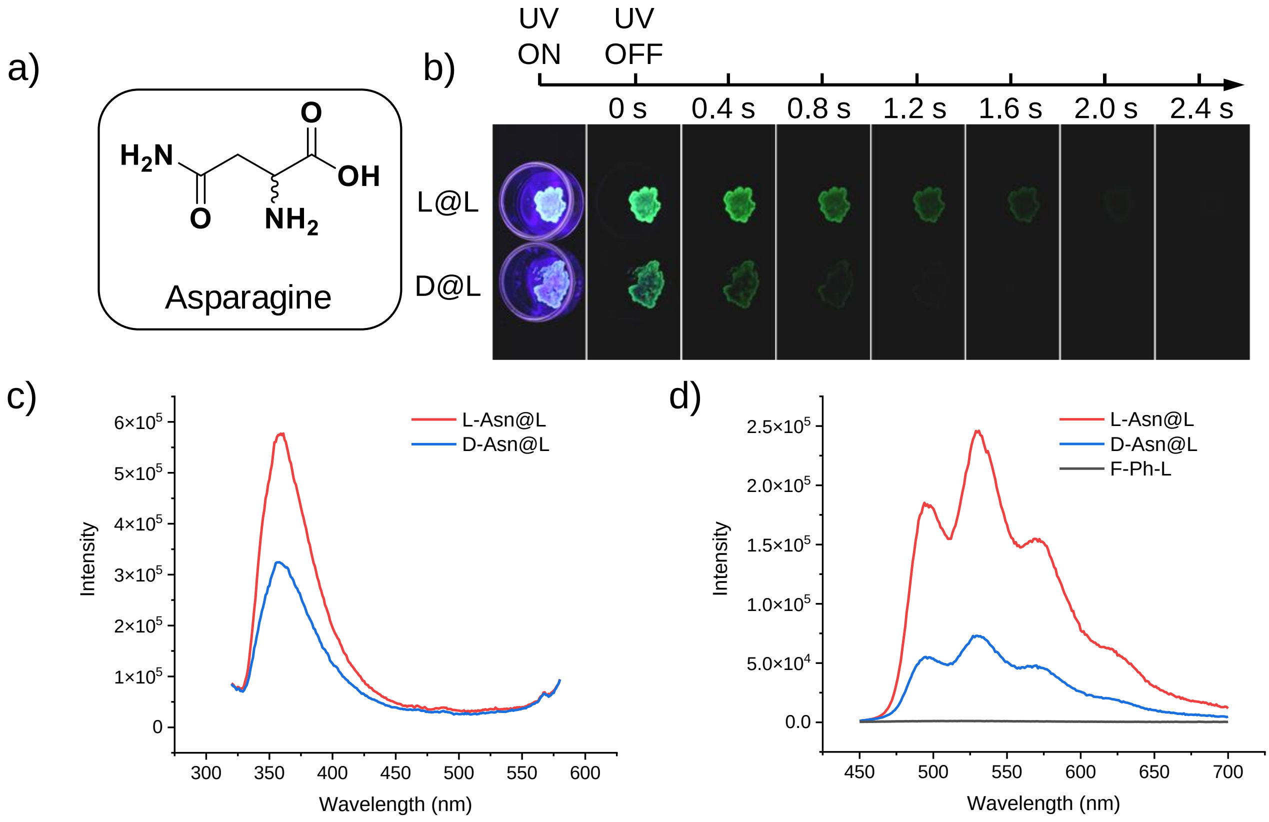

Supplementary Figure 42. a) Structure of Asparagine. b) Photographs of combinations of two guests (crude products of the two chiral amino acids and 2-naphthoyl chloride) doped in **F-Ph-L** during and immediately after 254-nm light irradiation at 298 K. c) Steady-state spectra of two guests doped in the **F-Ph-L** solid medium in air at 298 K (λ_{ex} = 298 nm). d) Delayed emission (DE, Δt = 5 ms) spectra of two guests doped in **F-Ph-L** solid in air at 298 K (λ_{ex} = 247 nm). (The guest-to-host ratio is 0.1% for all samples in the solid state)

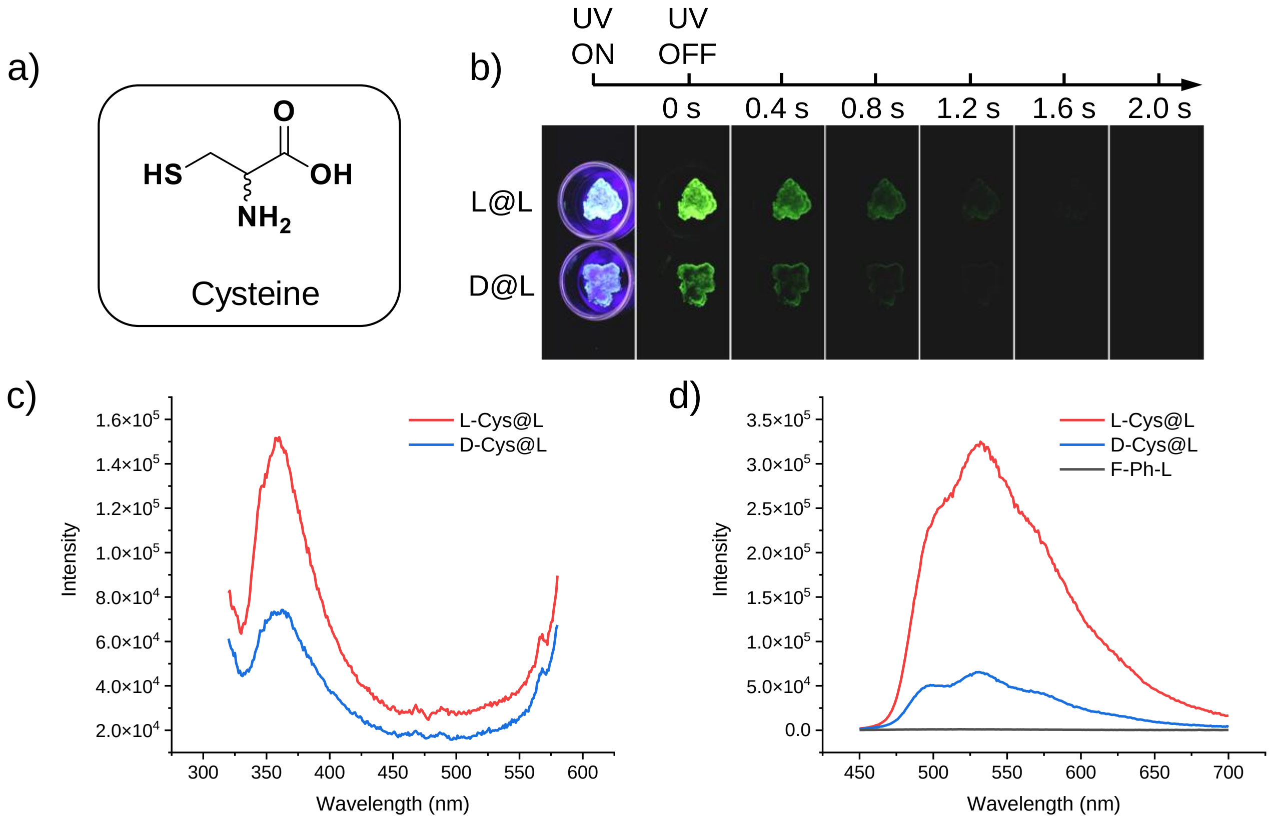

Supplementary Figure 43. a) Structure of Cysteine. b) Photographs of combinations of two guests (crude products of the two chiral amino acids and 2-naphthoyl chloride) doped in **F-Ph-L** during and immediately after 254-nm light irradiation at 298 K. c) Steady-state spectra of two guests doped in the **F-Ph-L** solid medium in air at 298 K (λ_{ex} = 298 nm). d) Delayed emission (DE, Δt = 5 ms) spectra of two guests doped in **F-Ph-L** solid in air at 298 K (λ_{ex} = 247 nm). (The guest-to-host ratio is 0.1% for all samples in the solid state)

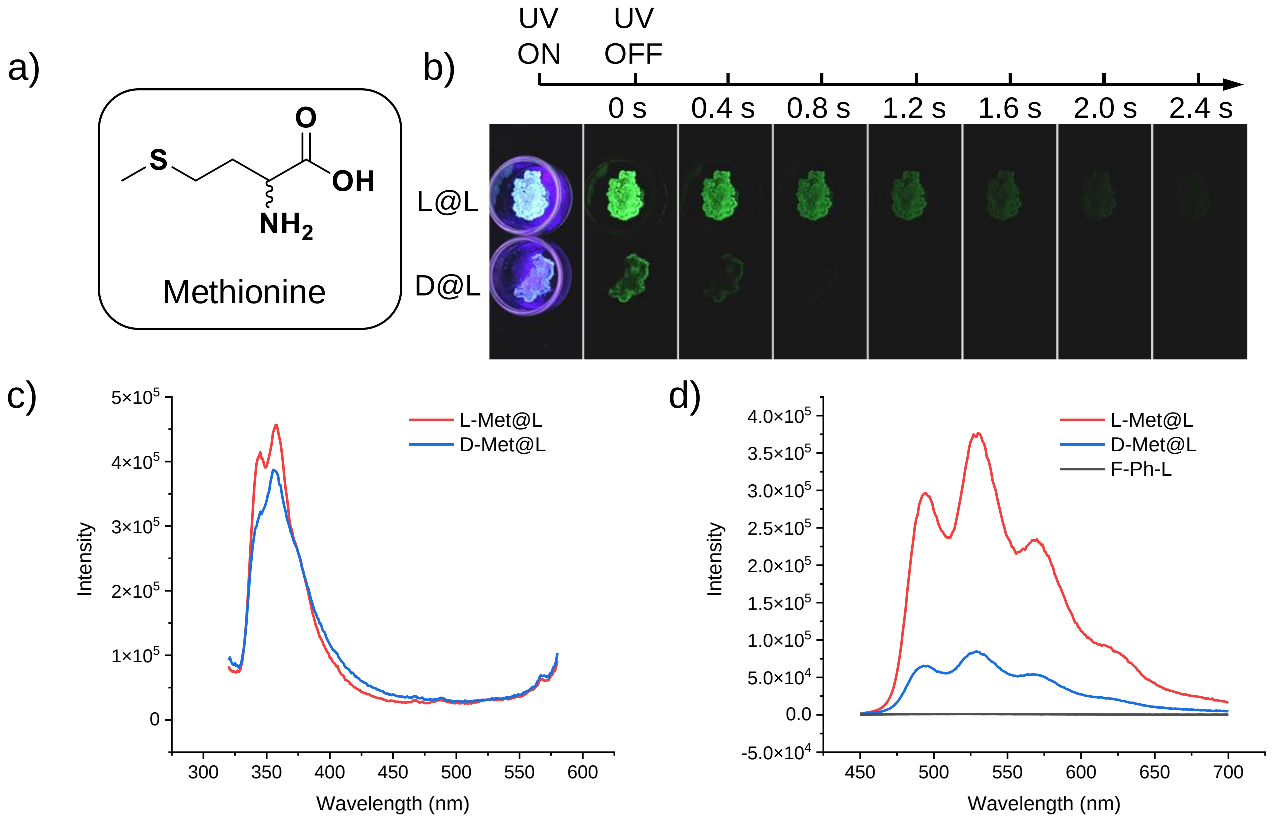

Supplementary Figure 44. a) Structure of Methionine. b) Photographs of combinations of two guests (crude products of the two chiral amino acids and 2-naphthoyl chloride) doped in **F-Ph-L** during and immediately after 254-nm light irradiation at 298 K. c) Steady-state spectra of two guests doped in the **F-Ph-L** solid medium in air at 298 K (λ_{ex} = 298 nm). d) Delayed emission (DE, Δt = 5 ms) spectra of two guests doped in **F-Ph-L** solid in air at 298 K (λ_{ex} = 247 nm). (The guest-to-host ratio is 0.1% for all samples in the solid state)

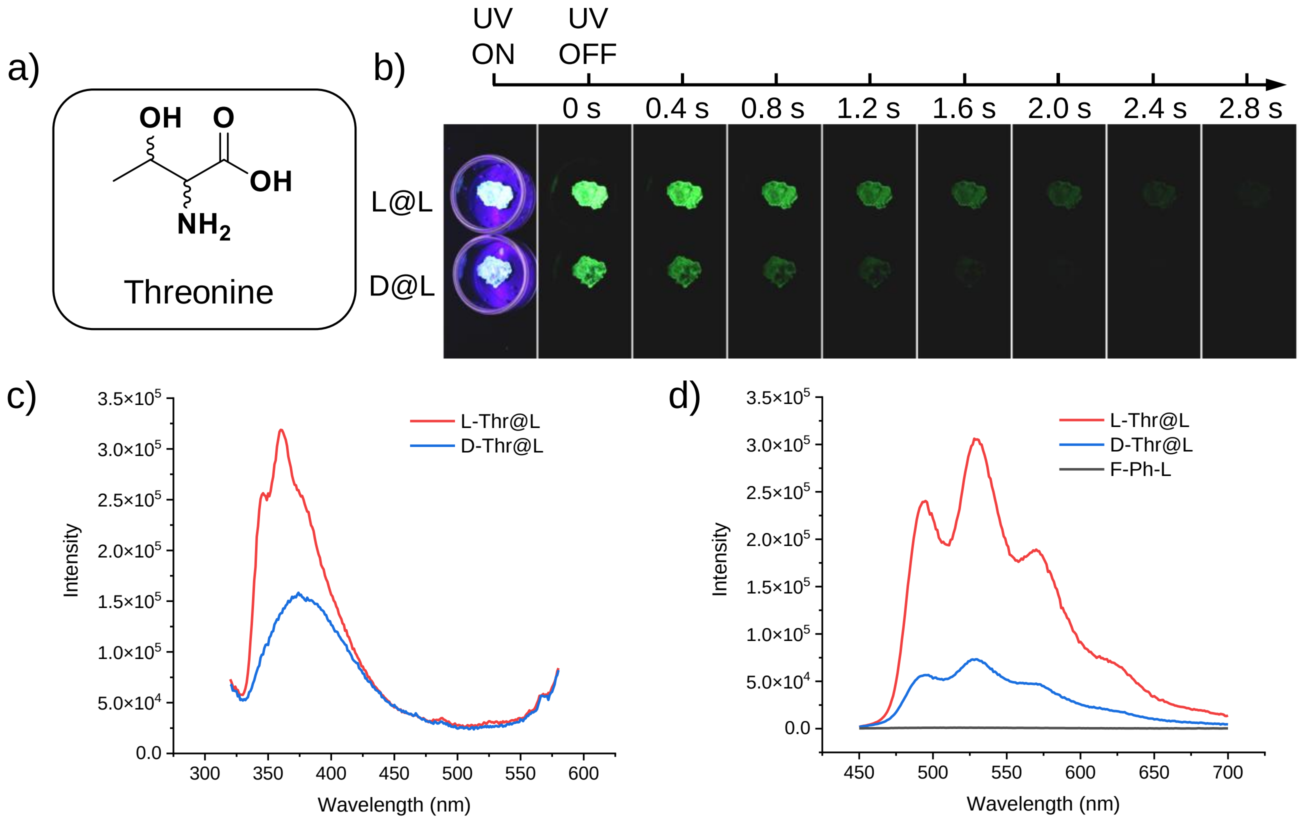

Supplementary Figure 45. a) Structure of Threonine. b) Photographs of combinations of two guests (crude products of the two chiral amino acids and 2-naphthoyl chloride) doped in **F-Ph-L** during and immediately after 254-nm light irradiation at 298 K. c) Steady-state spectra of two guests doped in the **F-Ph-L** solid medium in air at 298 K (λ_{ex} = 298 nm). d) Delayed emission (DE, Δt = 5 ms) spectra of two guests doped in **F-Ph-L** solid in air at 298 K (λ_{ex} = 247 nm). (The guest-to-host ratio is 0.1% for all samples in the solid state)

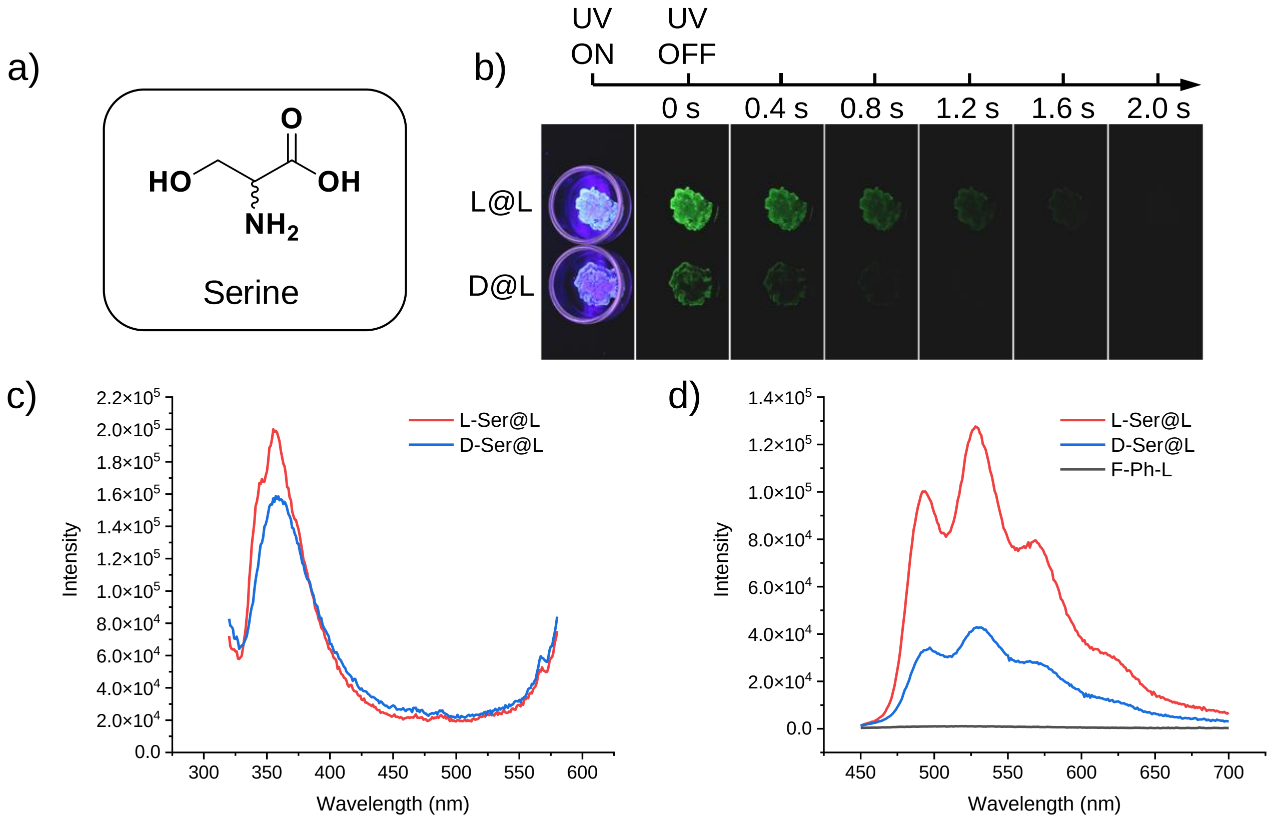

Supplementary Figure 46. a) Structure of Serine. b) Photographs of combinations of two guests (crude products of the two chiral amino acids and 2-naphthoyl chloride) doped in **F-Ph-L** during and immediately after 254-nm light irradiation at 298 K. c) Steady-state spectra of two guests doped in the **F-Ph-L** solid medium in air at 298 K (λ_{ex} = 298 nm). d) Delayed emission (DE, Δt = 5 ms) spectra of two guests doped in **F-Ph-L** solid in air at 298 K (λ_{ex} = 247 nm). (The guest-to-host ratio is 0.1% for all samples in the solid state)

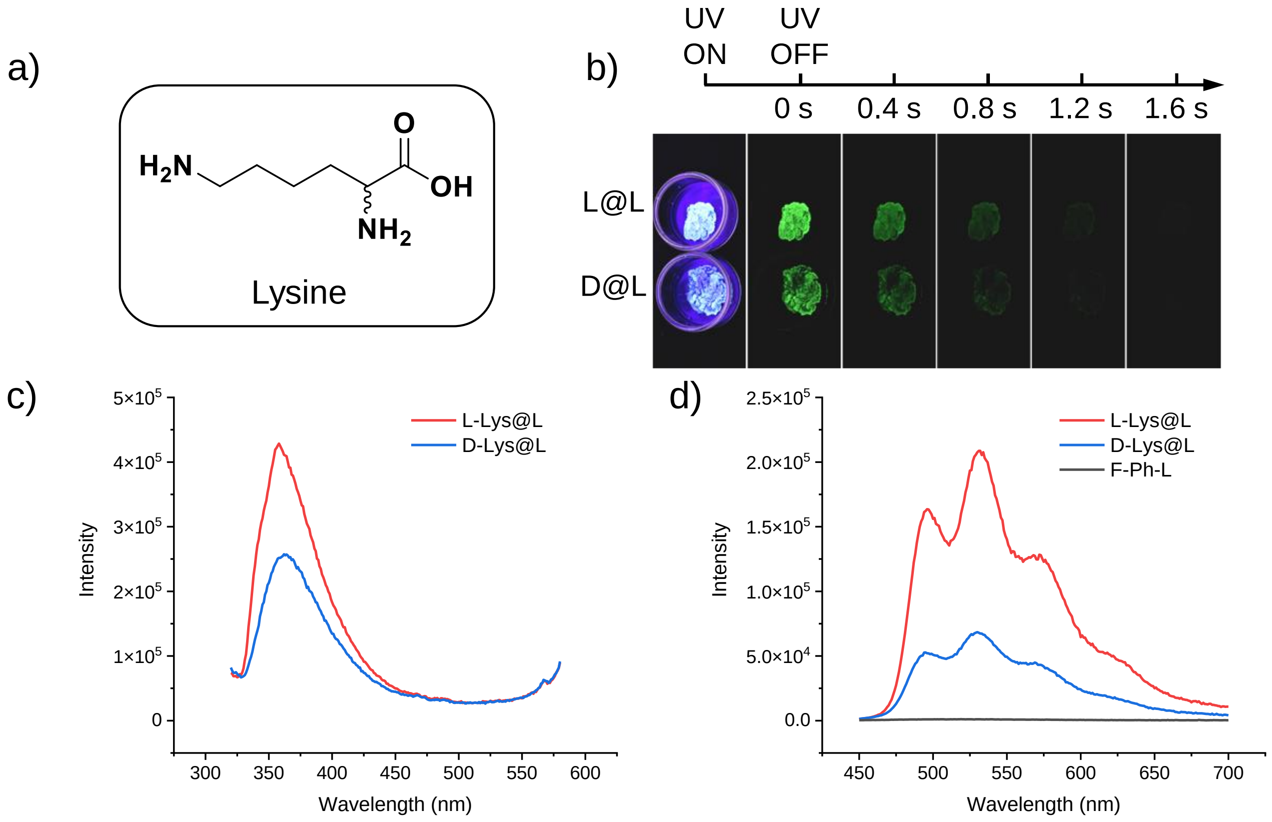

Supplementary Figure 47. a) Structure of Lysine. b) Photographs of combinations of two guests (crude products of the two chiral amino acids and 2-naphthoyl chloride) doped in **F-Ph-L** during and immediately after 254-nm light irradiation at 298 K. c) Steady-state spectra of two guests doped in the **F-Ph-L** solid medium in air at 298 K (λ_{ex} = 298 nm). d) Delayed emission (DE, Δt = 5 ms) spectra of two guests doped in **F-Ph-L** solid in air at 298 K (λ_{ex} = 247 nm). (The guest-to-host ratio is 0.1% for all samples in the solid state)

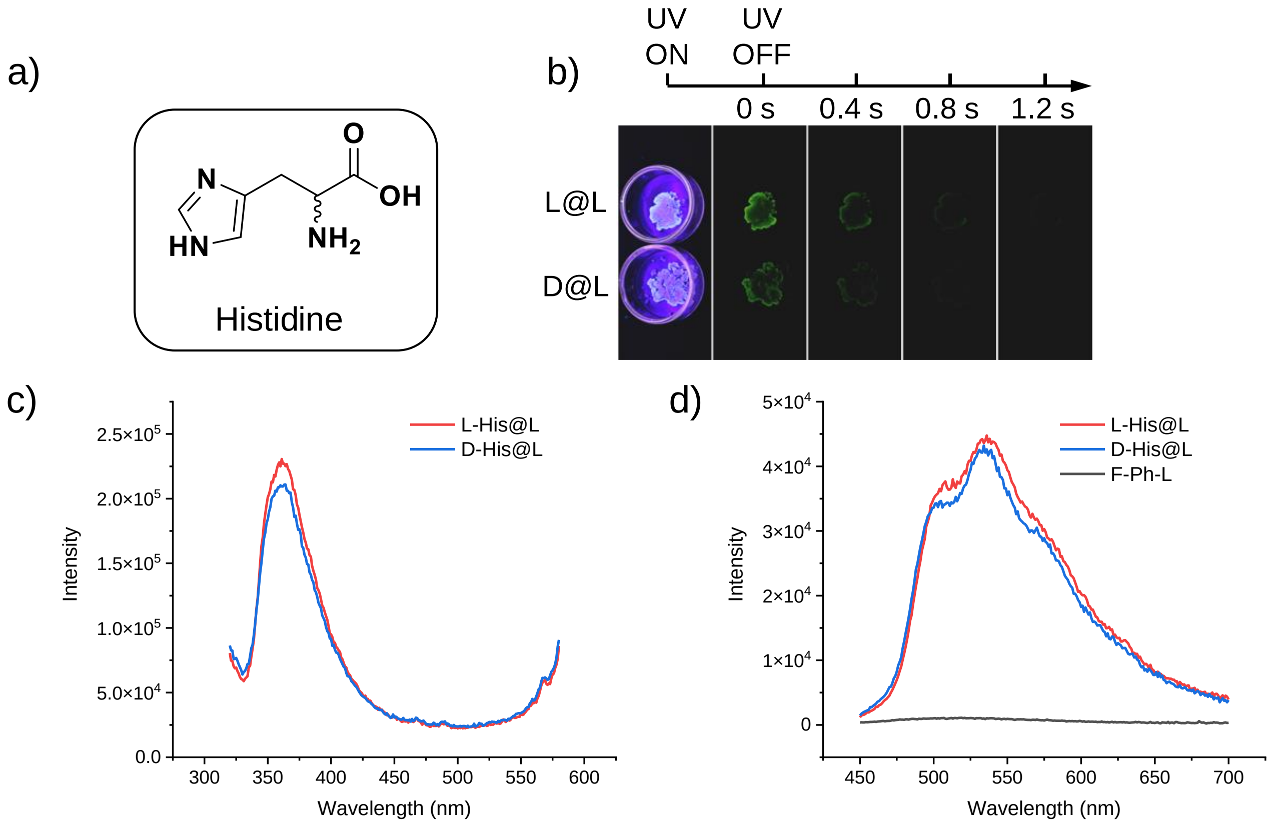

Supplementary Figure 48. a) Structure of Histidine. b) Photographs of combinations of two guests (crude products of the two chiral amino acids and 2-naphthoyl chloride) doped in **F-Ph-L** during and immediately after 254-nm light irradiation at 298 K. c) Steady-state spectra of two guests doped in the **F-Ph-L** solid medium in air at 298 K (λ_{ex} = 298 nm). d) Delayed emission (DE, Δt = 5 ms) spectra of two guests doped in **F-Ph-L** solid in air at 298 K (λ_{ex} = 247 nm). (The guest-to-host ratio is 0.1% for all samples in the solid state)

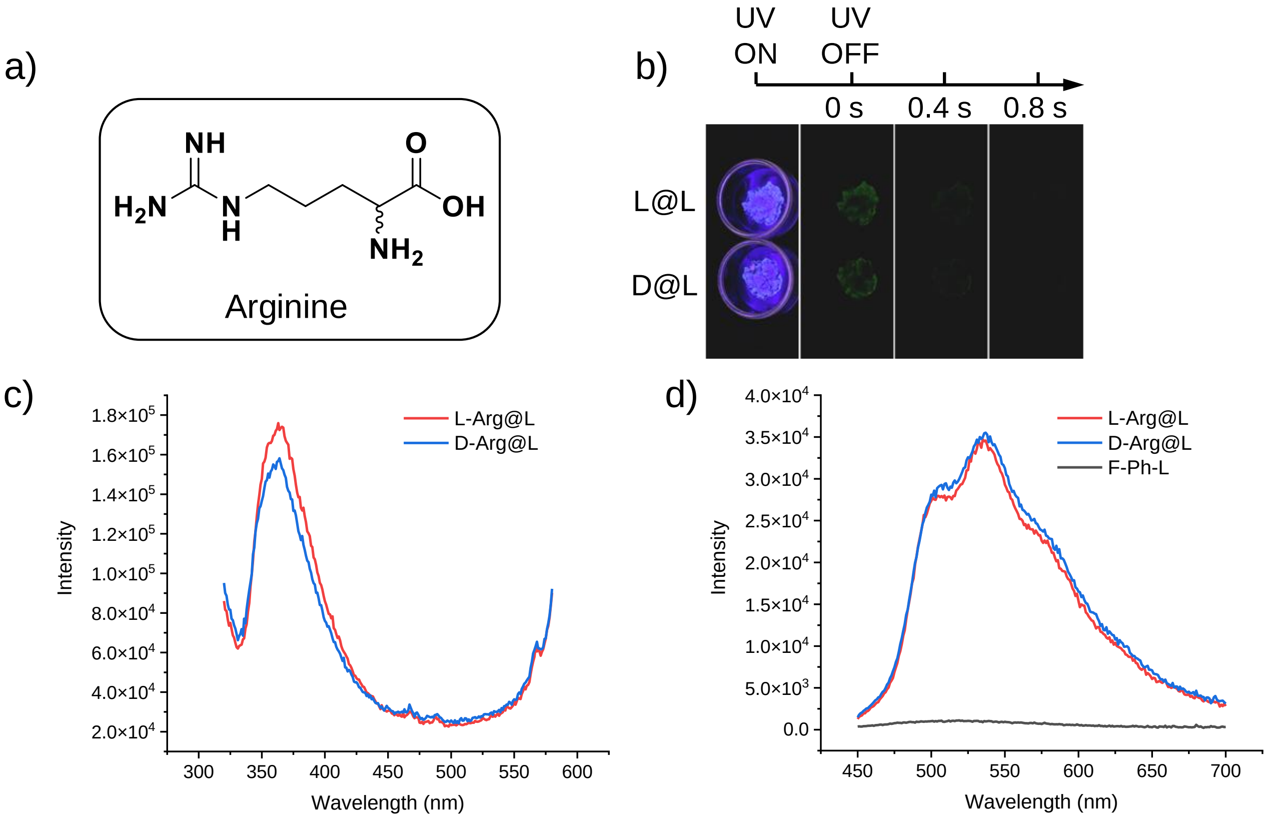

Supplementary Figure 49. a) Structure of Arginine. b) Photographs of combinations of two guests (crude products of the two chiral amino acids and 2-naphthoyl chloride) doped in **F-Ph-L** during and immediately after 254-nm light irradiation at 298 K. c) Steady-state spectra of two guests doped in the **F-Ph-L** solid medium in air at 298 K (λ_{ex} = 298 nm). d) Delayed emission (DE, Δt = 5 ms) spectra of two guests doped in **F-Ph-L** solid in air at 298 K (λ_{ex} = 247 nm). (The guest-to-host ratio is 0.1% for all samples in the solid state)

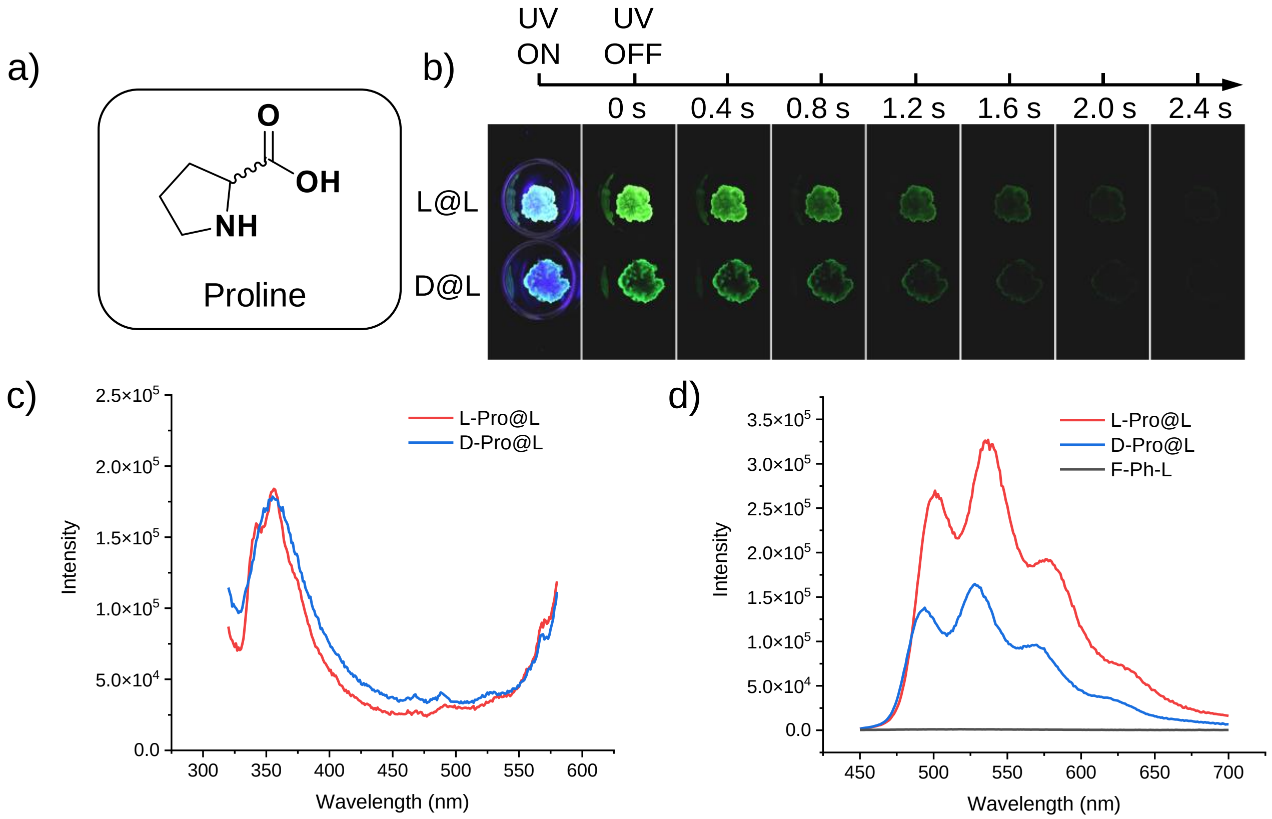

Supplementary Figure 50. a) Structure of Proline. b) Photographs of combinations of two guests (crude products of the two chiral amino acids and 2-naphthoyl chloride) doped in **F-Ph-L** during and immediately after 254-nm light irradiation at 298 K. c) Steady-state spectra of two guests doped in the **F-Ph-L** solid medium in air at 298 K (λ_{ex} = 298 nm). d) Delayed emission (DE, Δt = 5 ms) spectra of two guests doped in **F-Ph-L** solid in air at 298 K (λ_{ex} = 247 nm). (The guest-to-host ratio is 0.1% for all samples in the solid state)

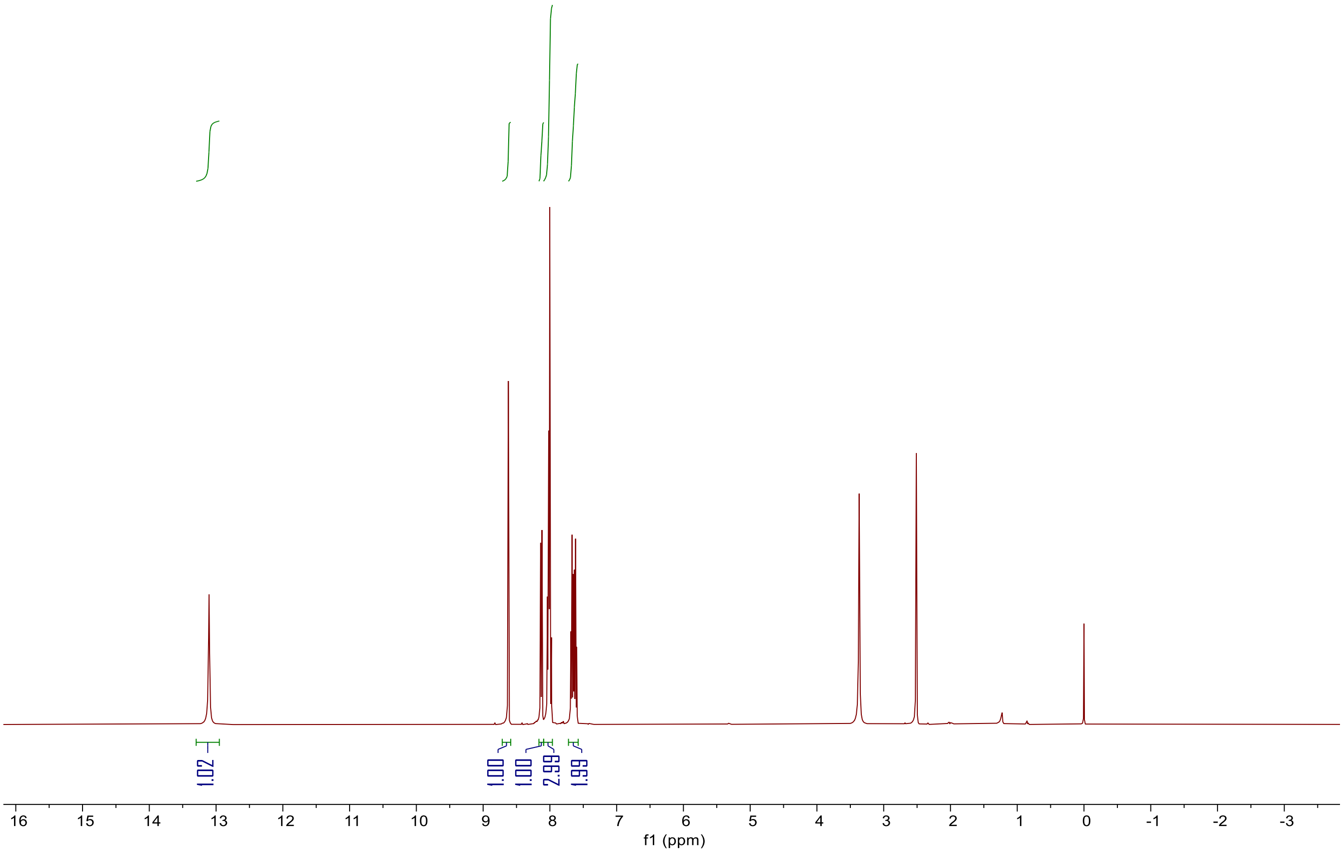

Supplementary Figure 51. ^{1}H NMR spectrum of 2-naphthoic acid in d-DMSO.

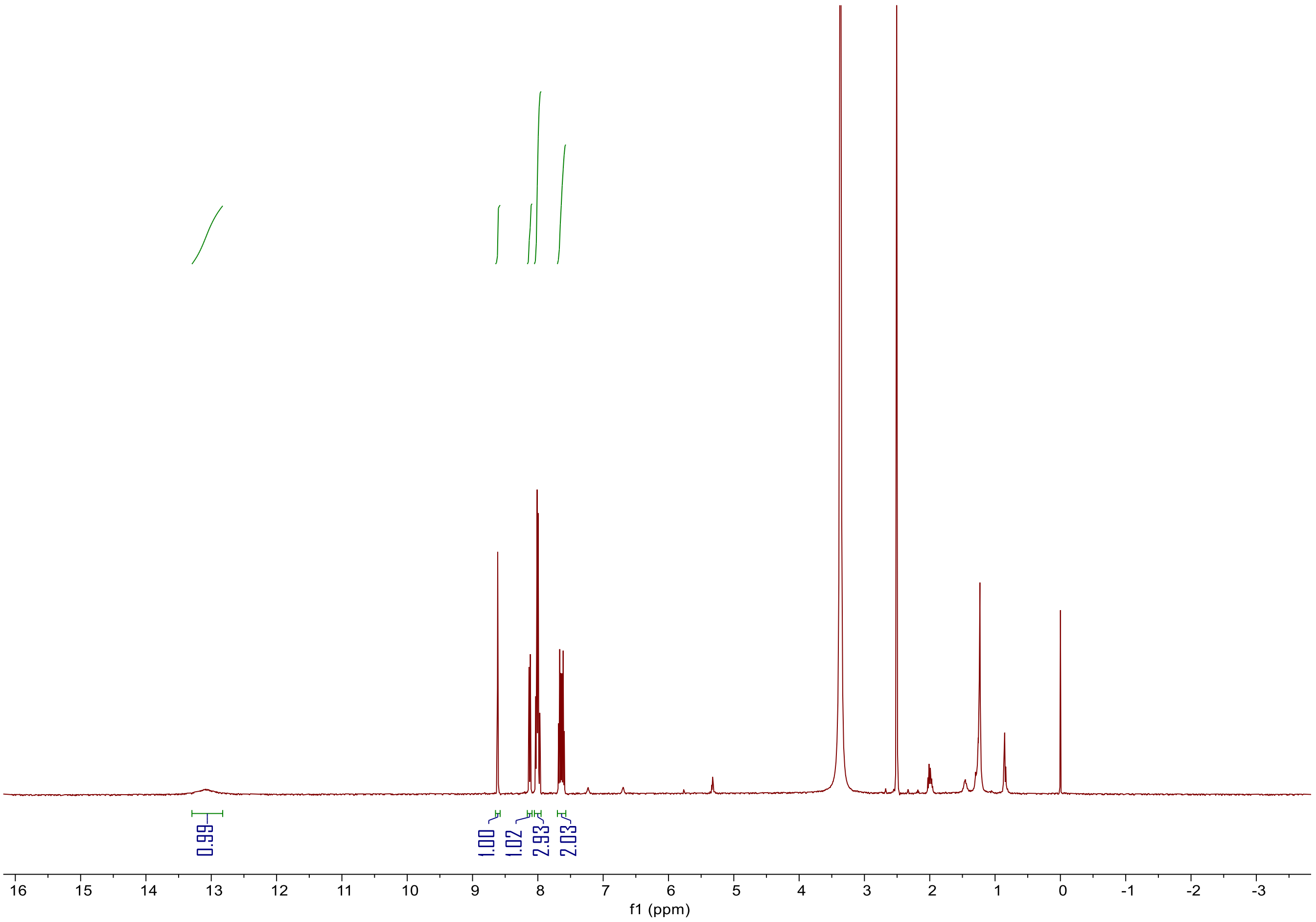

Supplementary Figure 52. ^{1}H NMR spectrum of the crude product of L-Histidine and 2-naphthoyl chloride in d-DMSO.

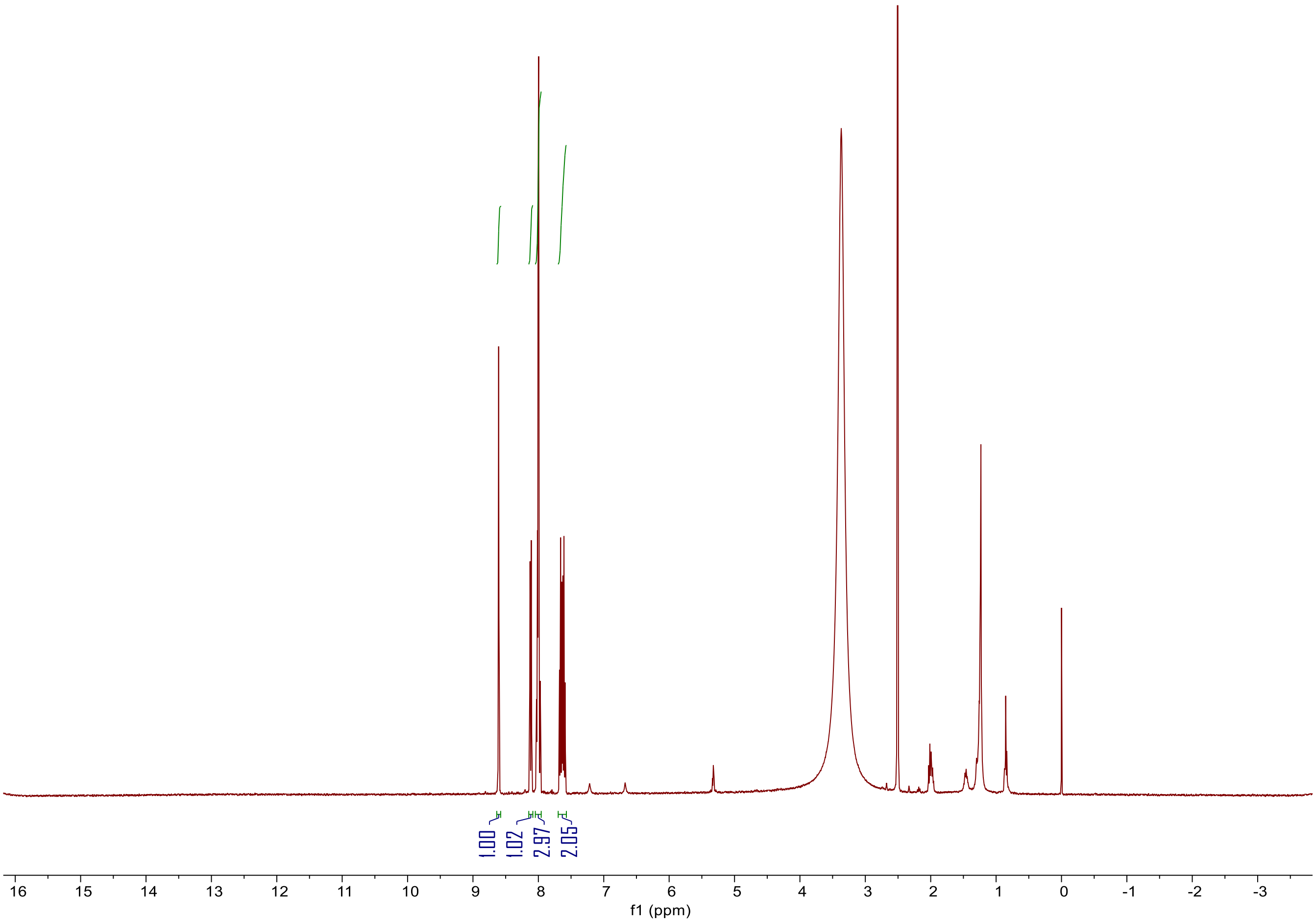

Supplementary Figure 53. ^{1}H NMR spectrum of the crude product of D-Histidine and 2-naphthoyl chloride in d-DMSO.

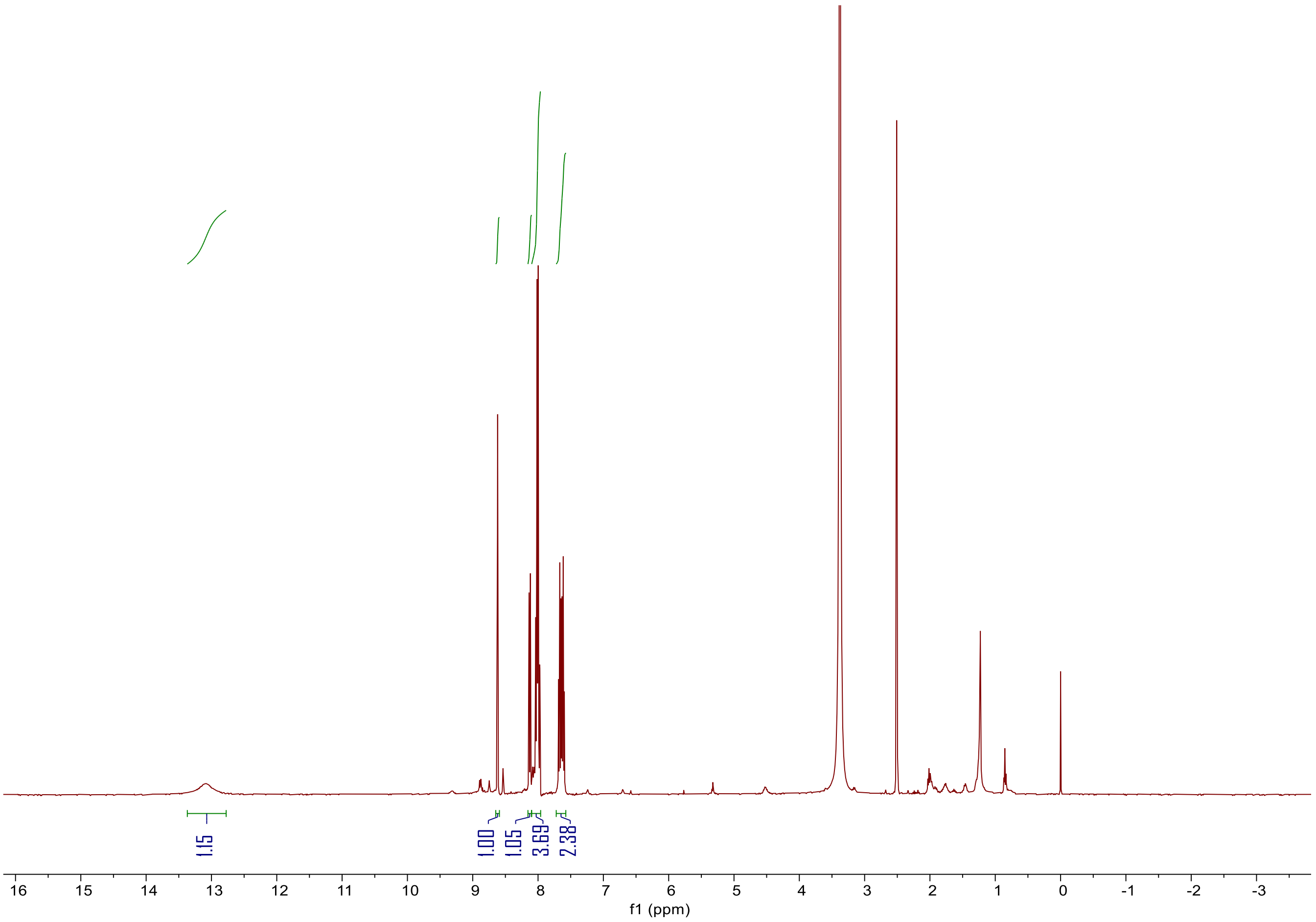

Supplementary Figure 54. ^{1}H NMR spectrum of the crude product of L-Arginine and 2-naphthoyl chloride in d-DMSO.

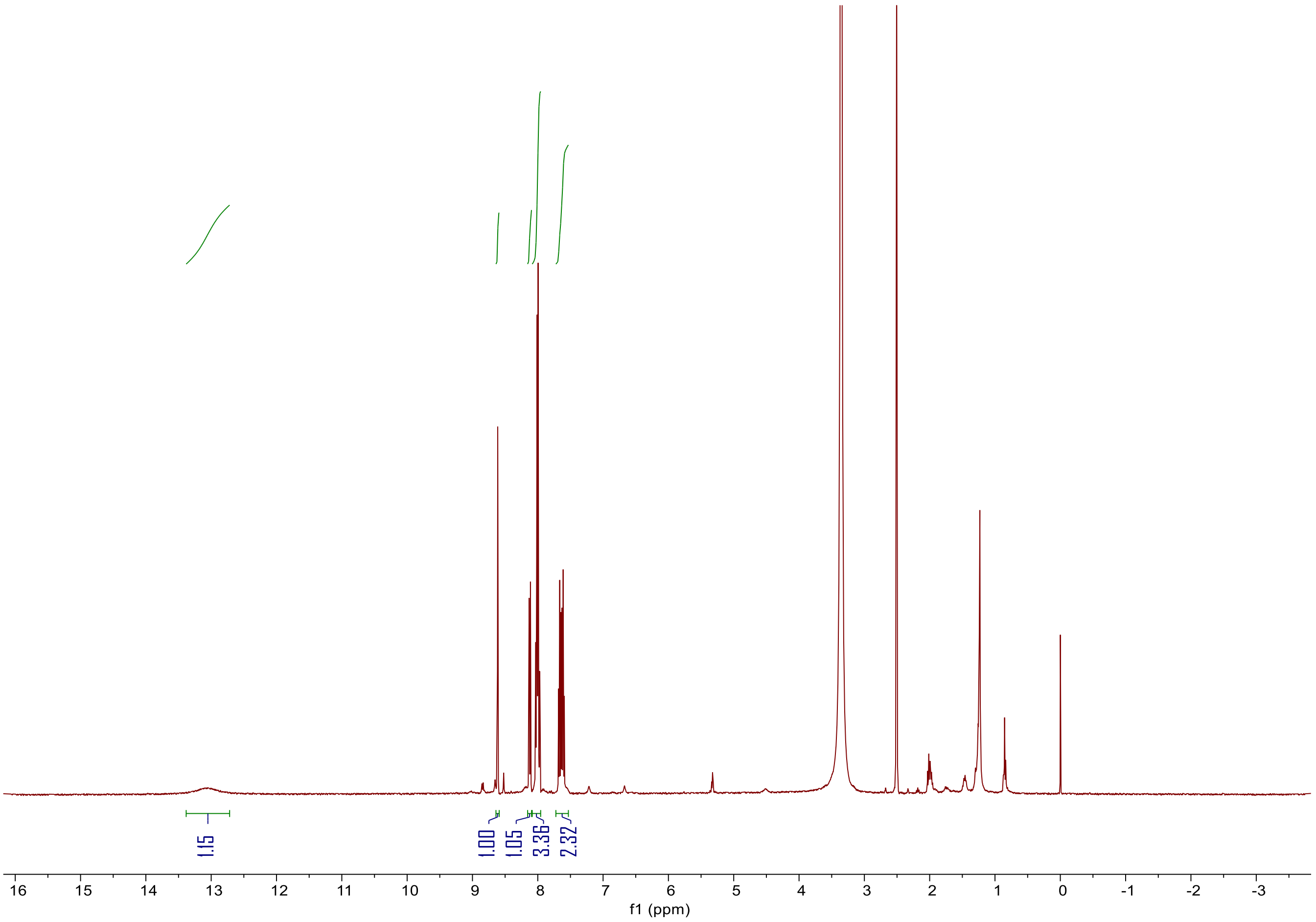

Supplementary Figure 55. ^{1}H NMR spectrum of the crude product of D-Arginine and 2-naphthoyl chloride in d-DMSO.

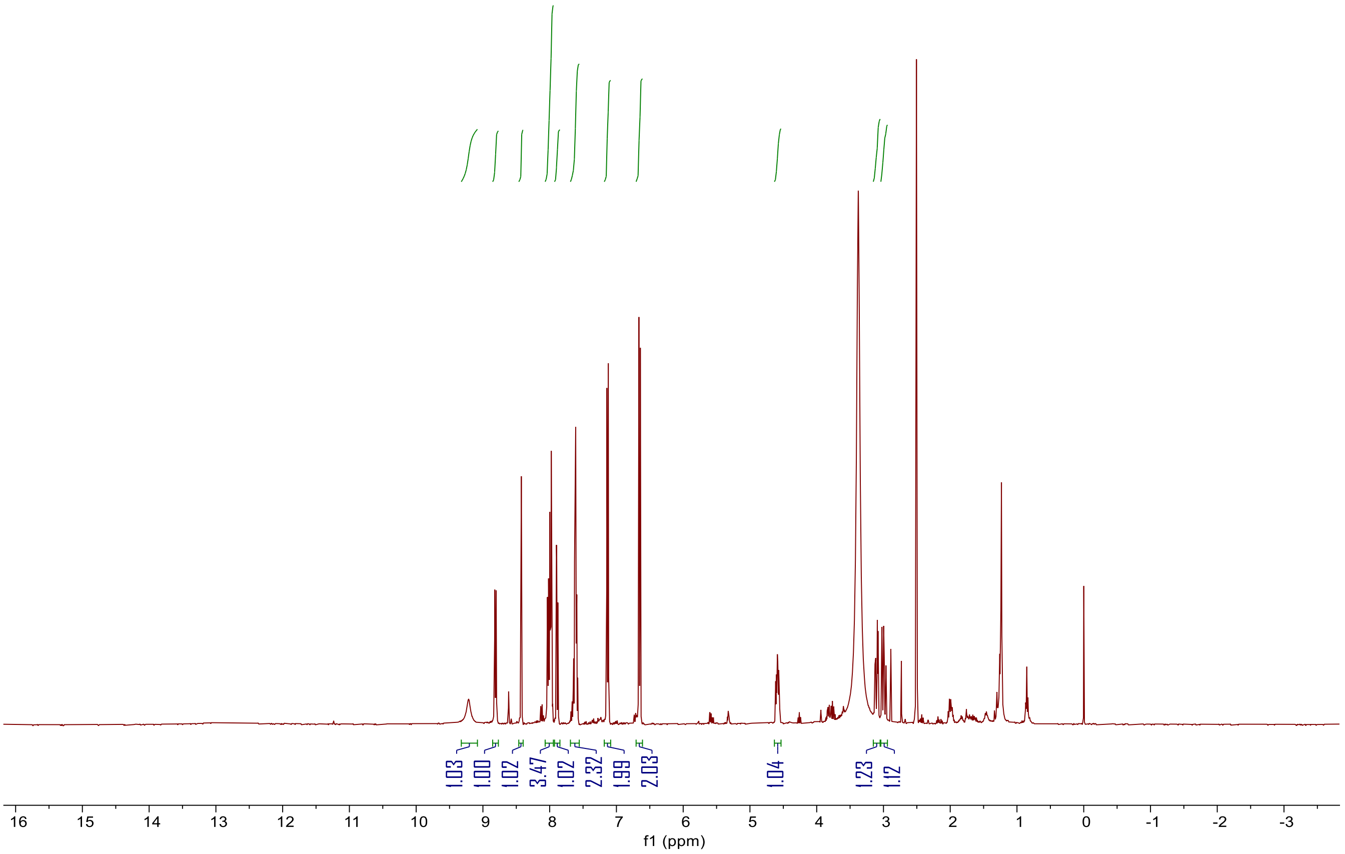

Supplementary Figure 56. ^{1}H NMR spectrum of the crude product of L-Tyrosine and 2-naphthoyl chloride in d-DMSO.

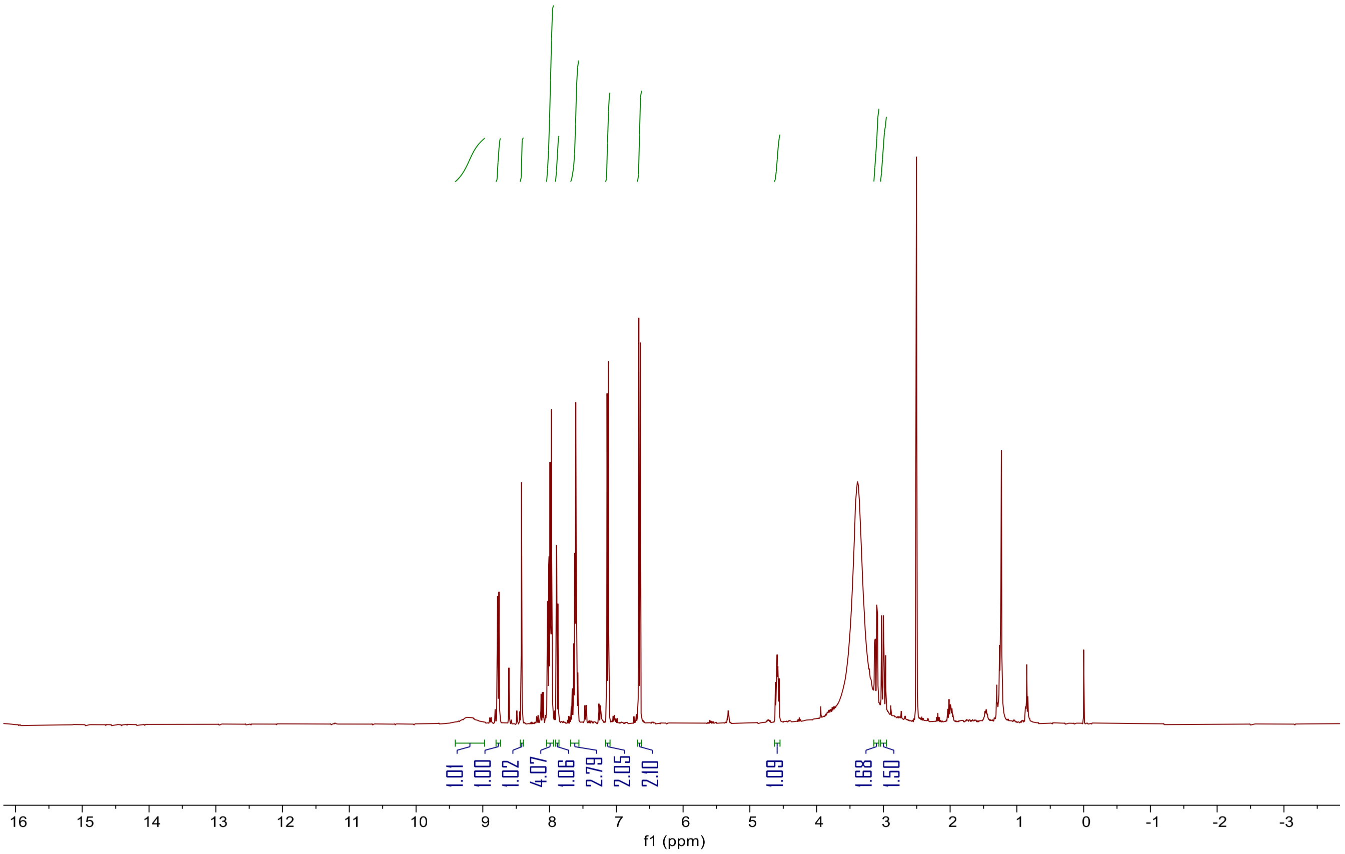

Supplementary Figure 57. ^{1}H NMR spectrum of the crude product of D-Tyrosine and 2-naphthoyl chloride in d-DMSO.

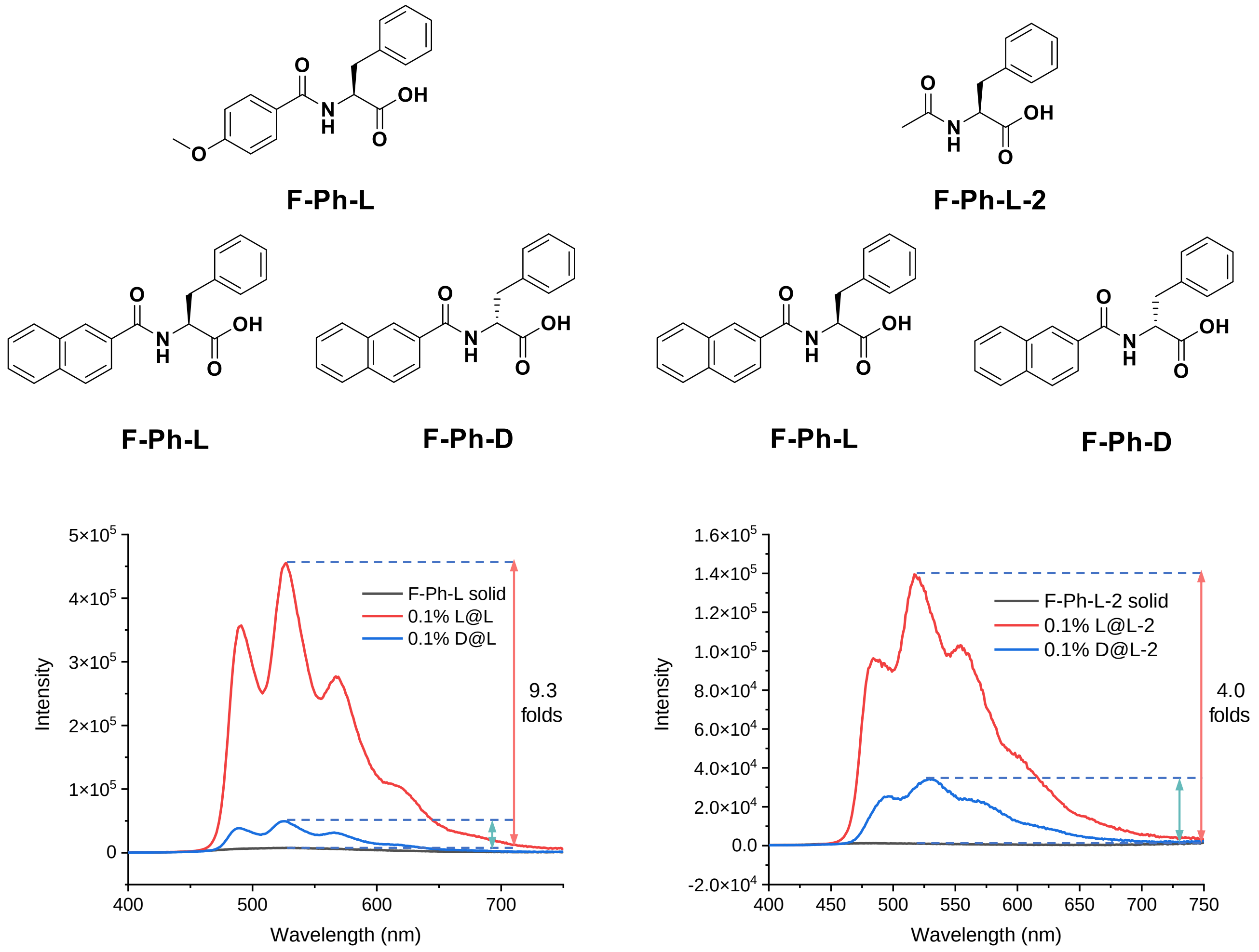

Supplementary Figure 58. Molecular structures of the hosts and guests and delayed emission (DE, Δt = 5 ms) spectra of doped samples (w/w = 0.1%) at 298 K (λ_{ex} = 247 nm).

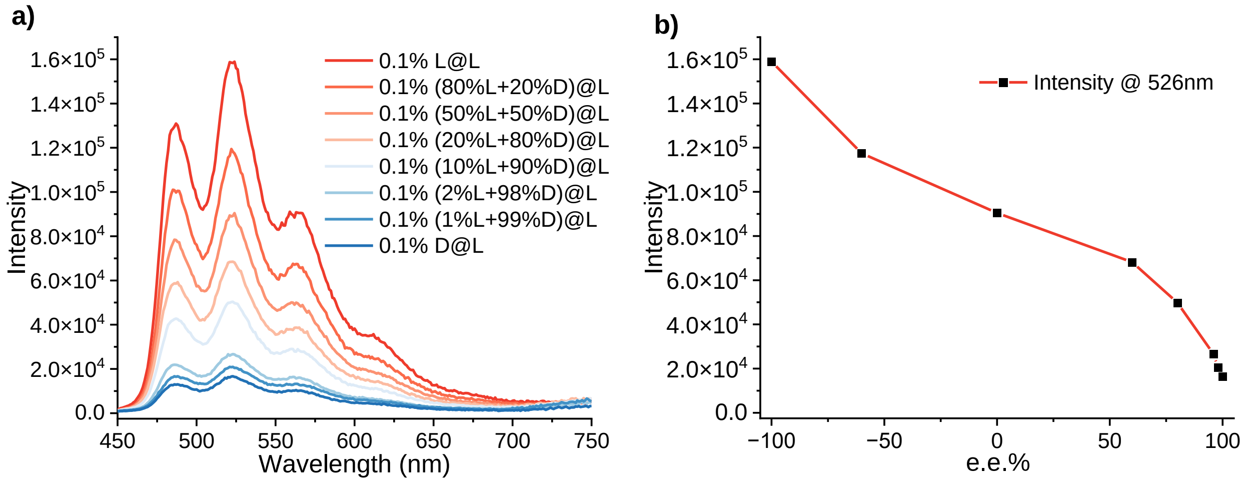

Supplementary Figure 59. a) The RTP emission intensity at 526 nm and b) correlation between the enantiomer excess (e.e.) values, illustrating the relationship between e.e. values and the efficiency of chiral recognition.

Nuclear Magnetic Resonance (NMR) and High-Resolution Mass (HRM) Spectra

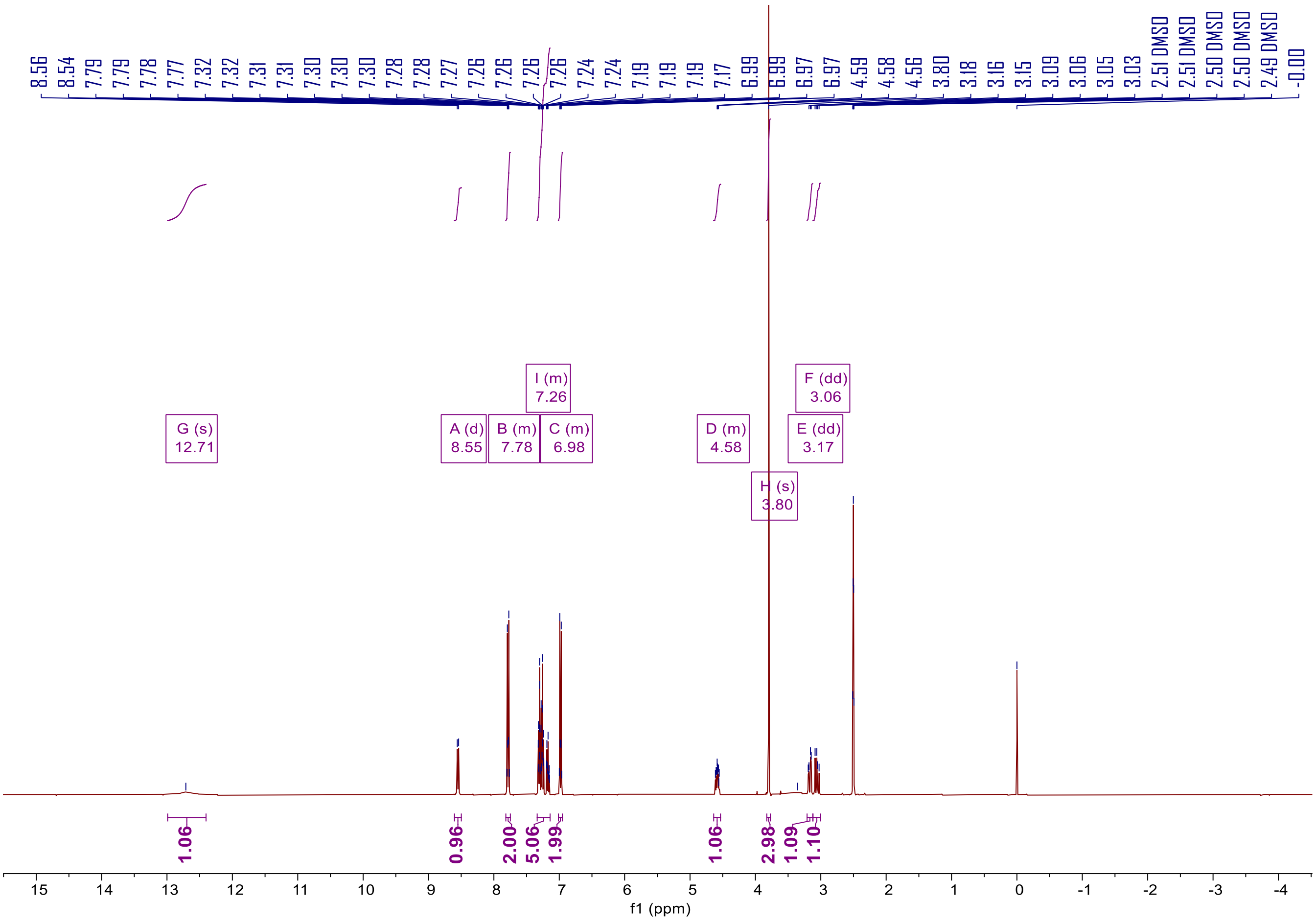

Supplementary Figure 60. ^{1}H NMR spectrum of **F-Ph-DL** in d-DMSO.

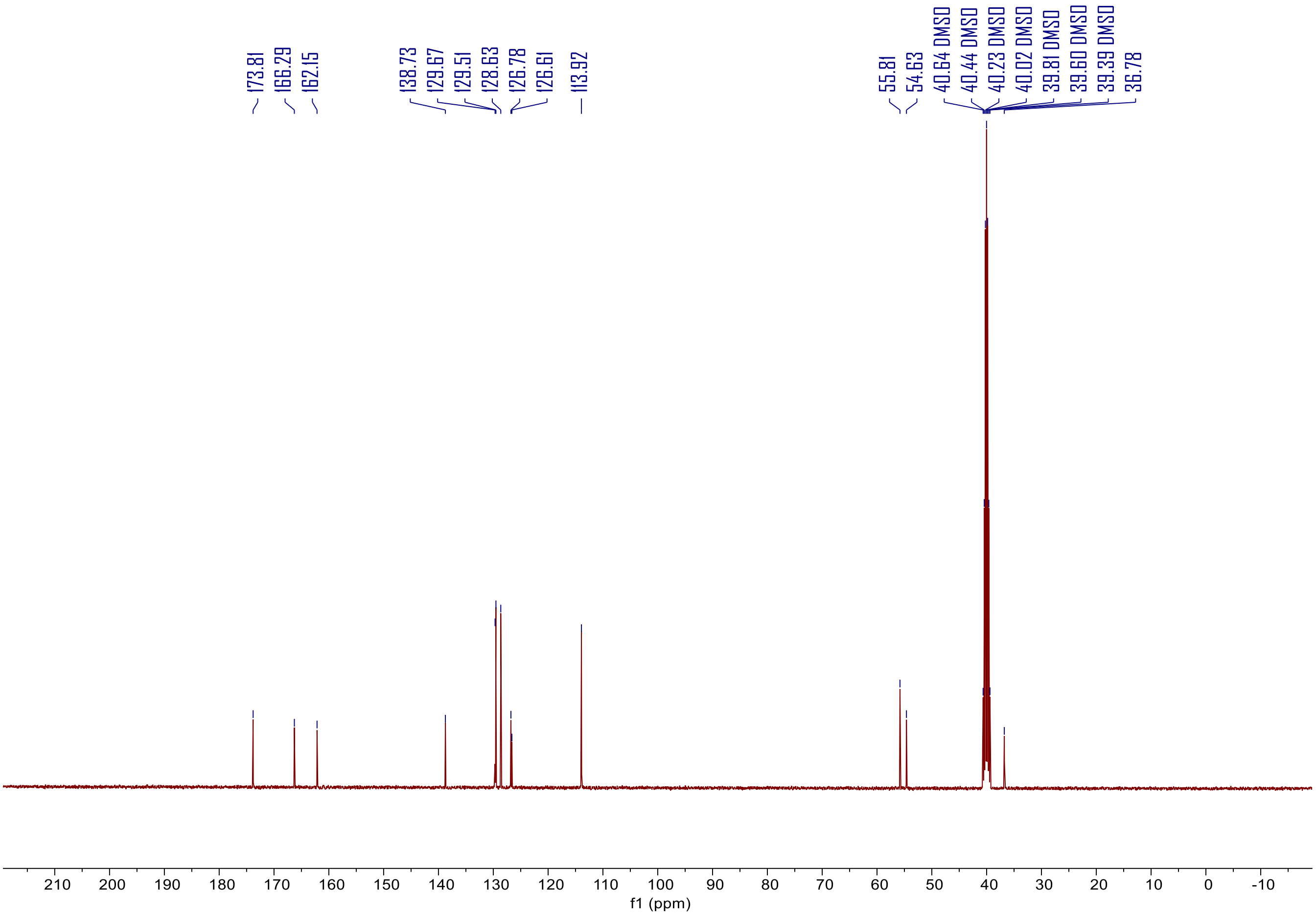

Supplementary Figure 61. ^{13}C NMR spectrum of **F-Ph-DL** in d-DMSO.

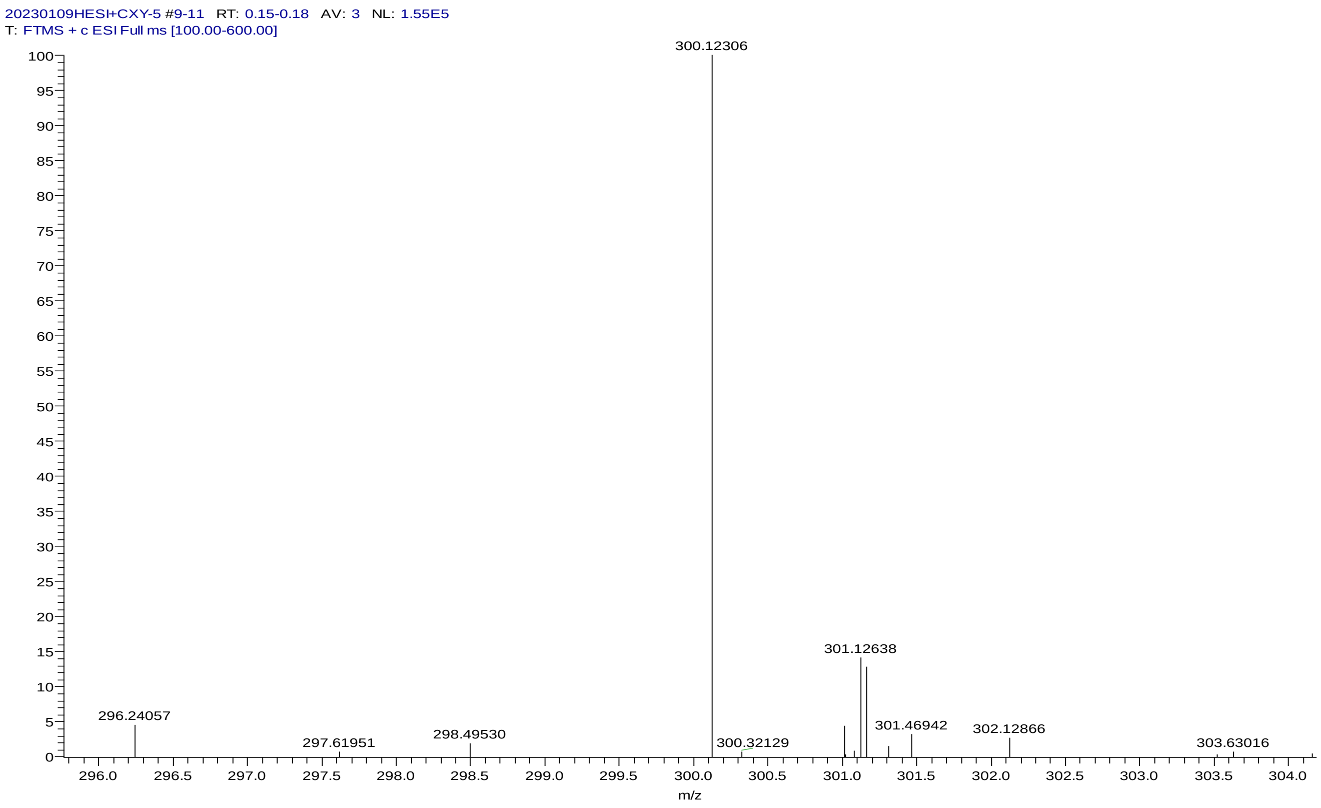

Supplementary Figure 62. EI mass spectrum of **F-Ph-DL**.

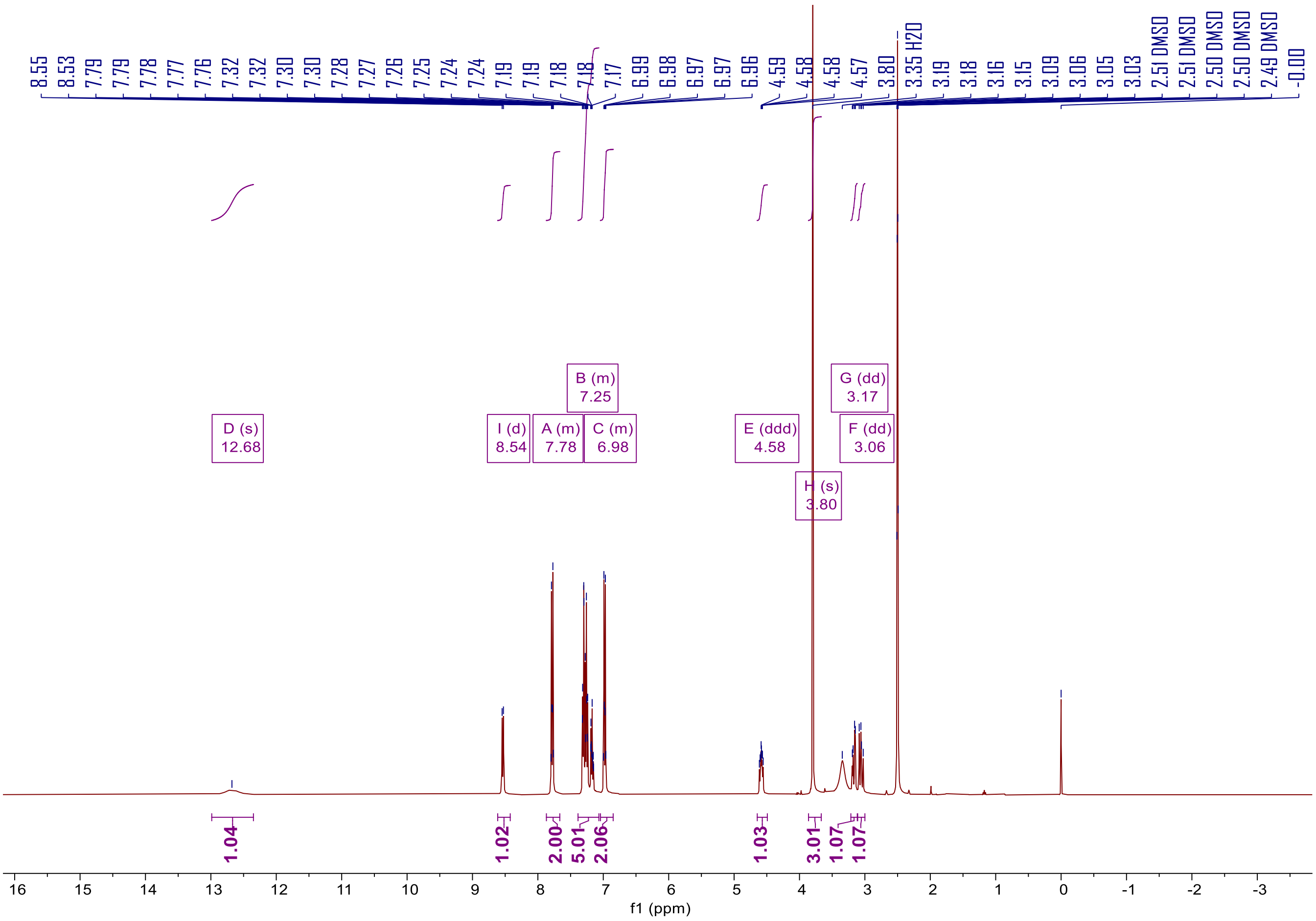

Supplementary Figure 63. ^1H NMR spectrum of **F-Ph-L** in d-DMSO.

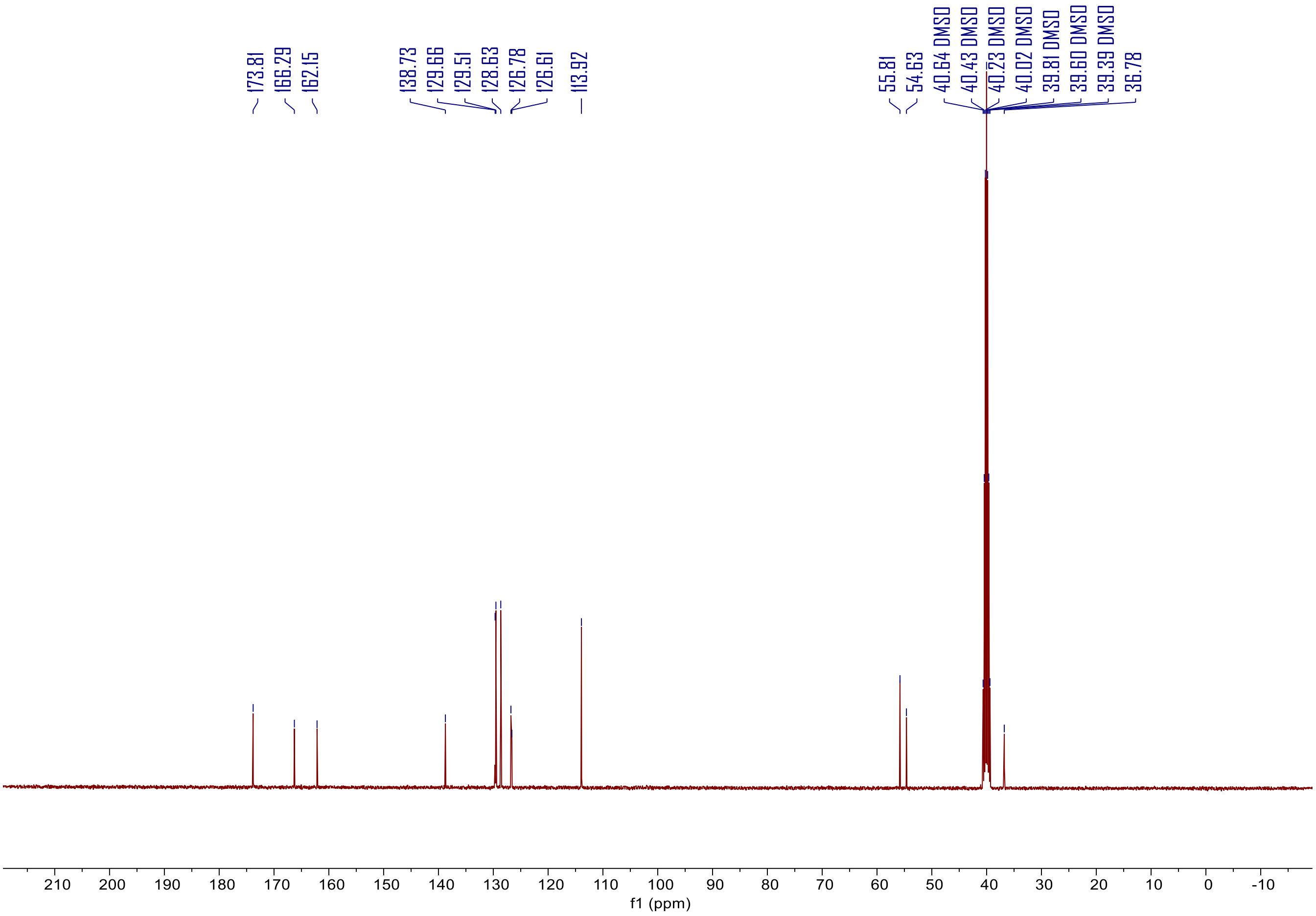

Supplementary Figure 64. ^{13}C NMR spectrum of **F-Ph-L** in d-DMSO.

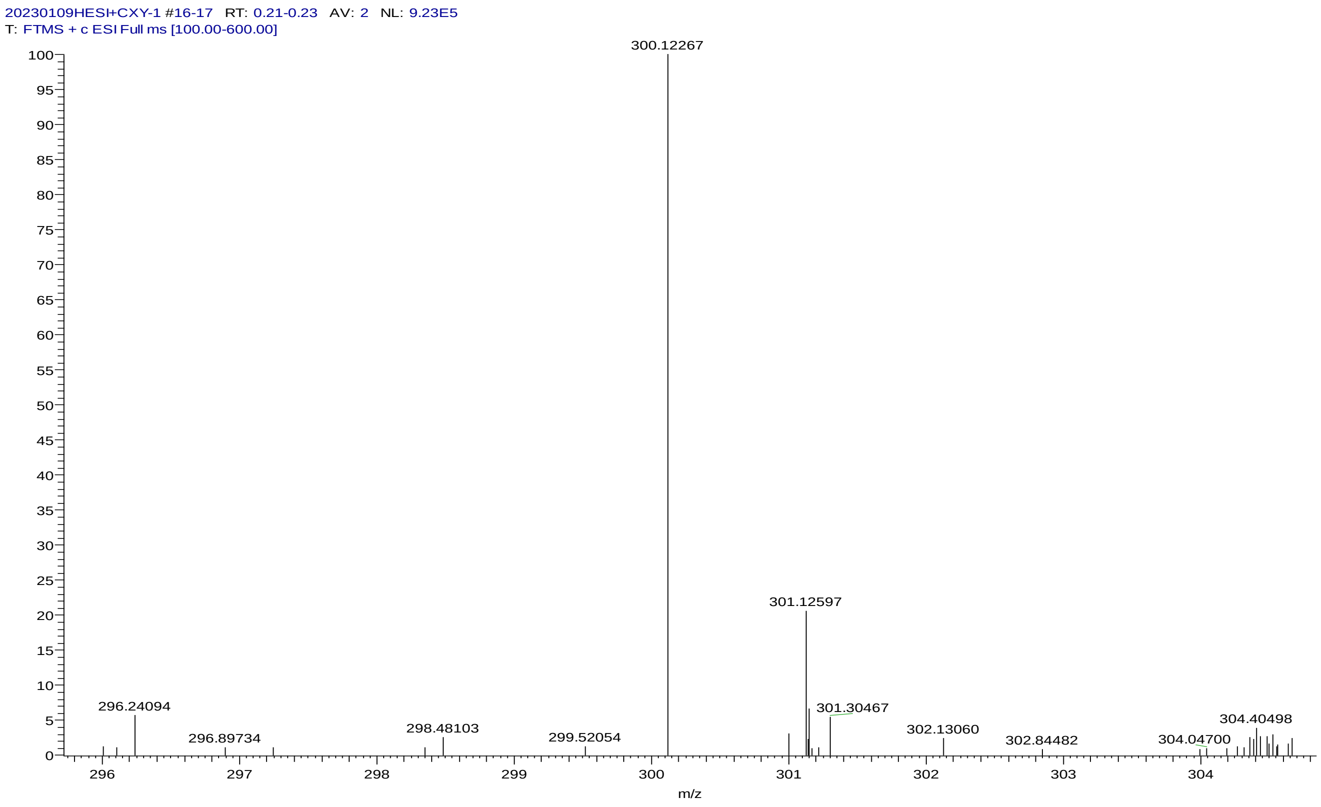

Supplementary Figure 65. EI mass spectrum of **F-Ph-L**.

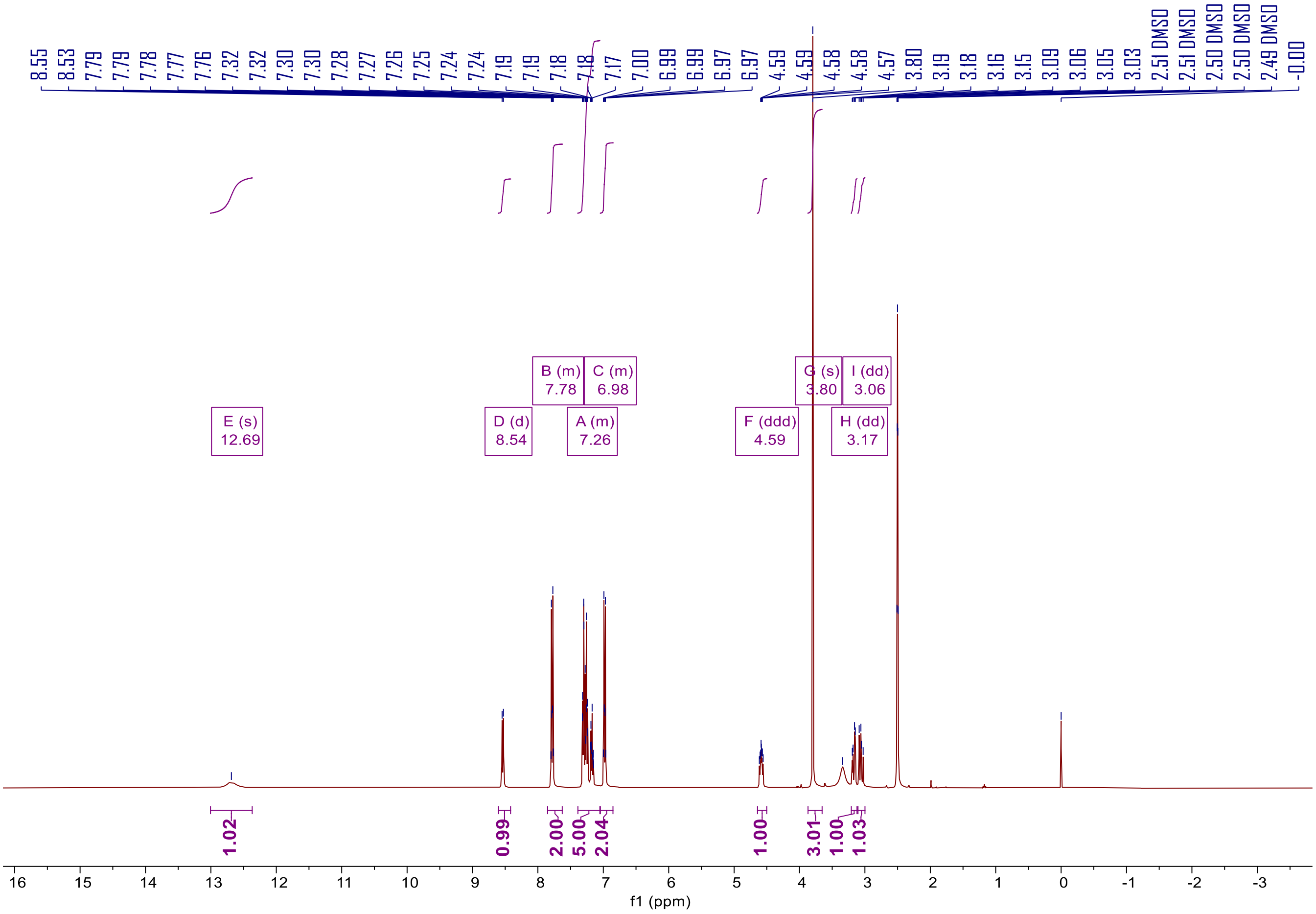

Supplementary Figure 66. ^{1}H NMR spectrum of **F-Ph-D** in d-DMSO.

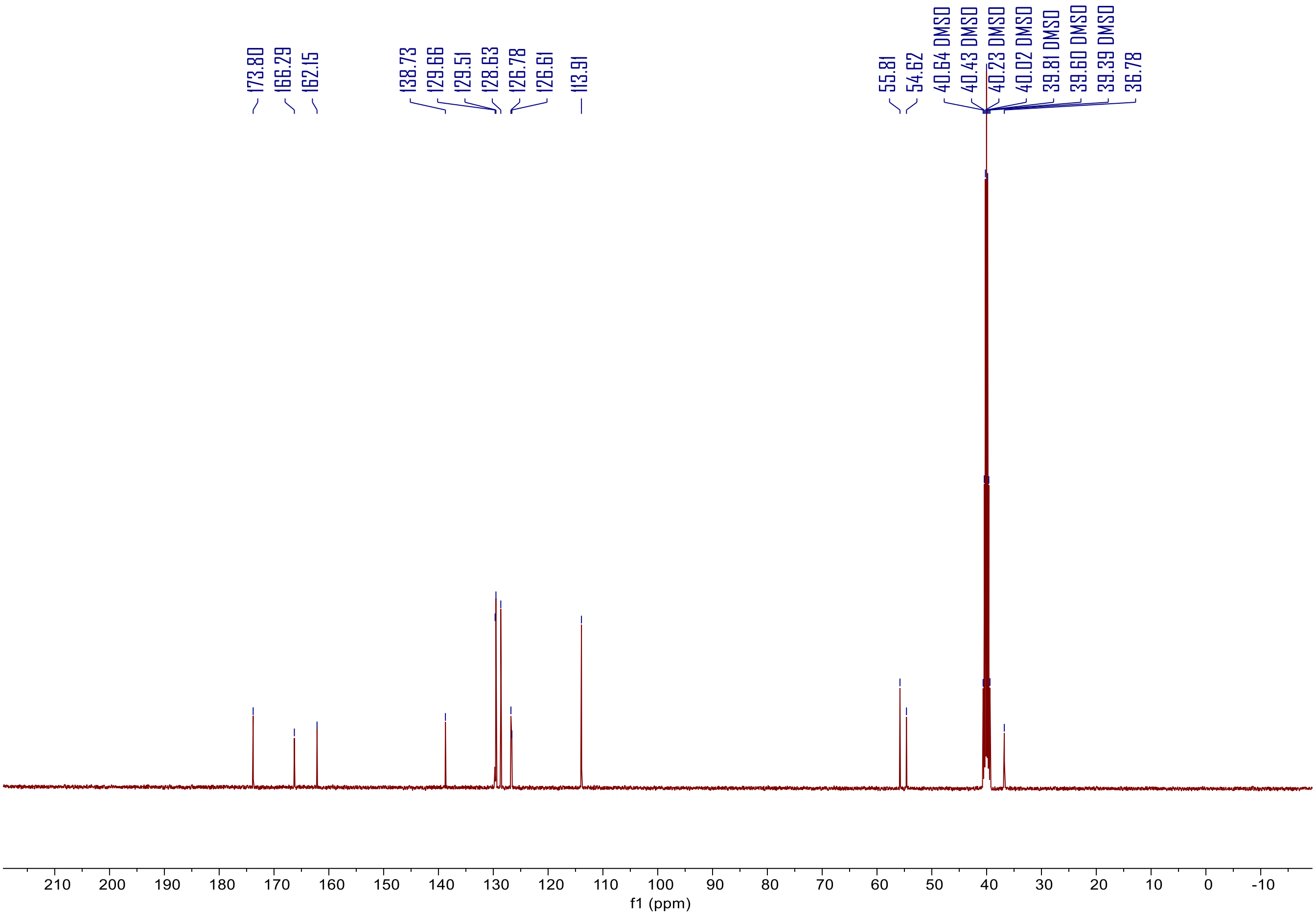

Supplementary Figure 67. ^{13}C NMR spectrum of **F-Ph-D** in d-DMSO.

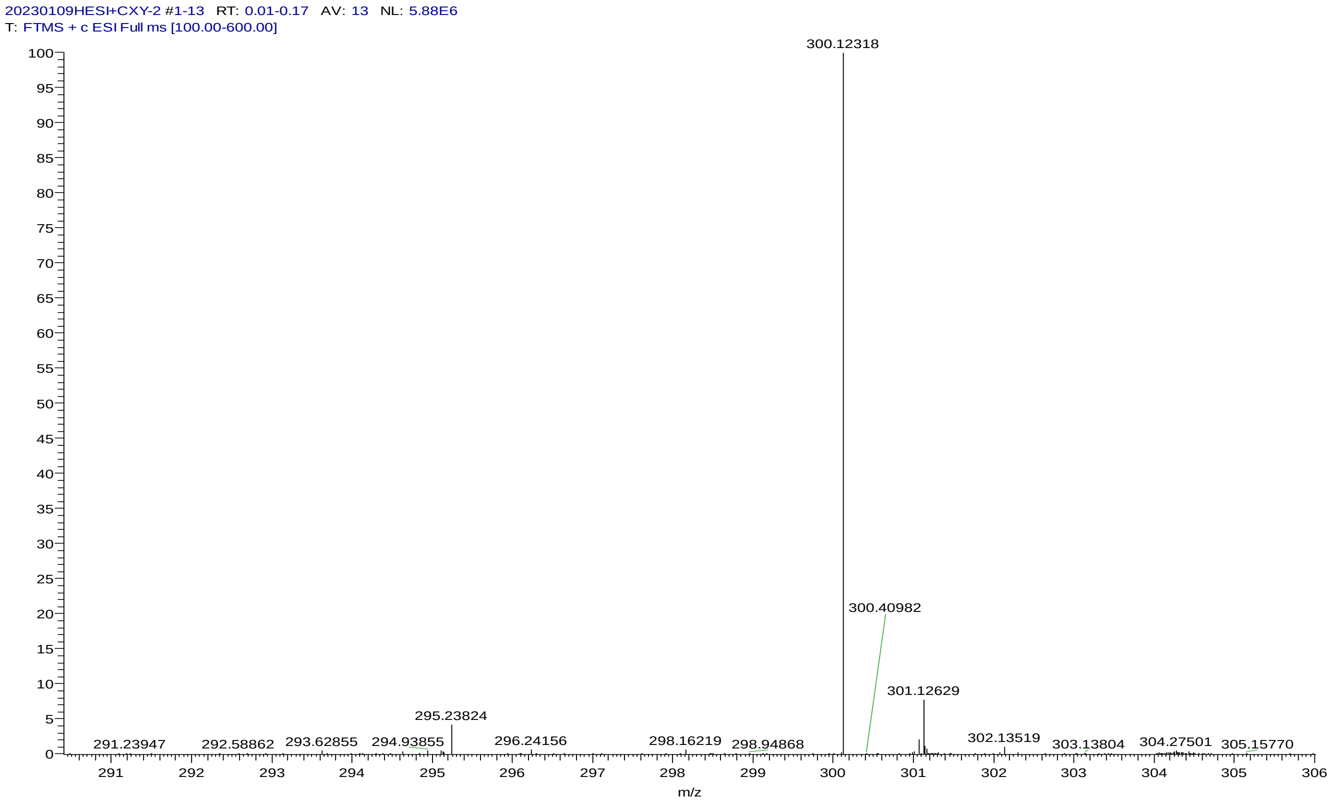

Supplementary Figure 68. EI mass spectrum of **F-Ph-D**.

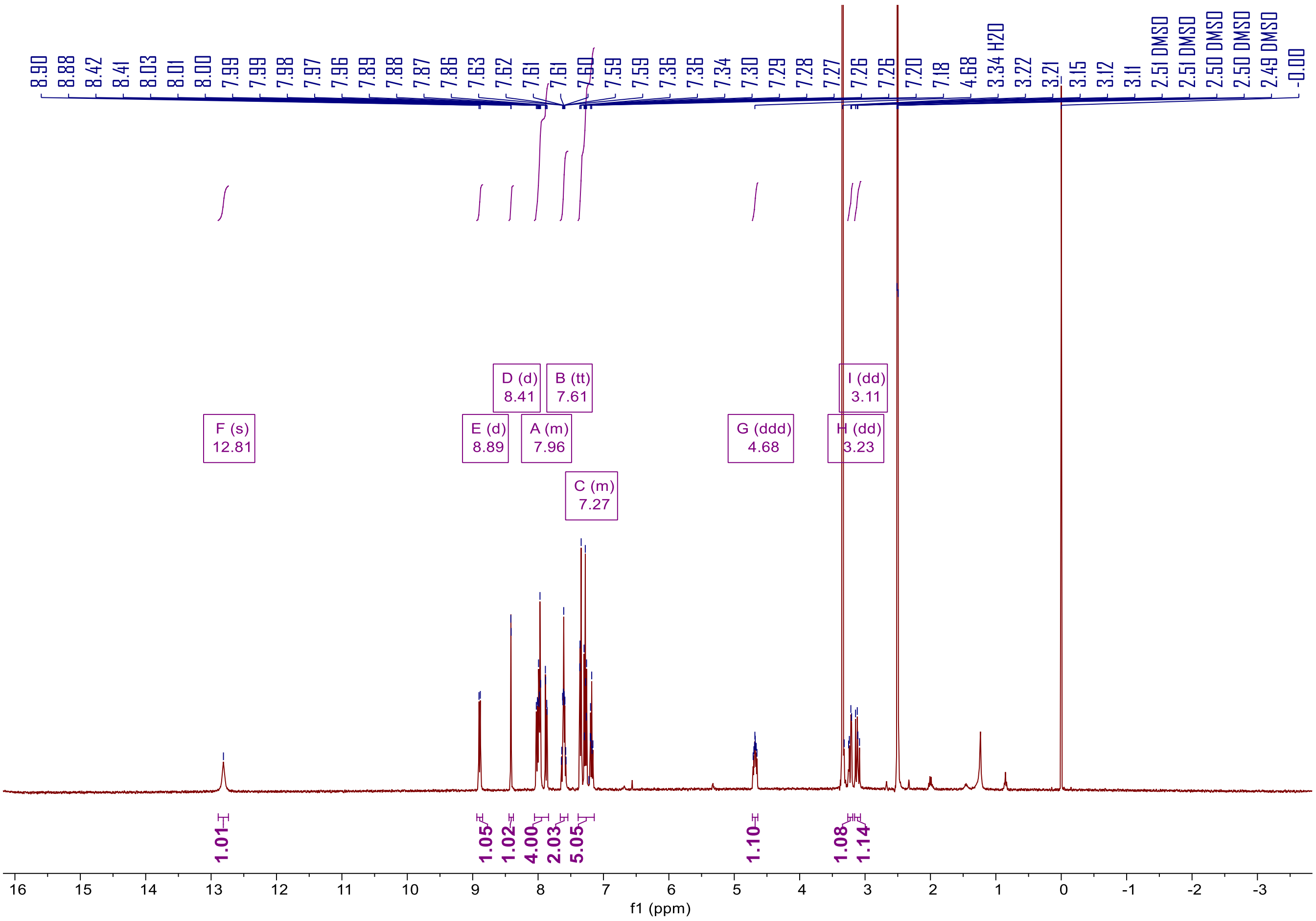

Supplementary Figure 69. ^{1}H NMR spectrum of **F-Na-L** in d-DMSO.

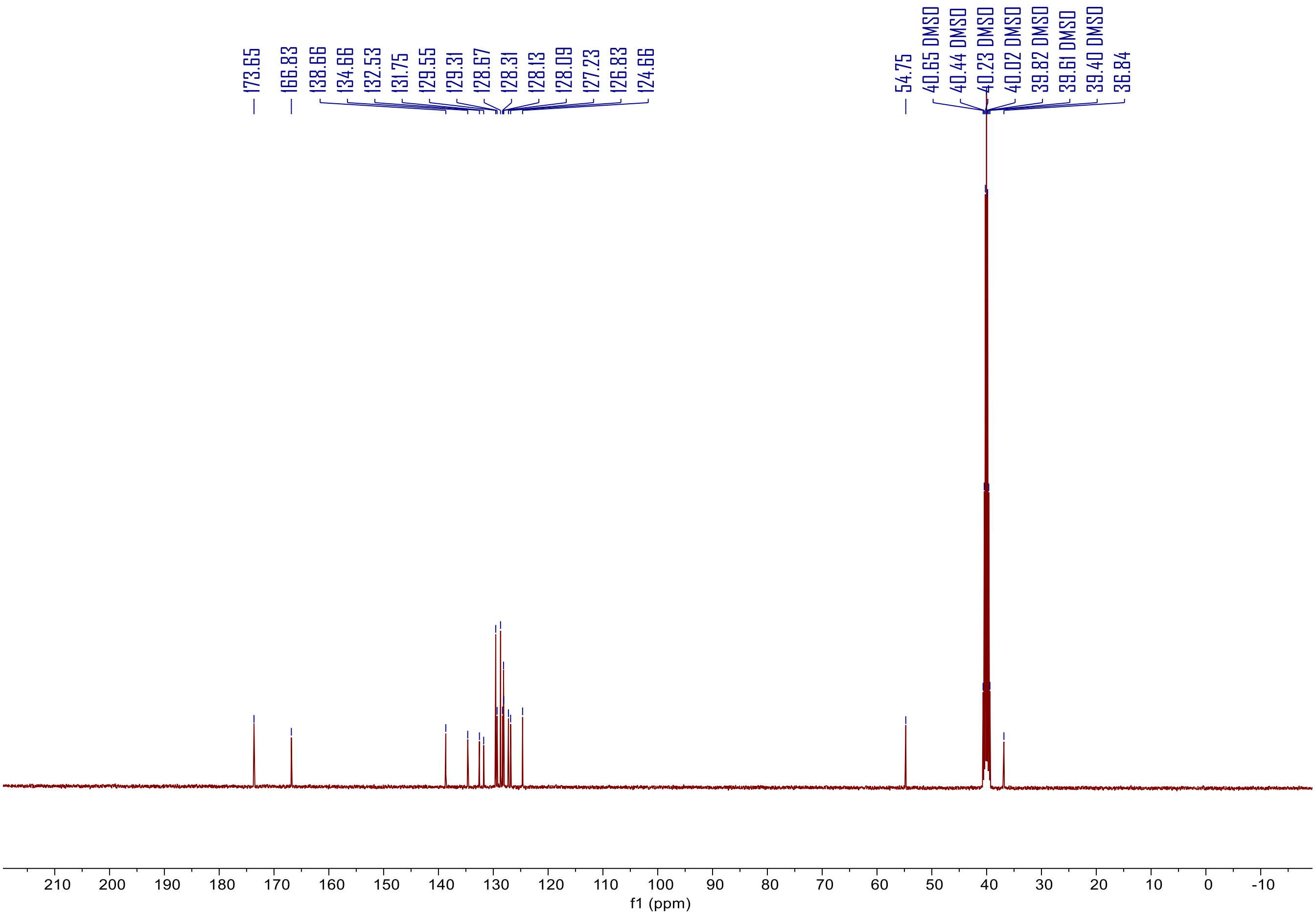

Supplementary Figure 70. ^{13}C NMR spectrum of **F-Na-L** in d-DMSO.

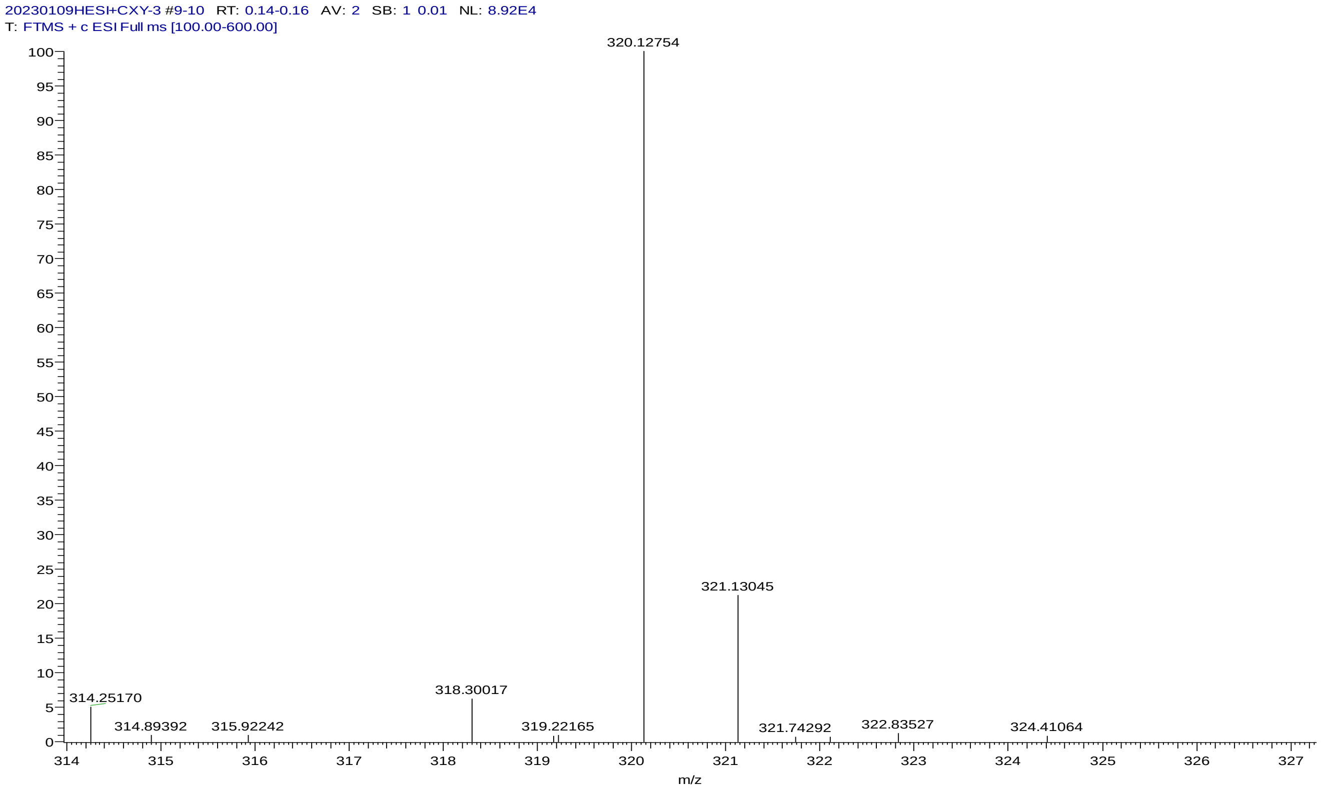

Supplementary Figure 71. EI mass spectrum of **F-Na-L**.

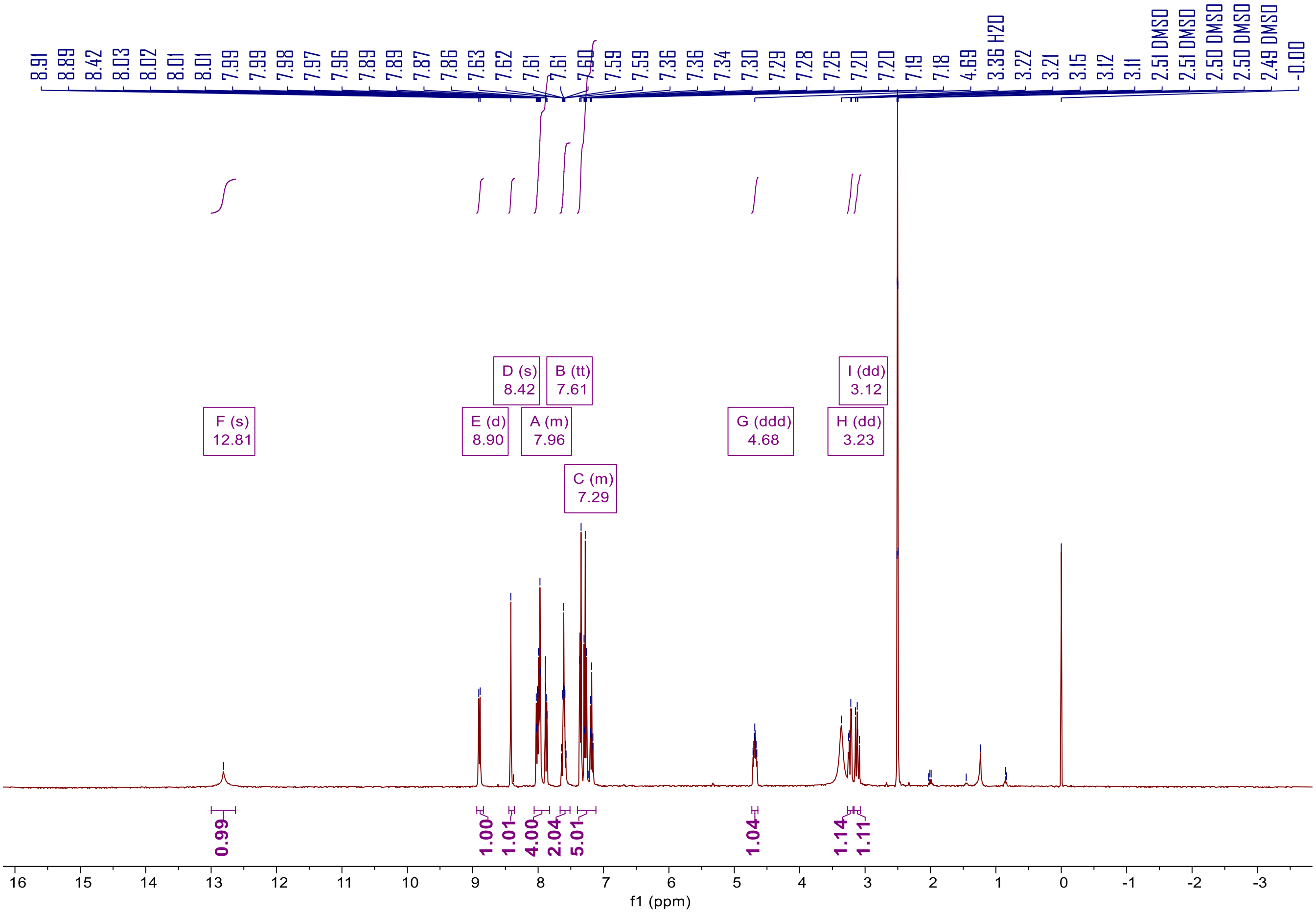

Supplementary Figure 72. ^{1}H NMR spectrum of **F-Na-D** in d-DMSO.

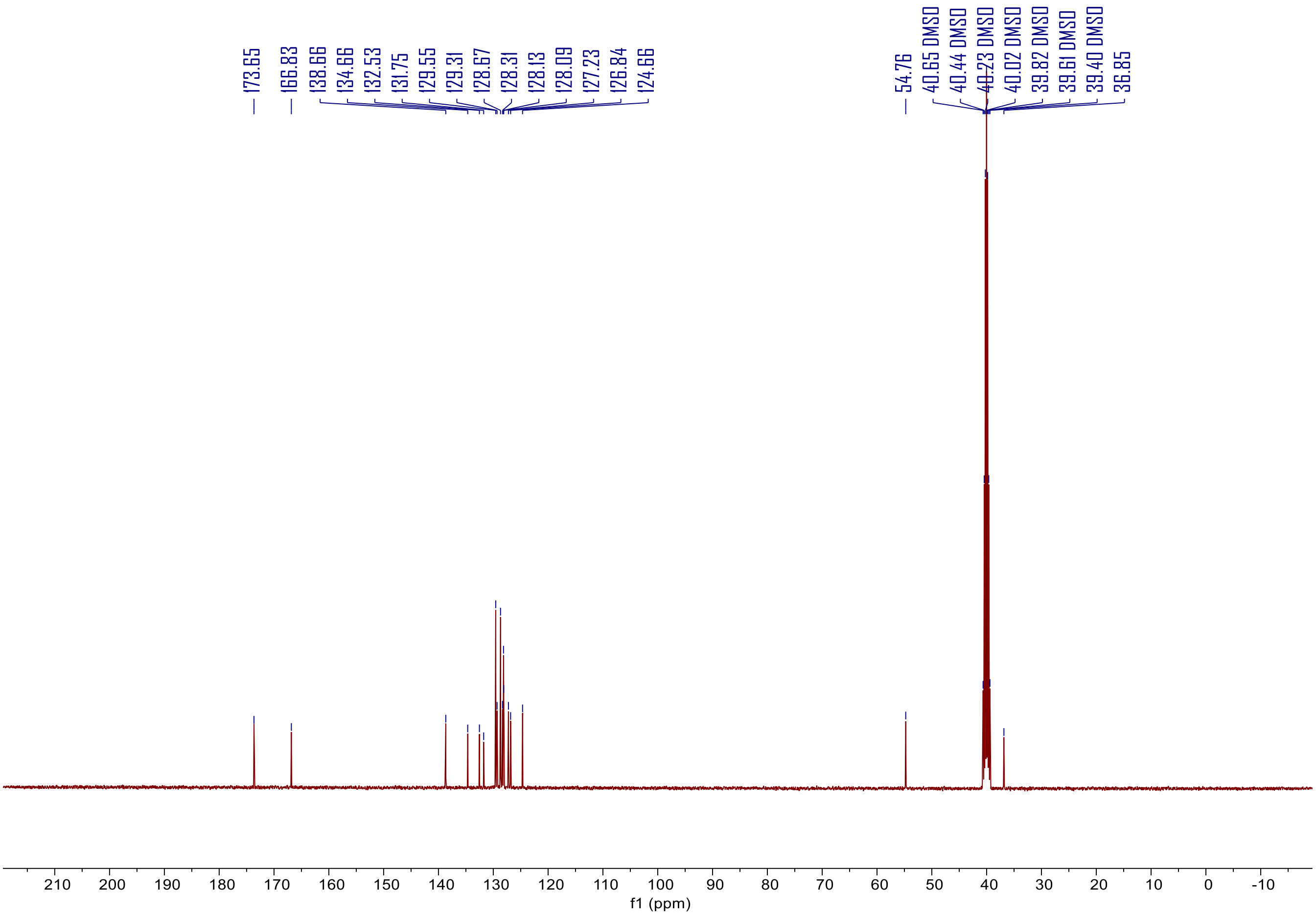

Supplementary Figure 73. ^{13}C NMR spectrum of **F-Na-D** in d-DMSO.

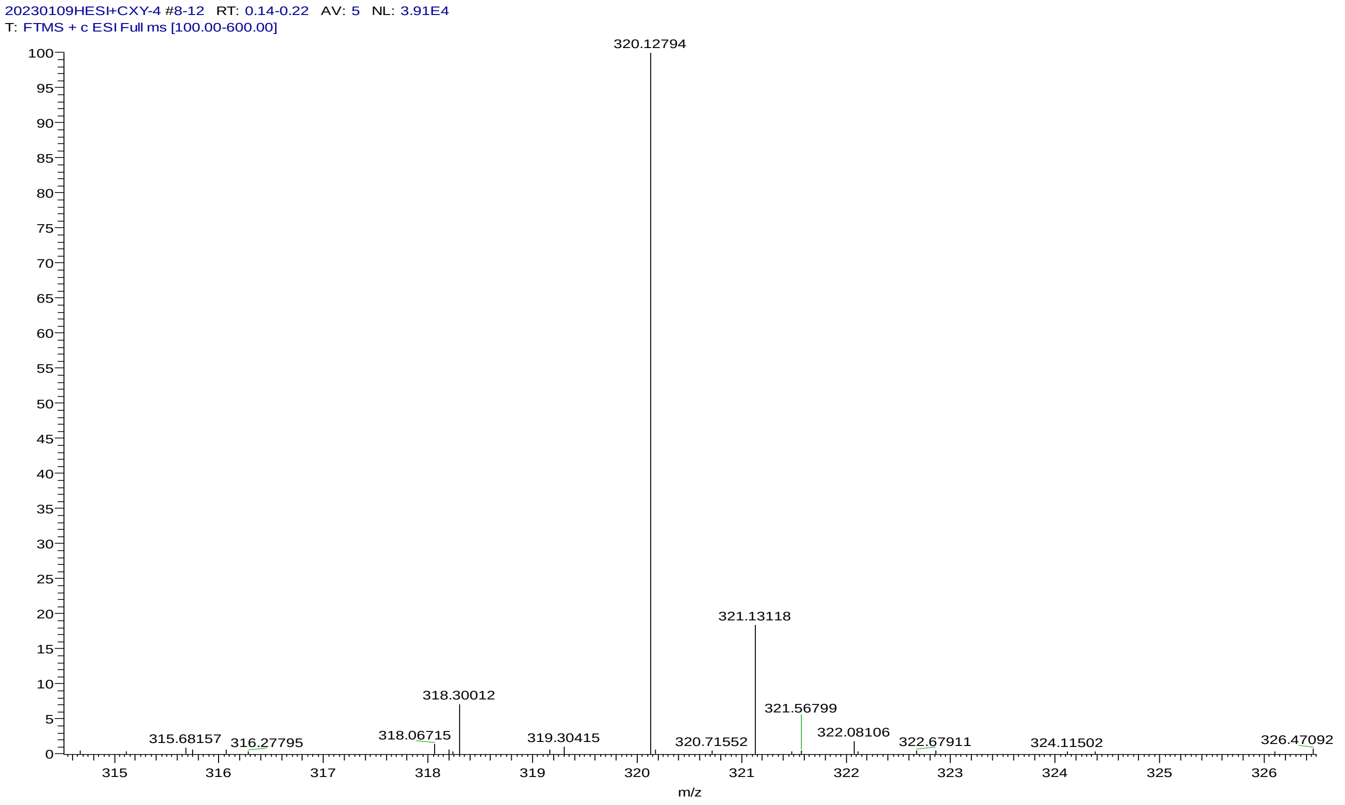

Supplementary Figure 74. EI mass spectrum of **F-Na-D**.

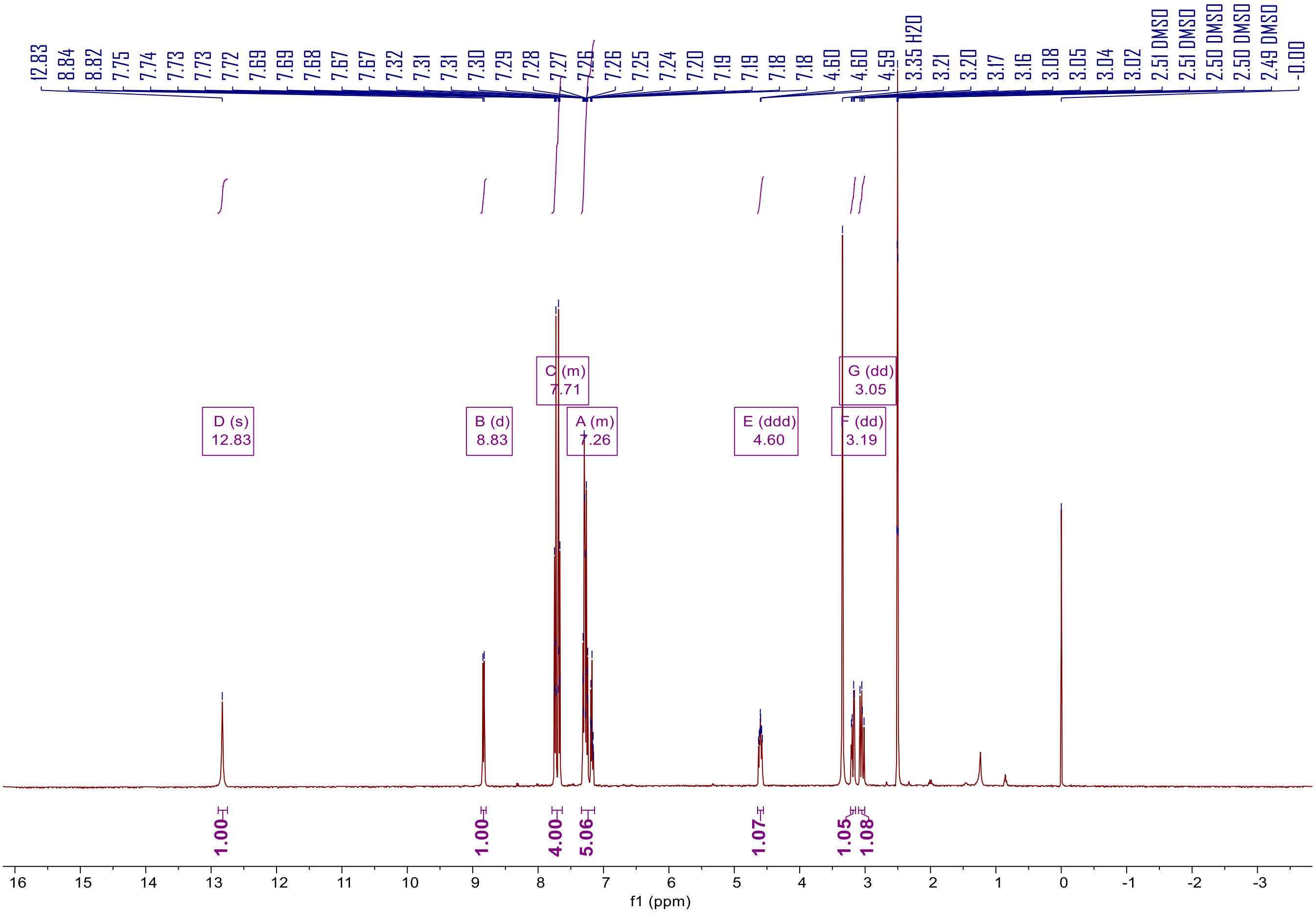

Supplementary Figure 75. ^{1}H NMR spectrum of **F-Ph-Br-L** in d-DMSO.

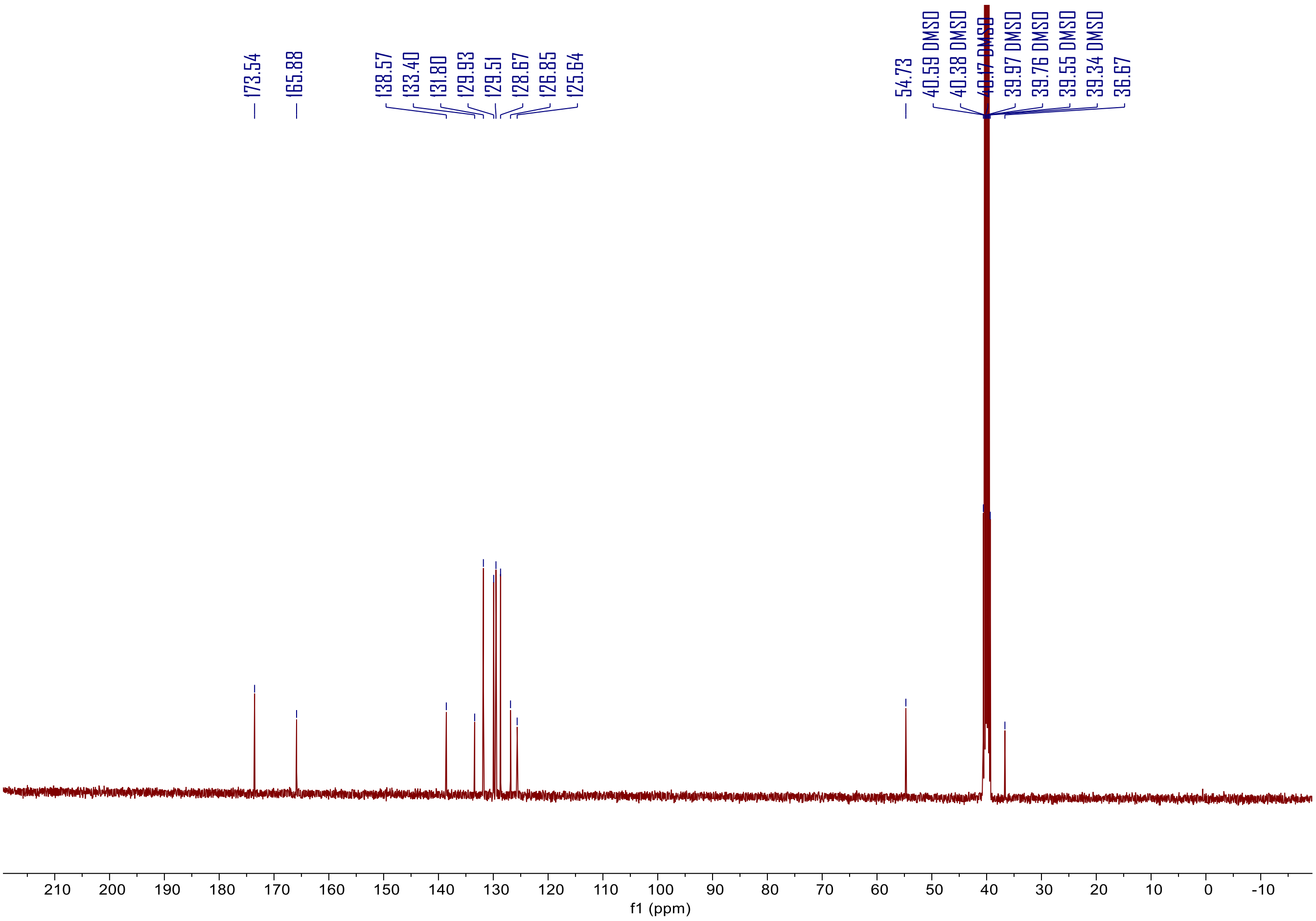

Supplementary Figure 76. ^{13}C NMR spectrum of **F-Ph-Br-L** in d-DMSO.

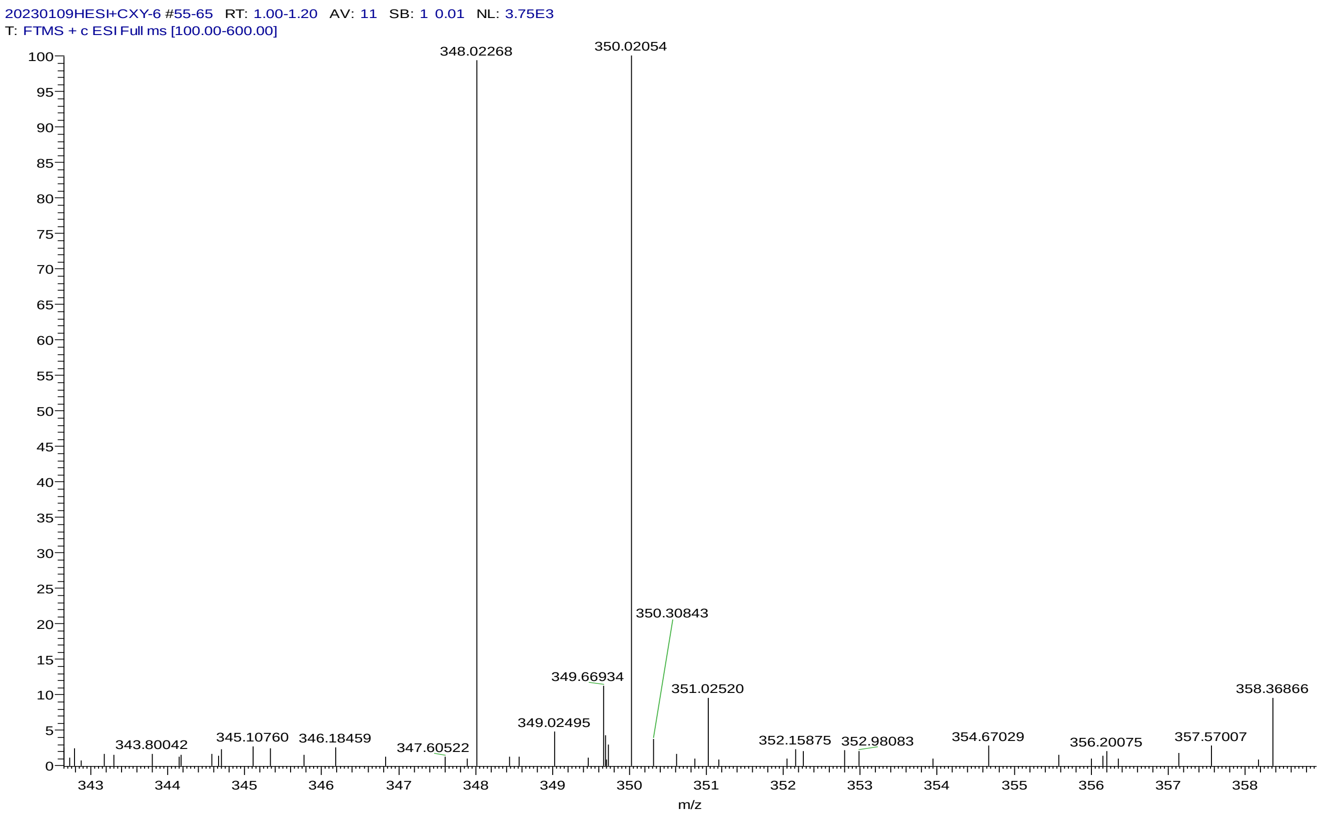

Supplementary Figure 77. EI mass spectrum of **F-Ph-Br-L**.

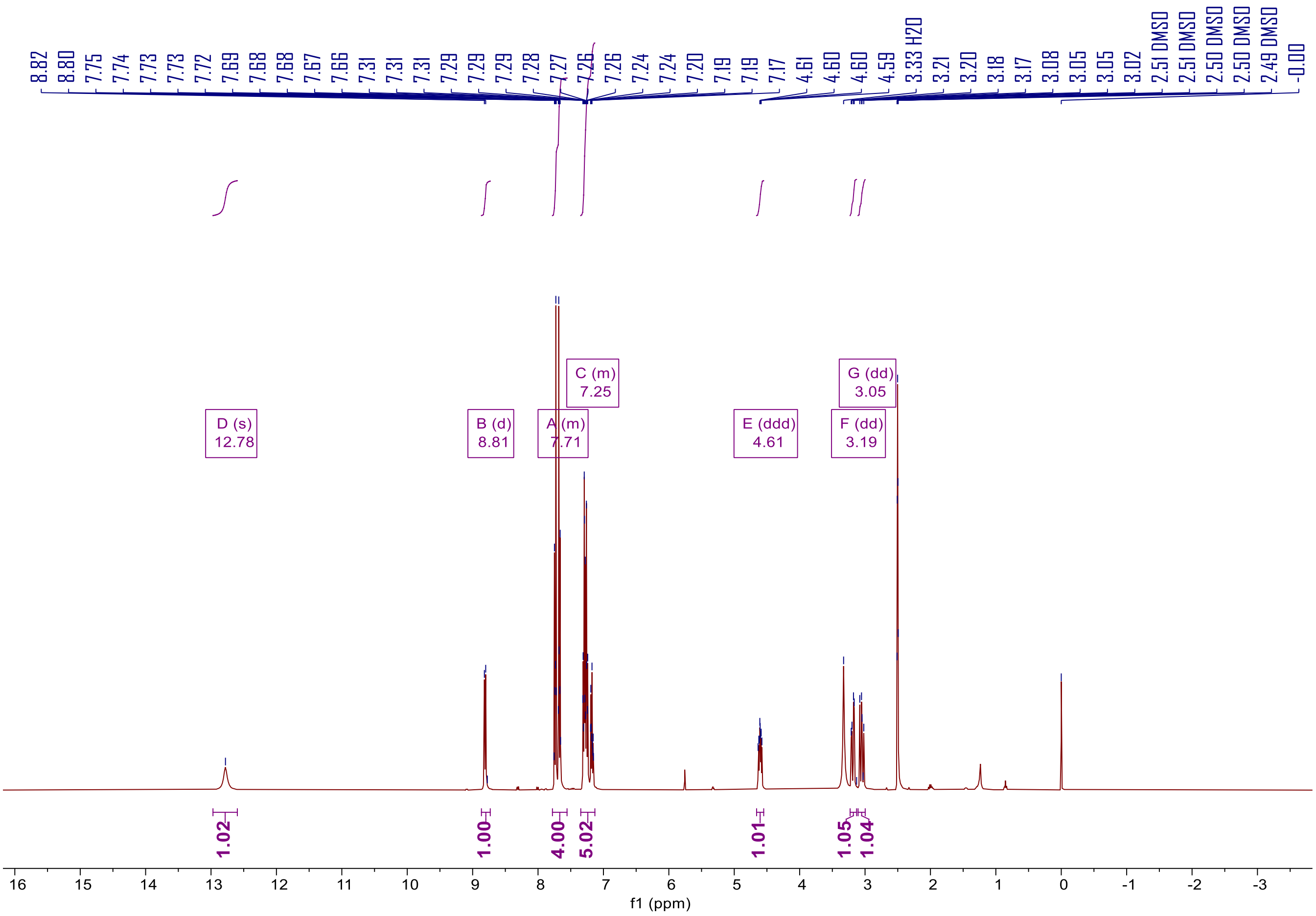

Supplementary Figure 78. ^{1}H NMR spectrum of **F-Ph-Br-D** in d-DMSO.

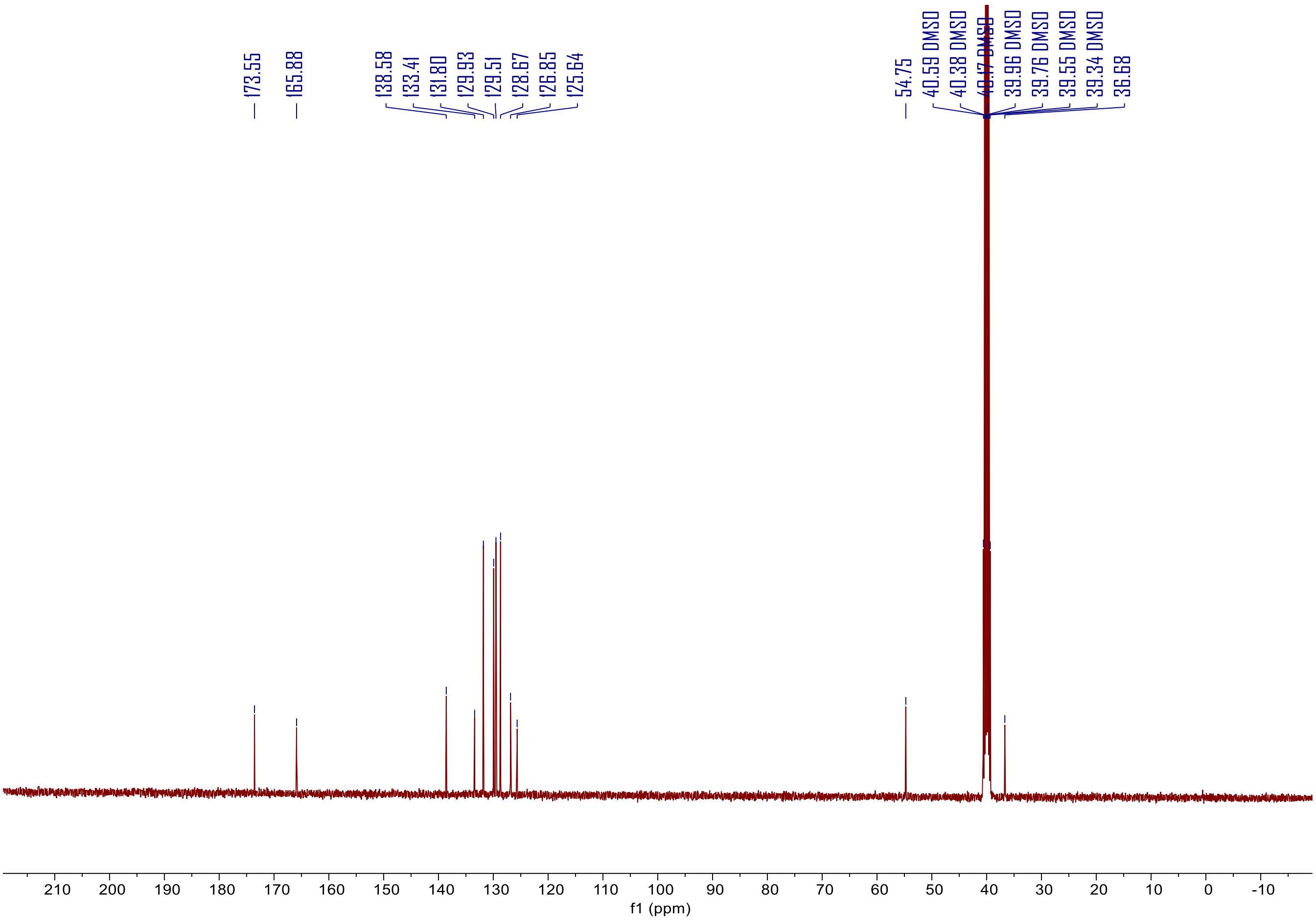

Supplementary Figure 79. ^{13}C NMR spectrum of **F-Ph-Br-D** in d-DMSO.

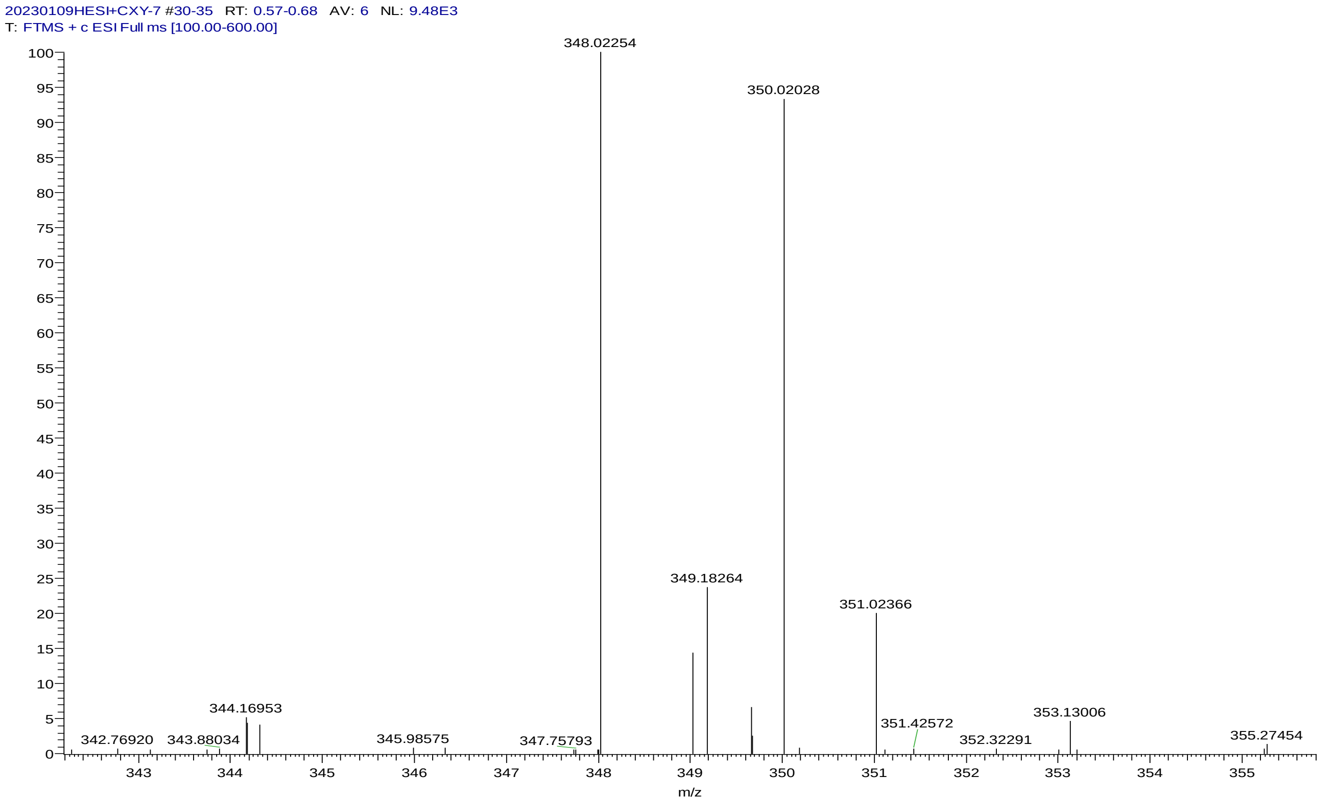

Supplementary Figure 80. EI mass spectrum of **F-Ph-Br-D**.

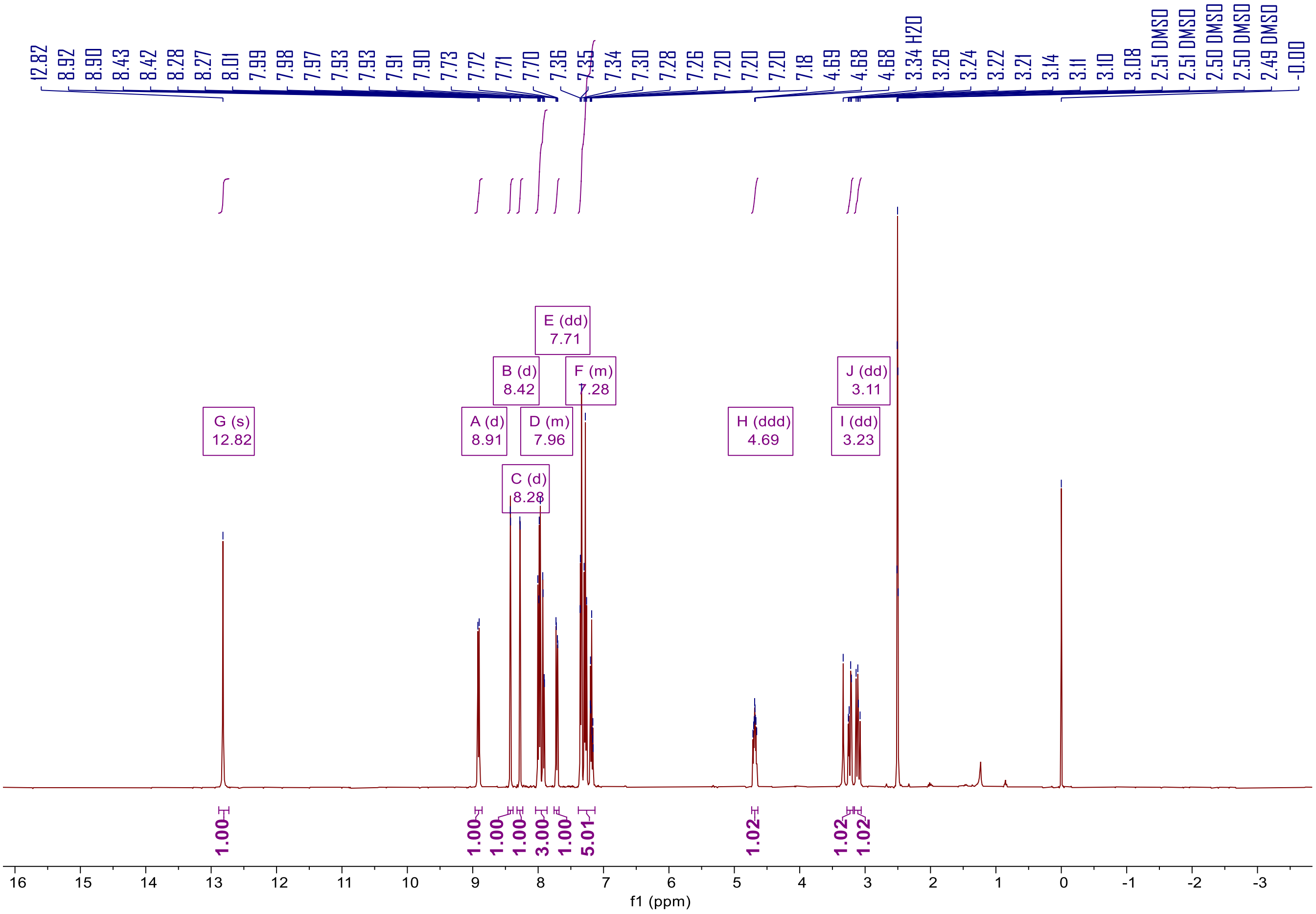

Supplementary Figure 81. ^{1}H NMR spectrum of **F-Na-Br-L** in d-DMSO.

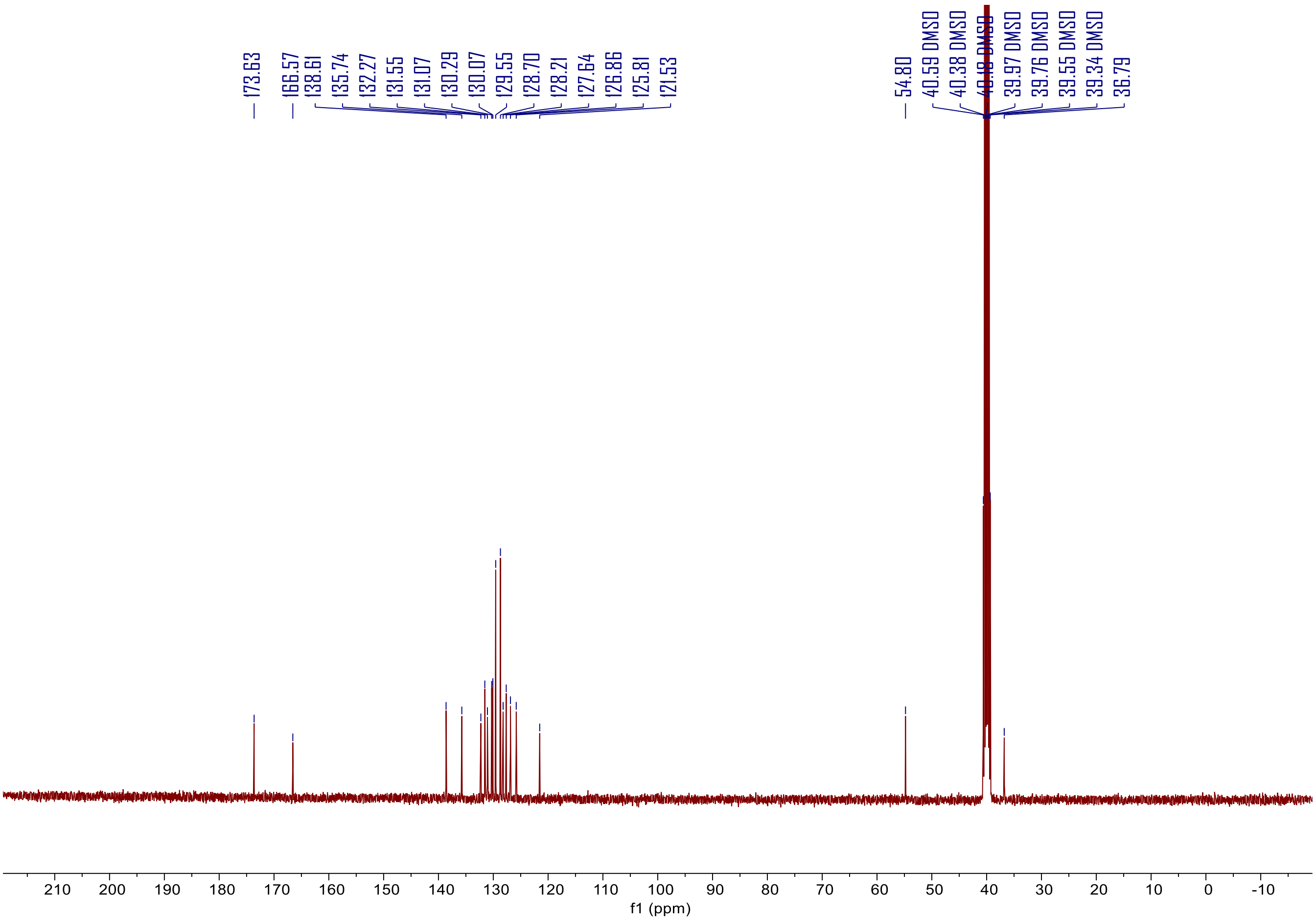

Supplementary Figure 82. ^{13}C NMR spectrum of **F-Na-Br-L** in d-DMSO.

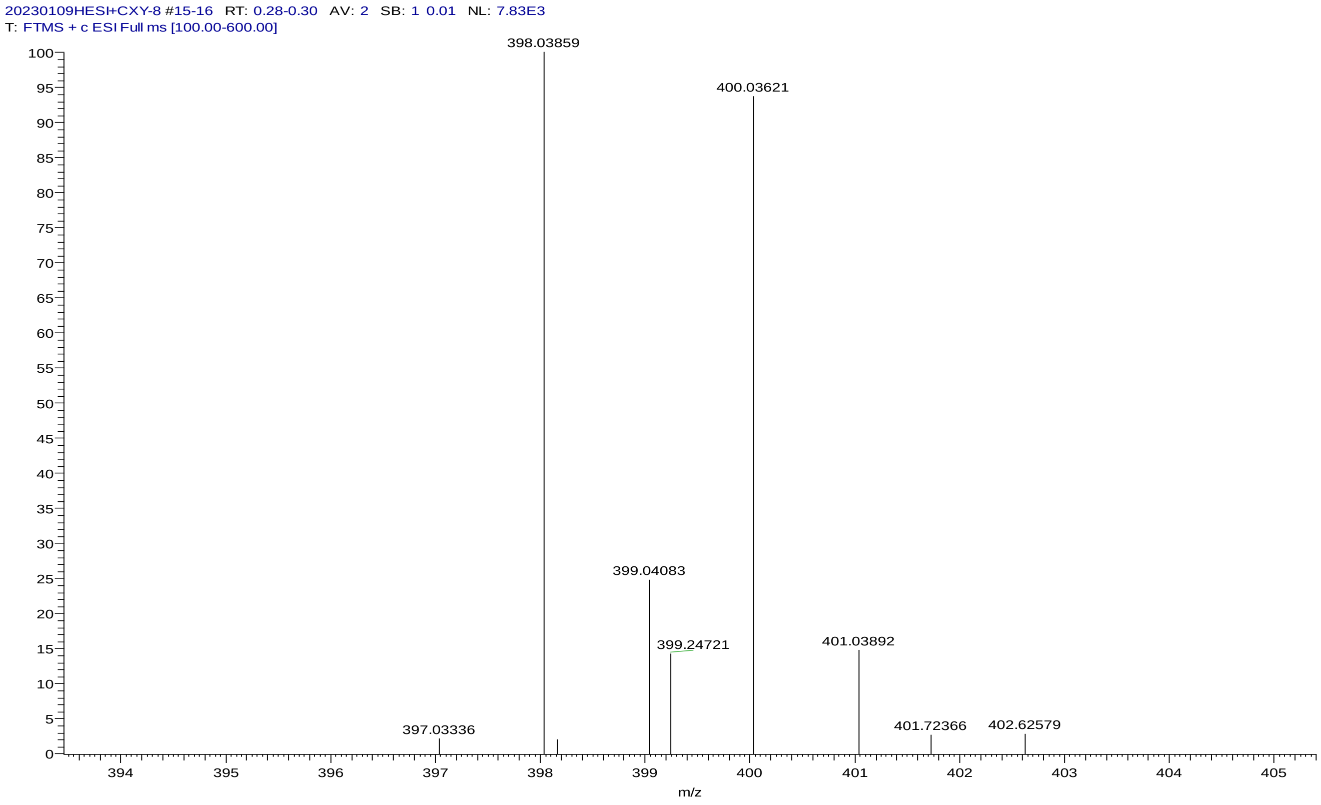

Supplementary Figure 83. EI mass spectrum of **F-Na-Br-L**.

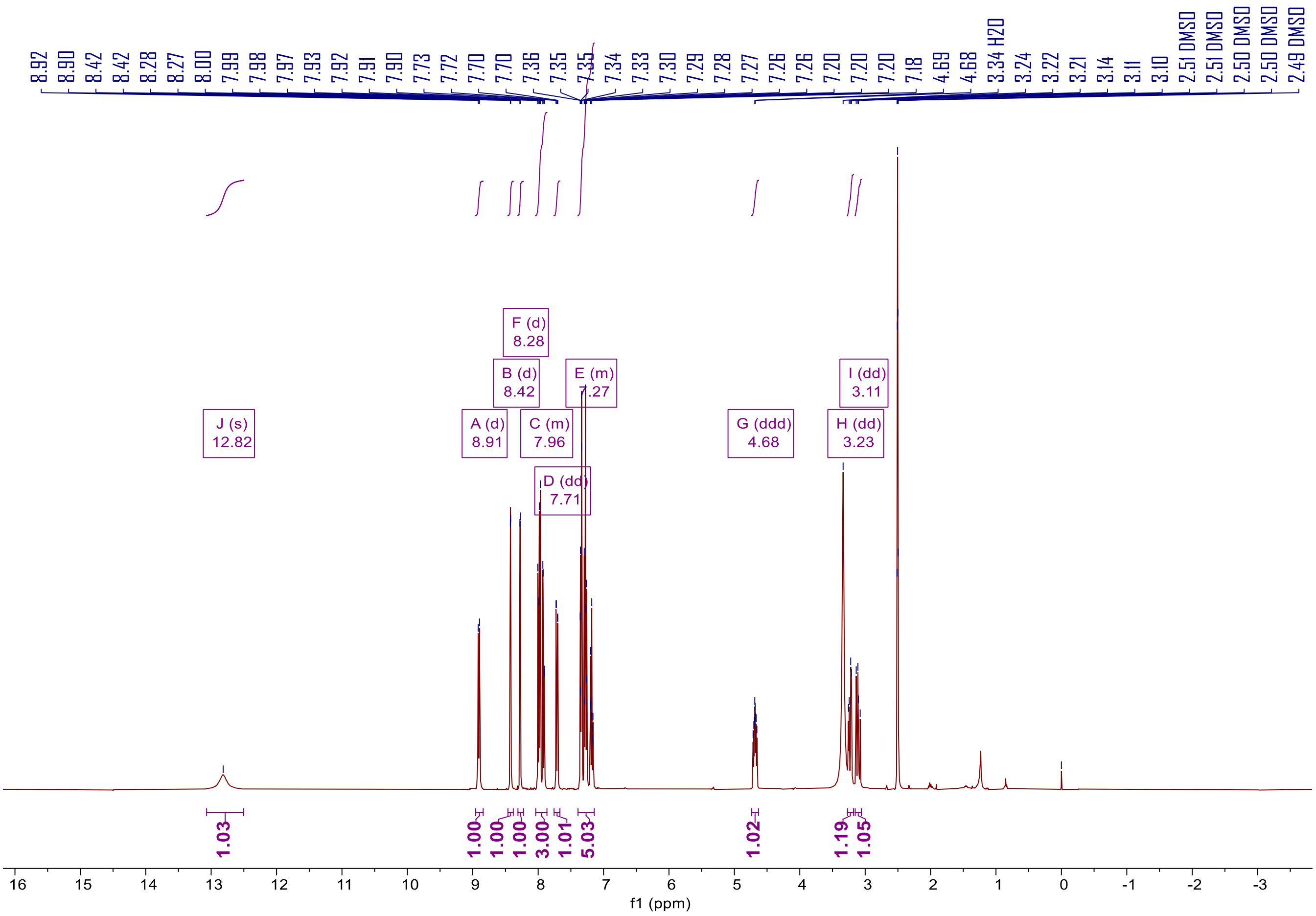

Supplementary Figure 84. ^{1}H NMR spectrum of **F-Na-Br-D** in d-DMSO.

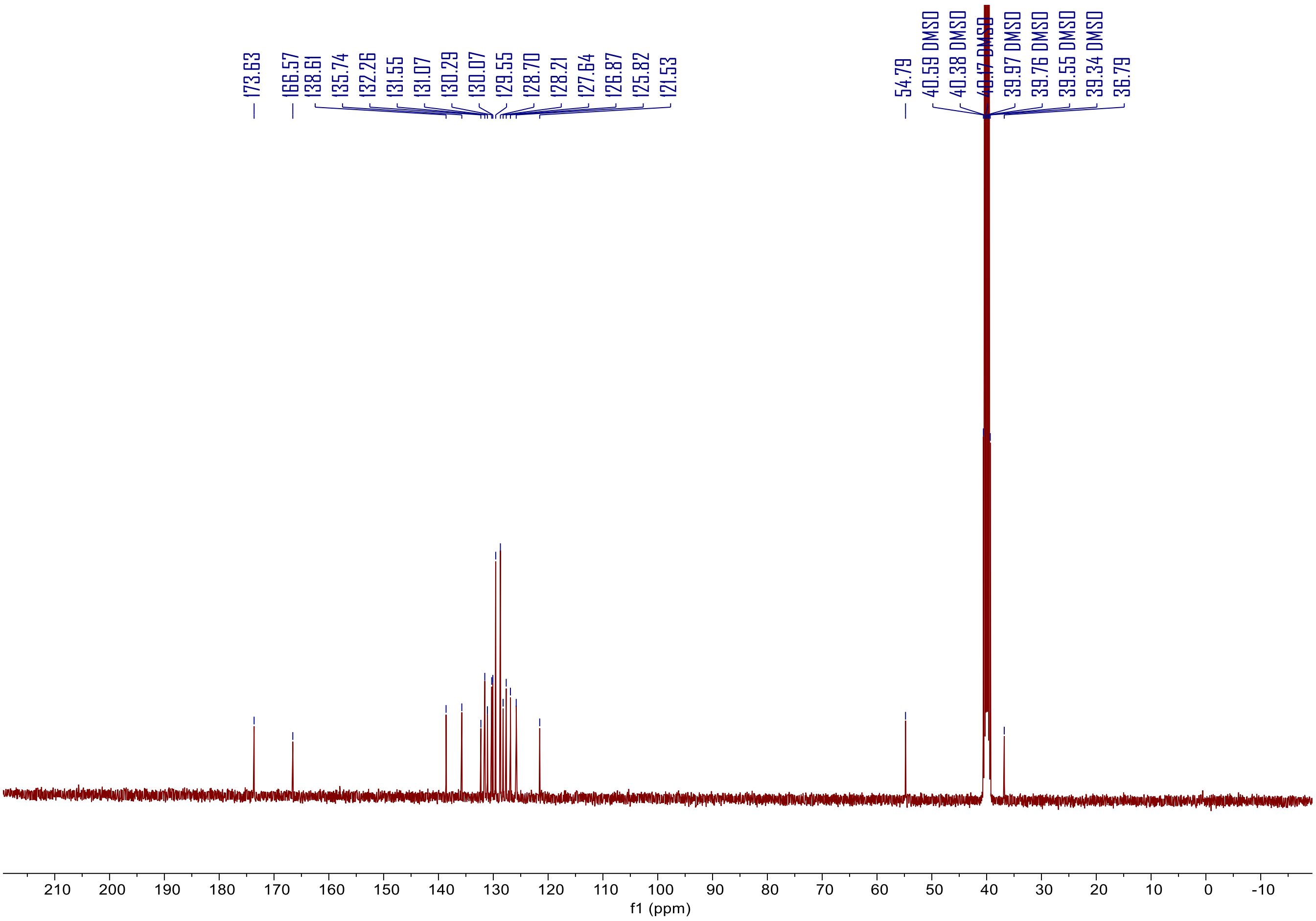

Supplementary Figure 85. ^{13}C NMR spectrum of **F-Na-Br-D** in d-DMSO.

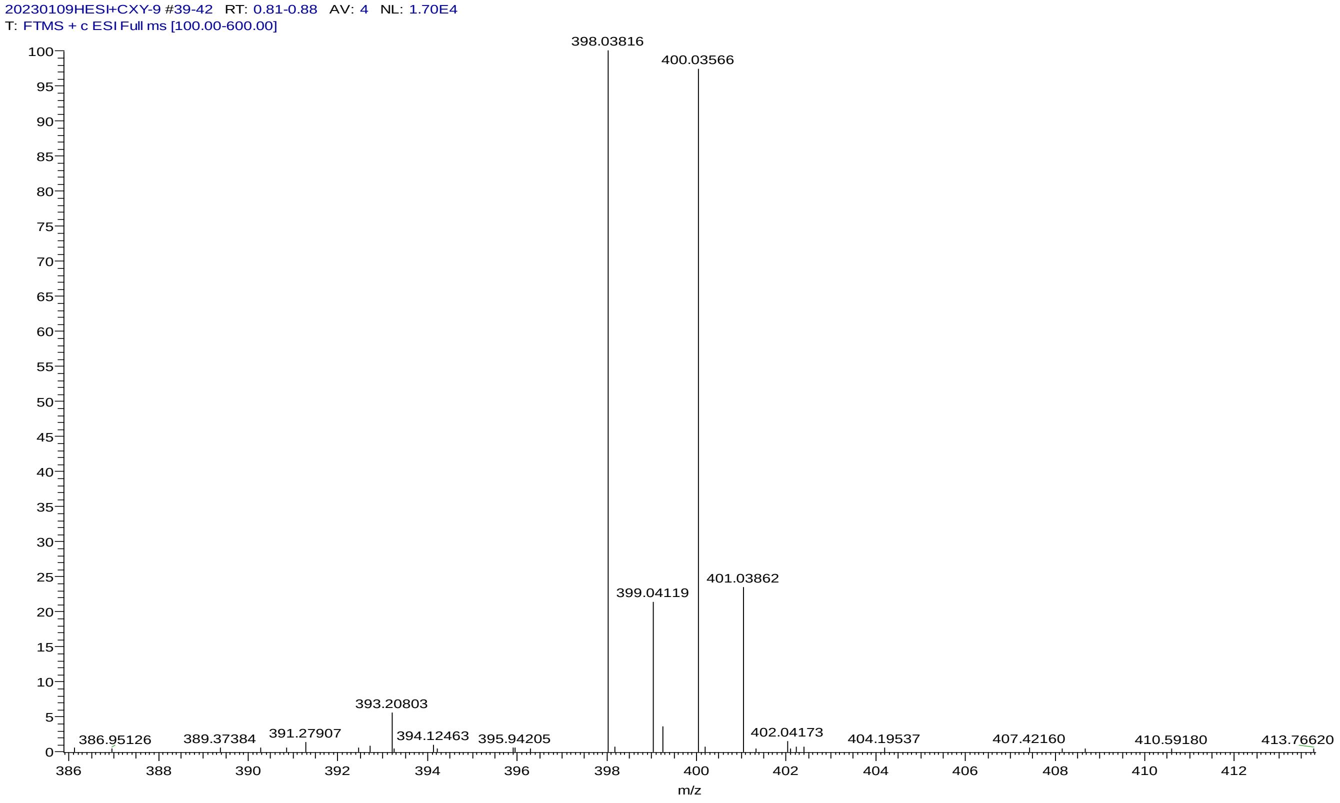

Supplementary Figure 86. EI mass spectrum of **F-Na-Br-D**.
